# N-heterocyclic carbene induced reductive coupling of phosphorus tribromide. Isolation of a bromine bridged P–P bond and its subsequent reactivity†
†Electronic supplementary information (ESI) available: All experimental data for this manuscript including analytical and computational data. CCDC 1480947–1480953. For ESI and crystallographic data in CIF or other electronic format see DOI: 10.1039/c6sc02343f



**DOI:** 10.1039/c6sc02343f

**Published:** 2016-07-20

**Authors:** Jordan B. Waters, Thomas A. Everitt, William K. Myers, Jose M. Goicoechea

**Affiliations:** a Department of Chemistry , University of Oxford , Chemistry Research Laboratory , 12 Mansfield Road , Oxford , OX1 3TA , UK . Email: jose.goicoechea@chem.ox.ac.uk

## Abstract

The room temperature reaction of a 1 : 1 mixture of phosphorus tribromide (PBr_3_) and the N-heterocyclic carbene 1,3-bis(2,6-diisopropylphenyl)-imidazol-2-ylidene (IPr) quantitatively affords the Lewis acid–base adduct (IPr)PBr_3_ (**1**). Interestingly, when **1** is heated between 55 and 65 °C for a period of several days, dark red crystals slowly begin to form in the reaction vessel accompanied by the release of bromine. The resulting crystalline sample, [P_2_(IPr)_2_Br_3_]Br ([**2**]Br), results from the reductive coupling of two equivalents of **1**, and contains a cationic moiety with a P–P bond that is bridged by a bromine atom. Anion exchange reactions with Na[BAr^F^_4_] (BAr^F^_4_ = B(3,5-{CF_3_}_2_C_6_H_3_)_4_) afford [**2**][BAr^F^_4_]. Abstraction of two equivalents of bromine allows for the isolation of the unprecedented dicationic species [P_2_(IPr)_2_Br_2_]^2+^ (**3**) which was isolated and structurally authenticated as two different [BAr^F^_4_]^–^ salts. Reaction of **2** with mild reductants such as SnBr_2_ or tetrakis(dimethylamino)ethylene (TDAE) affords [P_2_(IPr)_2_Br]^+^ (**4**) and the known radical cation [P_2_(IPr)_2_]˙^+^ (**5**), respectively. These studies show that relatively weak P–Br bonds present in compounds **1–4** can be cleaved in a straightforward manner to afford low oxidation state compounds in high yields.

## Abstract 2

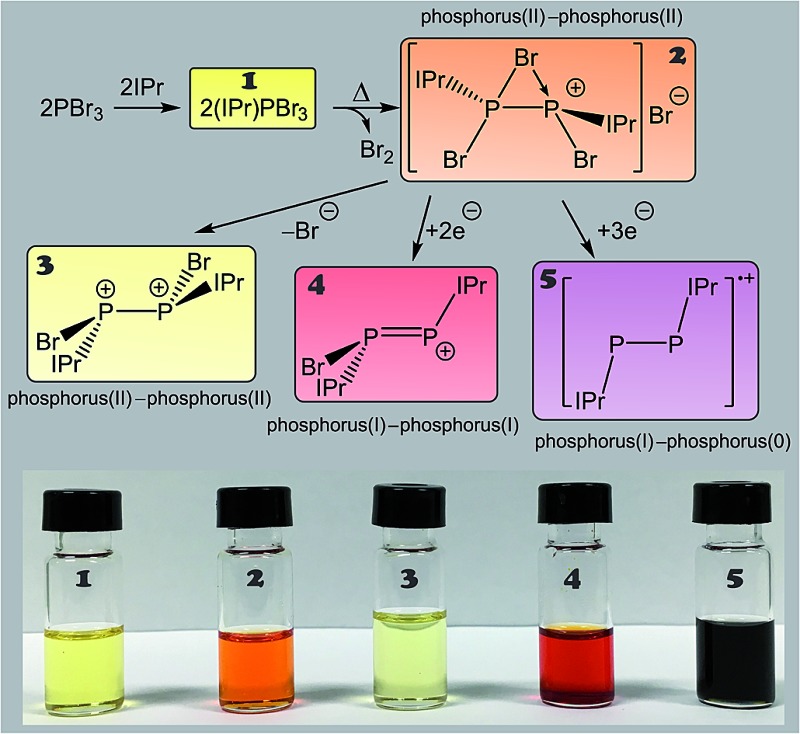
Thermal treatment of a 1:1 adduct of phosphorus tribromide and 1,3-bis(2,6-diisopropylphenyl)-imidazol-2-ylidene (IPr) results in the formation of a reduced phosphorus-containing dimer, which can be employed to access a range of low oxidation state compounds.

## 1. Introduction

The strong σ-donor properties of N-heterocyclic carbenes (NHCs), and the ease with which their steric bulk can be modified, has allowed such species to become one of the most versatile families of Lewis basic ligands in the span of just 25 years.^1–12^ NHCs have been extensively employed in main group chemistry for the isolation of low coordinate, low oxidation state compounds, such as E_2_(NHC)_2_ (E = B, Si–Sn, P, As).^13–20^ More recently, the use of cyclic alkylamino carbenes (CAACs) has also afforded related base-stabilized diatomic molecules E_2_(CAAC)_2_ (E = B, Si, P, Sb).^21–24^ Such species are typically accessed by the chemical reduction of carbene-stabilized main group element halide precursors (NHC)EX_*n*_ (*n* = 2–4) with strong reductants. As a representative example, the reduction of (NHC)PCl_3_ with three equivalents of KC_8_ allows for the isolation of P_2_(NHC)_2_ (where NHC = IPr (1,3-bis(2,6-diisopropylphenyl)-imidazol-2-ylidene) or IMes (1,3-bis(2,4,6-trimethylphenyl)-imidazol-2-ylidene)).^19^

These remarkable breakthroughs have not only challenged our understanding of chemical bonding, but have also heralded a new era in molecular main group chemistry. However, to the best of our knowledge, and despite extensive research in the area, to date there are no examples of carbene coordination spontaneously inducing the reductive coupling of such main group halides. Jones and Cole have previously demonstrated that N-heterocyclic carbenes can be used to promote the disproportionation of low oxidation state main group halides such as ‘GaI’ and InBr to afford oxidised metal centres and elemental gallium or indium, respectively.^25–27^ This technique allows access to complexes in which the group 13 elements are formally in the +2 oxidation state. The aforementioned studies indicate that due to their strong σ-donor properties, on coordination to a main group element centre, NHCs strongly influence the standard potentials of the element in question. Hence, in principle, it should be possible to use ligand coordination to increase the electron density on a given main group element halide and induce a formal reduction in oxidation state. Herein we report one such example by reaction of PBr_3_ with IPr at elevated temperature which affords a phosphorus(ii) dimer. The chemistry of this novel species towards other reductants is also explored.

## 2. Results and discussion

**Scheme 1:**
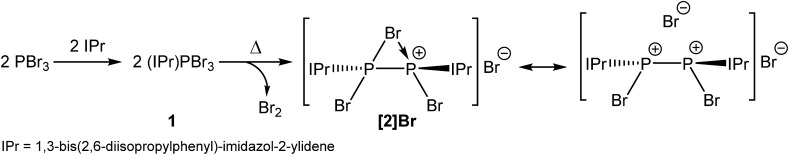
Thermally induced chemical reduction of phosphorus tribromide in the presence of an N-heterocyclic carbene to afford a bromine-bridged phosphorus(ii) dimer (**2**).

The room temperature reaction of phosphorus tribromide with IPr in diethyl ether affords the Lewis acid–base adduct (IPr)PBr_3_ (**1**; Scheme 1) which can be isolated in high yields as a compositionally pure yellow crystalline solid. The adduct exhibits a singlet resonance in its ^31^P NMR spectrum at 24.8 ppm, consistent with that observed for other carbene adducts of phosphorus trihalides (*cf.* (IPr)PCl_3_: 16.9 ppm).^28^ This resonance is shifted to a lower frequency compared to the PBr_3_ precursor (231.2 ppm). The ^1^H and ^13^C NMR spectra are also as expected for such a species.

**Fig. 1:**
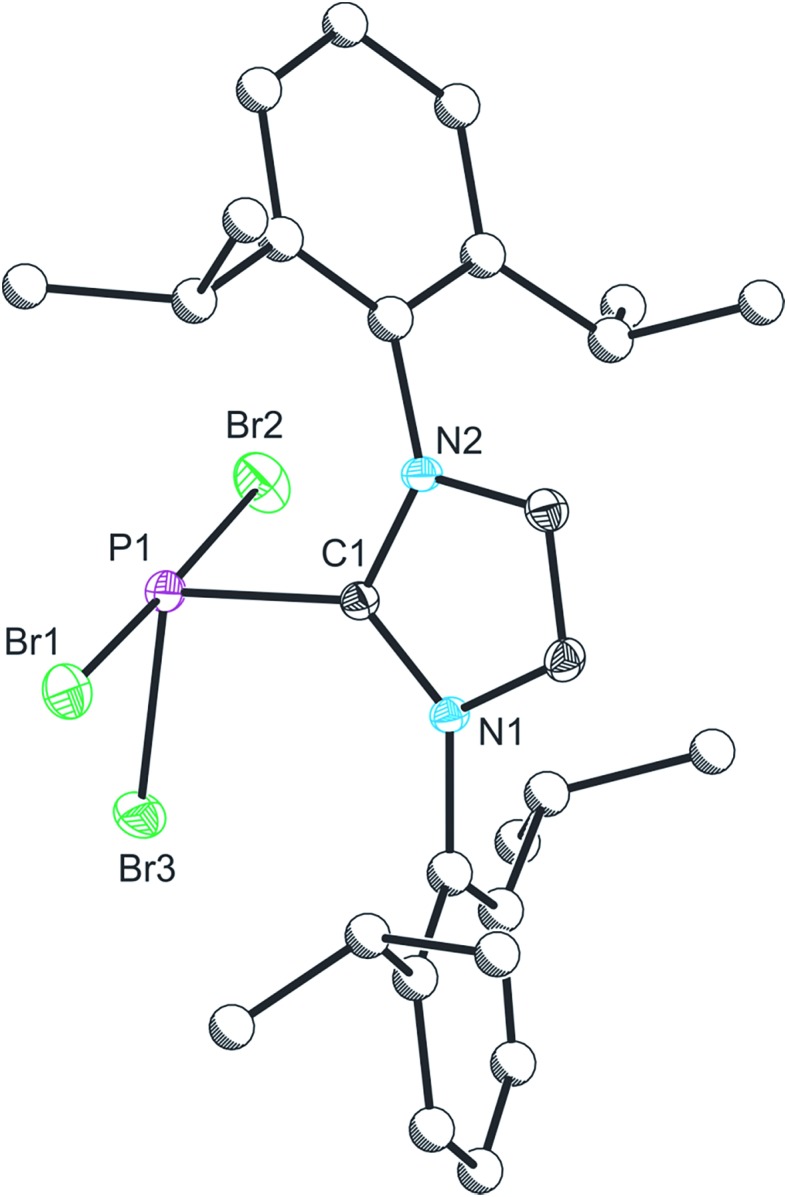
Single crystal X-ray structure of **1**. Thermal ellipsoids pictured at 50% occupancy level (carbon atoms of Dipp functionalities pictured as spheres of arbitrary radius). All hydrogen atoms removed for clarity. Selected bond distances (Å) and angles (°): P1–Br1, 2.475(1); P1–Br2, 2.545(1); P1–Br3, 2.232(1); P1–C1, 1.872(2). Br1–P1–Br2, 178.47(2); Br1–P1–Br3, 90.99(2); Br1–P1–C1, 89.41(5); Br2–P1–Br3, 90.09(2); Br2–P1–C1, 91.42(5); Br3–P1–C1, 102.64(5).

Compound **1** was characterized by single crystal X-ray diffraction. The structure exhibits a pseudo-trigonal bipyramidal geometry where the phosphorus lone pair occupies one of the equatorial positions (Fig. 1). The P1–C1 bond distance is 1.872(2) Å, and identical within experimental error to the two other structurally authenticated examples of phosphorus trihalide adducts of five membered NHC ligands ((IPr)PCl_3_: 1.871(11) Å and (NHC^Me^)PCl_3_: 1.879(2) Å; NHC^Me^ = 1,3-dimethylimidazolidin-2-ylidene).^28,29^ The P–Br distances vary significantly and, as expected, the distances to the apical bromine atoms (2.475(1) and 2.545(1) Å) are notably longer than that observed to the equatorial position (2.232(1) Å). This latter bond length falls within the range expected for a P–Br single bond (2.25–2.27 Å),^30,31^ whereas the bonds to the apical positions are significantly weakened, a result of the donation of the carbene lone pair into a P–Br σ* orbital.

Interestingly, when a solution of PBr_3_ and IPr is heated between 55 and 65 °C in tetrahydrofuran (THF), large dark red crystals slowly form in the reaction vessel. This novel compound arises from the reductive coupling of two equivalents of **1** with concomitant release of bromine (Br_2_) to afford [P_2_(IPr)_2_Br_3_]^+^ (**2**) as a bromide ion salt (Scheme 1). NMR spectroscopy reveals quantitative consumption of **1** over the course of 72 hours allowing for the isolation of [**2**]Br·3THF in high crystalline yields (approx. 70%). It is also important to note at this stage that thermal treatment of THF solutions of **1** also give rise to [**2**]Br. To our knowledge this is the first example of the thermally induced reductive coupling of a main group element halide. This process is facilitated by the strong electron donation of the N-heterocyclic carbene to the phosphorus(iii) centre in **1**, which significantly weakens the P–Br bonds. The electronic and steric stabilization offered by the two IPr ligands serve to stabilize what can be interpreted as a base-stabilized cyclic bromonium ion, or conversely, and perhaps more accurately, a dicationic species [P_2_(IPr)_2_Br_2_]^2+^ which forms a strong electrostatic interaction with a bromide ion (*vide infra*). The chlorine-containing analogue of **2**, [P_2_(IPr)_2_Cl_3_]^+^, was recently reported by Wolf, Weigand and co-workers from the chemical reduction of [(IPr)PCl_2_]OTf with sodium metal.^32^

The composition of **2** was corroborated by means of positive ion mode electrospray ionization mass-spectrometry (ESI-MS) which revealed the presence of the molecular ion ([P_2_(IPr)_2_Br_3_]^+^) at 1079.2753 (calculated value: 1079.2739). The ^31^P NMR spectrum of [**2**]Br·3THF in CD_2_Cl_2_ reveals a singlet at –27.3 ppm indicative of two equivalent phosphorus environments, whereas the ^1^H and ^13^C NMR spectra of the compound are consistent with restricted rotation of the N-heterocyclic carbene functionalities about the P–C bonds.

Also worth noting at this stage is that thermal treatment of a solid sample of **1** under a static vacuum at 140 °C (with occasional evacuation of the reaction vessel headspace) ultimately affords the bromine bridged species [**2**]Br, which is consistent with the loss of Br_2_. We have established this by means of ^31^P NMR spectroscopy which indicates that the predominant product is [**2**]Br with some trace amounts of other phosphorus-containing impurities (the main one of which is PBr_3_; see ESI† for spectra). A thermal gravimetric analysis (TGA) of **1** also shows loss of Br_2_ on heating at 140 °C.

**Fig. 2:**
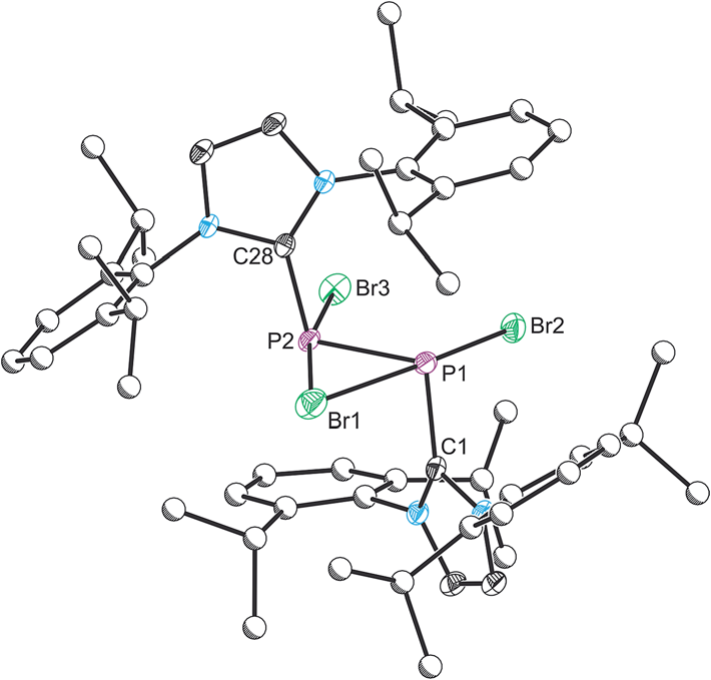
Single crystal X-ray structure of the cationic component in [**2**]Br·3THF. Thermal ellipsoids pictured at 50% occupancy level (carbon atoms of Dipp functionalities pictured as spheres of arbitrary radius). All hydrogen atoms removed for clarity. Selected bond distances (Å) and angles (°): P1–P2, 2.252(1); P1–Br1, 2.667(1); P1–Br2, 2.349(1); P2–Br1, 2.810(1); P2–Br3, 2.288(1); P1–C1, 1.866(3); P2–C28, 1.860(3). Br1–P1–Br2, 172.08(3); Br1–P1–P2, 69.09(2); Br1–P1–C1, 86.53(8); Br2–P1–C1, 92.45(9); Br1–P2–Br3, 168.08(3); Br1–P2–C28, 87.81(8); Br3–P2–C28, 95.00(8); P2–P1–Br2, 103.41(3); P1–P2–Br3, 105.65(3); C1–P1–P2, 100.08(9); P1–P2–C28, 100.62(9).

The structure of **2** was established by means of single crystal X-ray diffraction (Fig. 2) and reveals a planar [P_2_Br_3_]^+^ moiety (mean deviation from planarity 0.0184 Å) in which one of the bromine atoms bridges the P–P bond. There is an IPr ligand associated with each phosphorus centre with P–C interatomic distances of 1.866(3) and 1.860(3) Å (*cf.* 1.872(2) in **1**). The two carbene ligands sit on opposite sides of the plane defined by the [P_2_Br_3_]^+^ core. The interatomic distance between the two phosphorus atoms in **2** is 2.252(1) Å and fully consistent with a P–P single bond (2.14 to 2.22 Å). The bridging bromine atom is largely equidistant from the two phosphorus atoms (2.667(1) and 2.810(1) Å). These distances are notably longer than those observed between the phosphorus centres and the terminal bromine atoms to which they are bonded (2.349(1) and 2.288(1) Å). The isolobal and diagonal relationship between a phosphorus atom and a methine (C–H) group allows for the structure of **2** to be interpreted as a phosphorus-containing analogue of a bromonium ion. That being said, previous computational studies on the chlorine-containing analogue, [P_2_(IPr)_2_Cl_3_]^+^, indicated that the interaction between the bridging halide atom and the two phosphorus centres is more consistent with an electrostatic interaction between an anionic halide ion and a dicationic [P_2_(IPr)_2_Cl_2_]^2+^ core. The low Wiberg bond indices between the phosphorus centres and the bridging chlorine atom (0.25), the relatively high negative charge associated with the bridging chlorine, and an AIM topological analysis were all employed to reach this conclusion.^32^

Compound **2** can also be accessed by reduction of **1** with KC_8_, however such reactions are difficult to control and give rise to a mixture of products (over reduction is a significant issue). By contrast, thermal treatment of **1** affords **2** in high crystalline yields and does not require the use of strong reductants. To the best of our knowledge this represents a unique methodology to access low valent phosphorus compounds. Presumably this arises due to the weaker P–Br bonds when compared to P–Cl, and the reduced oxidative character of Br_2_ relative to Cl_2_. Salt metathesis reactions between [**2**]Br and Na[BAr^F^_4_] or Tl[BAr^F^_4_] in THF allow for the exchange of the bromide ion to afford [**2**][BAr^F^_4_], but even when an excess of these salts is employed the bridging bromine atom cannot be displaced. This effect was found to be strongly solvent dependent however, and similar anion exchange reactions in 1,2-diflurorobenzene (DFB) are markedly different (*vide infra*). Crystallographic verification of the structure of [**2**][BAr^F^_4_] was also obtained and the bond metric data are comparable to that of [**2**]Br·3THF (these data are provided in the ESI†). Exchanging the anion associated with **2** significantly varies the solubility of the salts, thus while [**2**]Br·3THF is essentially insoluble in ethereal solvents such as THF and Et_2_O, [**2**][BAr^F^_4_] is notably more soluble in common laboratory solvents.

**Fig. 3:**
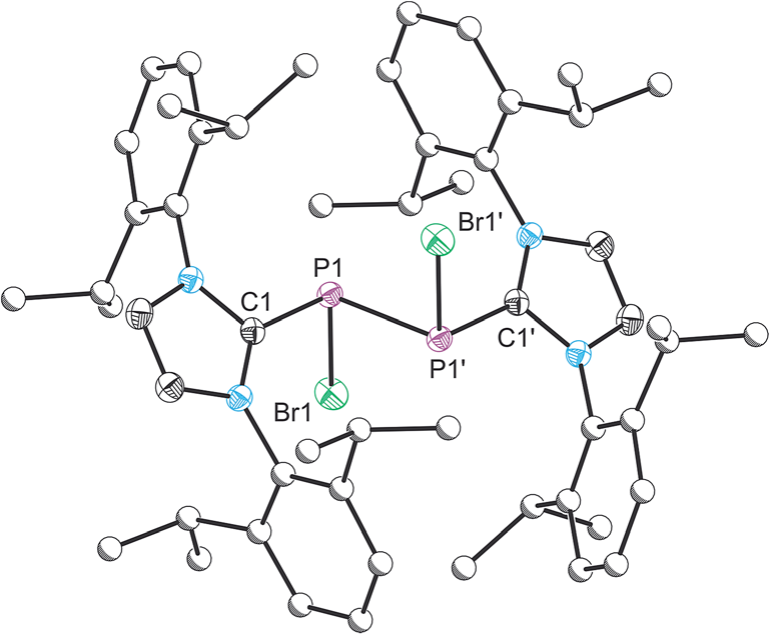
Single crystal X-ray structure of one of the two cationic components in [**3**][BAr^F^_4_]_2_·1.5CH_2_Cl_2_. Thermal ellipsoids pictured at 50% occupancy level (carbon atoms of Dipp functionalities pictured as spheres of arbitrary radius). All hydrogen atoms removed for clarity. Selected bond distances (Å) and angles (°): P1–P1′, 2.232(1); P1–Br1, 2.213(1); P1–C1, 1.850(2). Br1–P1–P1′, 94.46(4); C1–P1–P1′, 98.15(8); C1–P1–Br1, 101.88(8). Symmetry operation: 1 – *x*, –*y*, 2 – *z*.

**Scheme 2:**
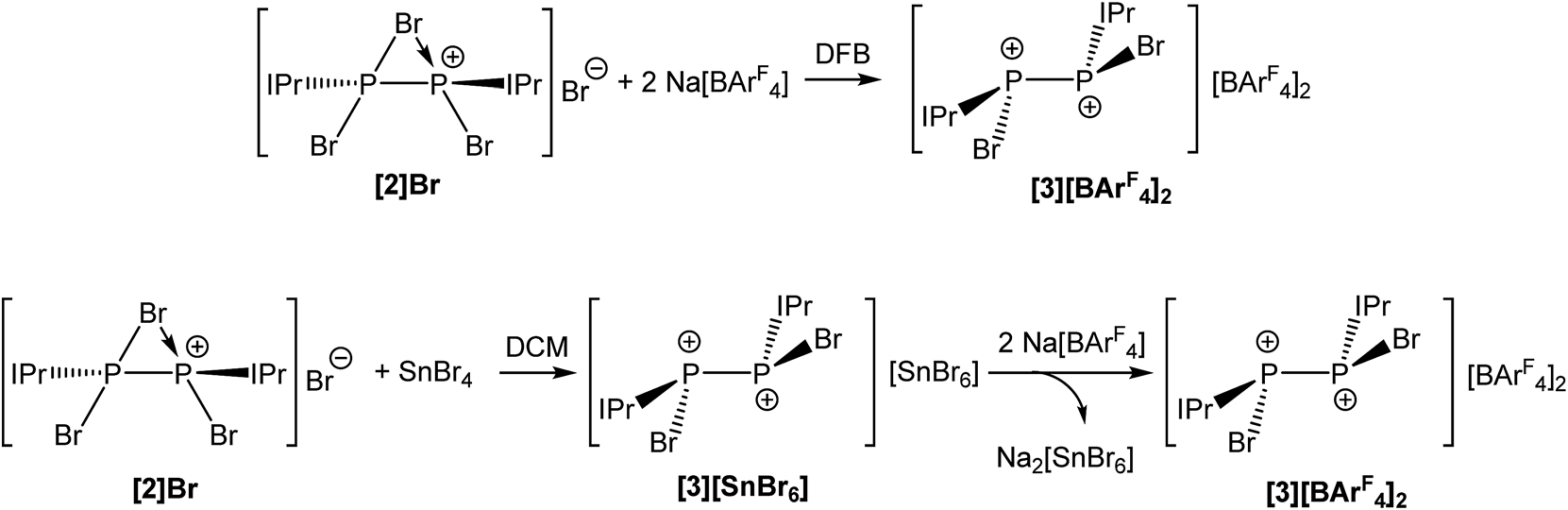
Reaction of [**2**]Br with either Na[BAr^F^_4_] in DFB (top) or SnBr_4_ in DCM (bottom) to afford [**3**][BAr^F^_4_]_2_.

Abstraction of an additional bromide ion was possible by reaction of [**2**]Br with two equivalents of Na[BAr^F^_4_] in 1,2-difluorobenzene (DFB), which is in stark contrast with the reactivity observed in THF. This indicates that in polar donor solvents, solvation of the alkali metal cation is a sufficiently significant thermodynamic force to prevent metathesis. This reaction affords the novel dicationic species [P_2_(IPr)_2_Br_2_]^2+^ (**3**) as [BAr^F^_4_]^–^ salt (Fig. 3). In dichloromethane (DCM), the bridging bromide ion can be removed by reaction of [**2**]Br with one equivalent of SnBr_4_ affording **3** as a SnBr_6_^2–^ salt. Anion exchange using Na[BAr^F^_4_] allowed for the isolation of [**3**][BAr^F^_4_]_2_ as a yellow crystalline solid as pictured in Scheme 2. Both [**3**][SnBr_6_] and [**3**][BAr^F^_4_]_2_ are sparingly soluble in DCM, consequently anion exchange requires relatively long reaction times and results in the generation of Na_2_[SnBr_6_] as a side-product (separation of a crystalline mixture of [**3**][BAr^F^_4_]_2_ and Na_2_[SnBr_6_] was carried out manually based on the different colour and morphology of the crystals). Consequently, the most efficient method for the generation of [**3**][BAr^F^_4_]_2_ is from reaction of **2** with two molar equivalents of Na[BAr^F^_4_] in DFB (Scheme 2).

Due to its poor solubility in most common laboratory solvents, NMR spectra for [**3**][BAr^F^_4_]_2_ were collected in DFB and reveal a singlet resonance at –1.8 ppm in the ^31^P NMR spectrum.

**Scheme 3:**
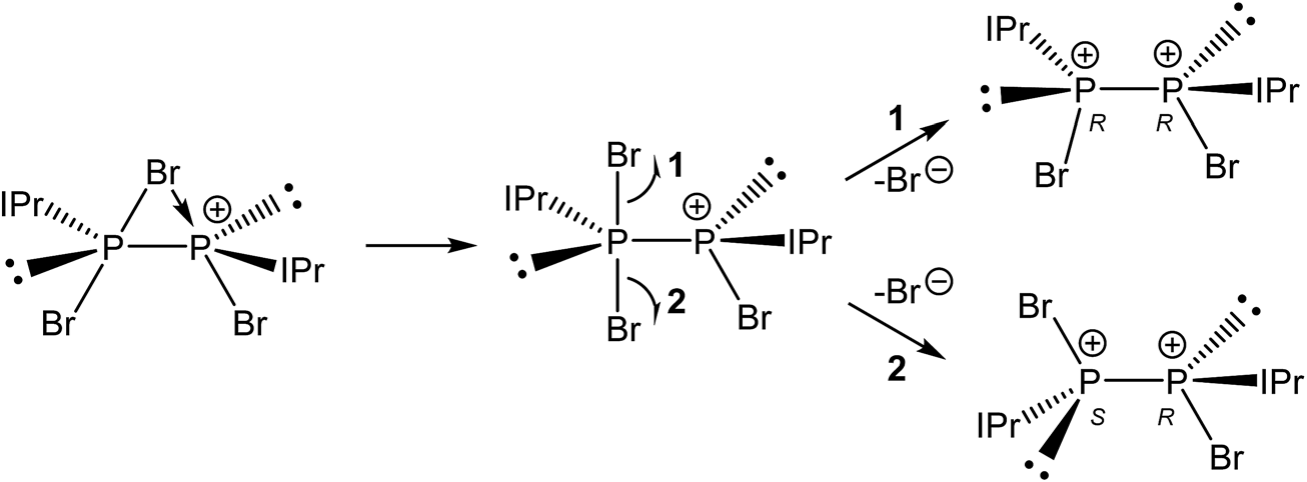
Possible stereochemical outcomes from the bromide ion abstraction from **2** to afford **3**.

Compound **3** was structurally characterized by single crystal X-ray diffraction in [**3**][BAr^F^_4_]_2_·2DFB and [**3**][BAr^F^_4_]_2_·1.5CH_2_Cl_2_. Both solvates exhibit comparable bond metric data (a comparison is provided in the ESI†). All of the dicationic moieties characterized reveal an anticlinal arrangement of the bromine atoms, with the carbenes in antiperiplanar positions (in other words, there is a centre of inversion along the P–P bond, rendering the two phosphorus centres enantiomeric (1*S*,2*R*)). At no point during our studies did we observe evidence for the formation of the stereoisomers 1*R*,2*R* or 1*S*,2*S* (*i.e.* systems in which there is a synclinal arrangement of either the bromine atoms or the carbene substituents). This observation is rather significant with regard to the mechanism of bromide ion abstraction from [**2**]Br. Loss of the bridging bromide can only afford the 1*R*,2*R*/1*S*,2*S* enantiomeric pair unless there is a pyramidal inversion at one of the phosphorus centres, a process which is known to be energetically costly (typically requiring >80 kJ mol^–1^).^33–36^ The other possibility is that a terminal bromide ion is being abstracted from [**2**]Br, perhaps *via* a mechanism where the bridging bromide ion adopts a terminal position and the bromide *trans* to it is lost. This is exemplified in Scheme 3. Density functional theory (DFT) level calculations reveal that the 1*S*,2*R* isomer is 29.0 kJ mol^–1^ more stable that the 1*R*,2*R* stereoisomer, indicating that this may well be a thermodynamically favoured phenomenon.

For clarity, we will only discuss bond metric data for one of the two structurally authenticated samples of **3**, [**3**][BAr^F^_4_]_2_·1.5CH_2_Cl_2_. The structure contains two crystallographically independent [P_2_(IPr)_2_Br_2_]^2+^ moieties in the lattice. The P–P bond distance is 2.232(1) Å, which is marginally shorter that that observed for the parent compound **2** (2.252(1) Å), but still largely consistent with a P–P single bond. The sum of bond angles around the phosphorus centre (294.49°) is as expected for a pyramidal phosphorus centre possessing a stereochemical lone pair of electrons. The loss of the bridging bromide ion on going from **2** to **3** results in a modest reduction in the P–C_carbene_ bond lengths to 1.850(2) Å (from 1.866(3) and 1.860(3) Å in **2**). The P–Br bond lengths, however, are more dramatically affected, and experience a significant reduction on removal of the bridging bromide ion to 2.213(1) Å (from 2.349(1) and 2.288(1) Å in **2**). The similarity of the P–P and P–C bond metric data between **2** and **3** indicate that the bonding in both species is very similar, and that therefore the formulation of **2** as a dicationic species that is bridged by a bromide ion is most appropriate. Compound **3** is an unprecedented phosphorus(ii)–phosphorus(ii) dication, and is isoelectronic with a neutral silicon(i) dimer, Si_2_(IPr)_2_Br_2_, reported by Filippou and co-workers.^37^ It is worth noting however, that for Si_2_(IPr)_2_Br_2_ only the *RR*/*SS* stereoisomers were observed.

**Scheme 4:**
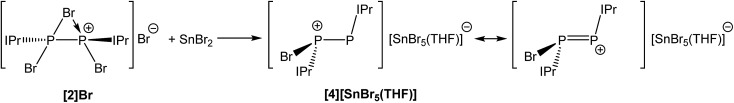
Reduction of [**2**]Br with SnBr_2_ to afford [**4**][SnBr_5_(THF)]. Note in solution [SnBr_5_(THF)]^–^ gives rise to an equilibrium with SnBr_4_ and [SnBr_6_]^2–^. Consequently, **4** was structurally authenticated as the hexabromostannate salt [**4**]_2_[SnBr_6_].

The fact that [**2**]Br can be accessed without a reducing agent, and that coordination of the N-heterocyclic carbene IPr to PBr_3_ allowed for the thermally induced reductive coupling of phosphorus(iii) to phosphorus(ii), indicates that the P–Br bonds are relatively weak and that subsequent reduction of the phosphorus centres in **2** should be possible using mild reductants. A two electron reduction of [**2**]Br with one equivalent of SnBr_2_ in THF results in the formation of [P_2_(IPr)_2_Br]^+^ (**4**) and SnBr_4_. The SnBr_4_ goes on to further react with the bromide ions present in solution to afford SnBr_5_^–^. The net stoichiometric reaction is depicted in Scheme 4.

The reaction was monitored by ^31^P NMR spectroscopy and revealed near quantitative conversion of **2** to **4** as evidenced by the appearance of two doublets at 145.4 and –7.6 ppm with a ^1^ *J*_P–P_ coupling constant of 391 Hz. The two inequivalent phosphorus environments and their chemical shifts are consistent with the formation of a compound with a [(IPr)P–P(IPr)Br]^+^ core, with the highest frequency resonance (145.4 ppm) corresponding to the two-connect phosphorus centre. Compound **4** can be interpreted as either a phosphorus(i)–phosphorus(i) dimer or as a mixed valence species with the phosphorus atoms in the +2 and 0 oxidation states (depending on which of the two principal resonance forms is invoked (see Scheme 4)). Variable temperature NMR studies reveals fluxional behaviour that leads to the exchange of the heterotopic phosphorus nuclei. This fluxionality was observed in a similar silicon(i) species (Si_2_(IPr_2_)I^+^) reported by Filippou and co-workers.^37^ On heating to 338 K, the phosphorus resonances in the ^31^P NMR spectrum broaden beyond recognition, although on cooling to 208 K, the two doublets progressively sharpen due to the decreasing rate of exchange of the bromide between the two phosphorus centres. The ^1^H NMR spectrum at 208 K shows two distinct IPr environments whereby one IPr ligand has restricted rotation about the carbon–phosphorus bond. This is clearly seen in the imidazole resonances. One of the NHC ligands displays two distinct singlets at 8.24 and 8.20 ppm which each integrate to one proton, demonstrating the asymmetry of the ligand at this temperature. A third resonance at 7.80 ppm (integrating to two protons) is attributed to the imidazole protons of the second, freely rotating IPr. Likewise, the ^13^C{^1^H} NMR spectrum of [**4**][SnBr_5_(THF)] at 208 K shows the inequivalence of the two IPr ligands. Most characteristically, two doublet of doublet resonances which appear at 164.2 and 150.6 ppm can be assigned to the two separate carbene carbons. The chlorine-containing analogue of **4**, [P_2_(IPr)_2_Cl]^+^, has previously been reported by Wolf and Weigand *via* reduction of [(IPr)PCl_2_]OTf at –90 °C which afforded a mixture of products.^32^ By contrast, this novel synthesis takes place at room temperature and is, as previously mentioned, near quantitative.

**Fig. 4:**
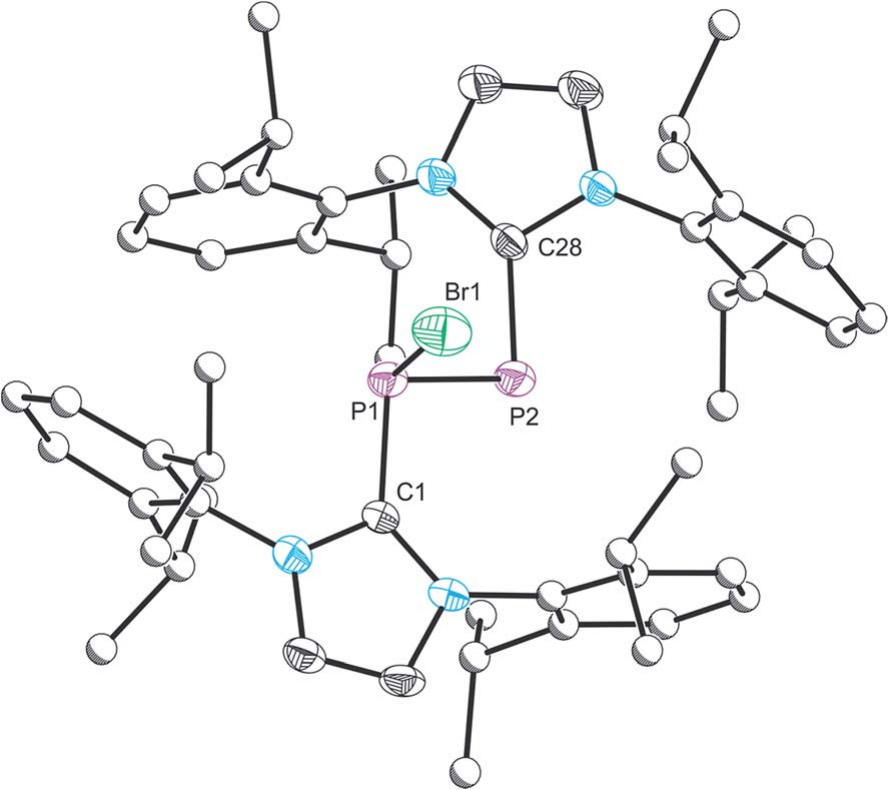
Single crystal X-ray structure of one of the two cationic components in [**4**]_2_[SnBr_6_]·THF. Thermal ellipsoids pictured at 50% occupancy level (carbon atoms of Dipp functionalities pictured as spheres of arbitrary radius). All hydrogen atoms removed for clarity. Selected bond distances (Å) and angles (°): P1–P2, 2.096(2); P1–Br1, 2.443(1); P1–C1, 1.847(5); P2–C28, 1.845(5). Br1–P1–P2, 109.29(6); Br1–P1–C1, 93.02(15); C1–P1–P2, 100.99(16); P1–P2–C28, 96.39(16).

Compound **4** was structurally authenticated in [**4**]_2_[SnBr_6_]·THF and reveals two molecules in the asymmetric unit accompanied by a SnBr_6_^2–^ dianion and a molecule of THF (Fig. 4). Bond metric data are comparable for both cations and a comparison is provided in the ESI.† The P–P bond distance is 2.096(2) Å which is notably shorter than that observed for **2** (2.252(1) Å) and **3** (2.232(1) Å). The significant shortening of this bond, which is of a comparable magnitude to literature reported species with a P=P double bond, indicates that there is significant contribution to bonding by the resonance structure carrying a formal positive change on the two-connect phosphorus atom (*i.e.* the phosphorus(i)–phosphorus(i) species). That being said the computed Hirshfeld charges show that the greatest degree of positive charge accumulates on the phosphorus centre bonded to the bromine atom (0.143), indicating that both resonances structures must contribute significantly to the structure of **4**. The P–C bond distances to the NHC ligands are identical within experimental error (1.847(5) and 1.845(5) Å) and slightly shorter than those observed for **2** (1.866(3) and 1.860(3) Å). In contrast, the P–Br bond, 2.443(1) Å, is notably longer than the analogous distances in **2** (2.349(1) and 2.288(1) Å) and **3** (2.213(1) Å) indicating a weak interaction, and suggesting that bromide ion abstraction to afford the phosphorus(i)–phosphorus(i) dication, [P_2_(IPr)_2_]^2+^, may be possible. In fact, ^31^P NMR spectroscopy suggests that when a solution of [**2**]Br and SnBr_2_ is allowed to sit for prolonged periods of time the dicationic species [P_2_(IPr)_2_][SnBr_6_] is ultimately generated from such mixtures as evidenced by the appearance of a resonance at 442.6 ppm in the ^31^P NMR spectrum of the reaction mixture. This secondary halide abstraction can be circumvented by the addition of half an equivalent of IPr in order to sequester SnBr_4_ as the Lewis acid–base adduct (IPr)SnBr_4_ resulting in the crystallisation of [**4**]_2_[SnBr_6_]·THF.

**Scheme 5:**
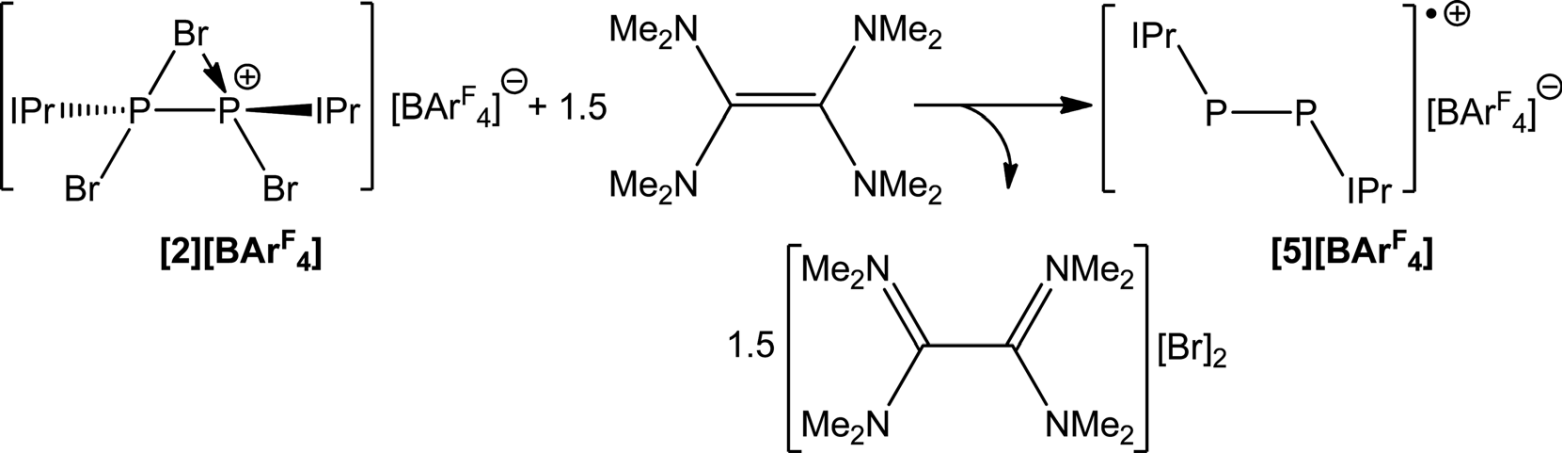
Reduction of [**2**][BAr^F^_4_] with TDAE to afford [**5**][BAr^F^_4_].

The use of a stronger reductant than SnBr_2_, such as tetrakis(dimethylamino)ethylene (TDAE) results in a three electron reduction of **2** to afford the known cationic species [P_2_(IPr)_2_]˙^+^ (**5**). This reaction affords the radical cation in quantitative yields when 1.5 equivalents of TDAE are employed (Scheme 5). When monitoring the reaction by ^31^P NMR spectroscopy, it is clear that the use of less than 1.5 equivalents of TDAE results in incomplete consumption of **2**. Expectedly, the use of a stoichiometric excess of TDAE has no effect and full conversion to **4** is observed with greater stoichiometric loadings. Compound **5** was first reported by Bertrand and co-workers by the chemical oxidation of P_2_(IPr)_2_ with [CPh_3_][B(C_6_F_5_)_4_], which can be further oxidized to afford a closed shell dicationic species.^38^ Bertrand and co-workers have also isolated a related monocation stabilized by CAAC ligands.

Room temperature X-band (*ν* = 9.3761 GHz) electron paramagnetic resonance (EPR) spectroscopy on a 100 μM solution of [**5**][BAr^F^_4_] in fluorobenzene reveals that the predominant spin density is located on the ^31^P nuclei, giving rise to 1 : 2 : 1 hyperfine pattern (*g*_iso_ = 2.0090 (+/– 0.0001); as previously reported by Bertrand and co-workers). The isotropic hyperfine for the ^31^P nuclei, *A*_iso_(^31^P), is 126 MHz. The hyperfine interactions of the four ^14^N atoms of the imidazolyl groups are resolved as a nine-peak pattern on the central peak. The ^14^N isotropic hyperfine, *A*_iso_(^14^N), is 4 MHz (full details of the EPR spectrum are provided in the ESI†).

**Fig. 5:**
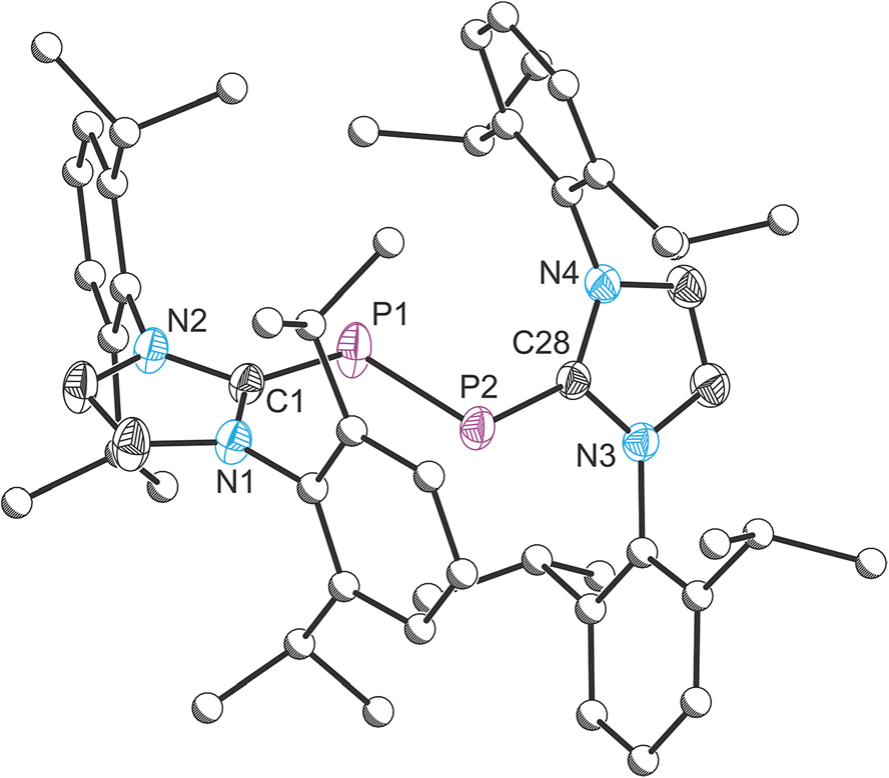
Single crystal X-ray structure of the cationic moiety in [**5**][BAr^F^_4_]. Thermal ellipsoids pictured at 50% occupancy level (carbon atoms of Dipp functionalities pictured as spheres of arbitrary radius). All hydrogen atoms removed for clarity. Selected bond distances (Å) and angles (°): P1–P2, 2.111(1); P1–C1, 1.795(2); P2–C28, 1.824(2). C1–P1–P2, 104.07(6); P1–P2–C28, 95.48(5).

Compound **5** was structurally authenticated by single crystal X-ray diffraction as [**5**][BAr^F^_4_] (Fig. 5). The bond metric data are entirely consistent with that reported by Bertrand and co-workers for their [B(C_6_F_5_)_4_]^–^ salt. Thus the P–P bond distance is 2.111(1) Å (*cf.* 2.091(1) Å in Bertrand's compound). The P–C bond distances also agree nicely between [**5**][BAr^F^_4_], 1.795(2) and 1.824(2) Å, and [**5**][B(C_6_F_5_)_4_], 1.795(2) and 1.810(2) Å.

## 3. Conclusions

We demonstrate that thermal treatment of the N-heterocyclic carbene 1,3-bis(2,6-diisopropylphenyl)-imidazol-2-ylidene (IPr) adduct of PBr_3_, (IPr)PBr_3_ (**1**), results in a spontaneous reductive coupling of **1** to afford the phosphorus(ii)–phosphorus(ii) dimer, [P_2_(IPr)_2_Br_3_]Br ([**2**]Br) and bromine (Br_2_). Abstraction of a bromide ion from **2** allows for the isolation of the unprecedented dicationic species [P_2_(IPr)_2_Br_2_]^2+^ (**3**) which was isolated and structurally authenticated as two different [BAr^F^_4_]^–^ salts. The stereochemical configuration of this dication strongly suggests that the bridging bromide ion from **2** is not directly removed, but rather involved in a fluxional process which allows for the removal of one of the terminal bromide ions.

Reduction of **2** with SnBr_2_ or tetrakis(dimethylamino)ethylene (TDAE) affords [P_2_(IPr)_2_Br]^+^ (**4**) and the known radical cation [P_2_(IPr)_2_]˙^+^ (**5**), respectively. These studies show that relatively weak P–Br bonds present compounds **1–4** can be cleaved in a straightforward manner to afford low oxidation state compounds in high yields. Such species have previously only been accessible through use of strong reducing agents such as potassium graphite (KC_8_).
