# Supplementary material for: N-heterocyclic carbene induced reductive coupling of phosphorus tribromide. Isolation of a bromine bridged P–P bond and its subsequent reactivity
Source: Chem Sci. 2016 Jul 20;7(12):6981–7. doi: 10.1039/c6sc02343f (PMC5356258; doi:10.1039/c6sc02343f)
Supplement: Supplementary file 1 [file SC-007-C6SC02343F-s001.pdf]

Supporting Information

N-heterocyclic carbene induced reductive coupling of phosphorus tribromide. Isolation of a bromine bridged P–P bond and its subsequent reactivity.

Jordan B. Waters, Thomas A. Everitt, William K. Myers and Jose M. Goicoechea*

Department of Chemistry, University of Oxford, Chemistry Research Laboratory, 12 Mansfield Road, Oxford, OX1 3TA, U.K.

E-mail: jose.goicoechea@chem.ox.ac.uk

CONTENTS:

1. Experimental details

2. Single crystal X-ray diffraction data

3. NMR spectra

4. ESI-MS spectra

5. UV/Vis spectra

6. EPR spectra

7. GC-MS spectra

8. TGA data

9. Computational data

1. Experimental details

General synthetic methods: All reactions and product manipulations were carried out under an inert atmosphere of argon or dinitrogen using standard Schlenk-line or glovebox techniques (MBraun UNIlab or MBraun LABmaster 130 glovebox maintained at < 0.1 ppm H_2O and < 0.1 ppm O_2). PBr_3 (97%, Sigma-Aldrich) and tetrakis(dimethylamino)ethylene (TDAE; 95%, Fluorochem) were used as received. $SnBr_2$ (99%, Sigma-Aldrich) was recrystallized from hot THF and dried thoroughly under vacuum to remove all coordinated solvent. 1,3-bis(diisopropylphenyl)-imidazol-2-ylidene (IPr) and $Na[B(3,5\text{-}\{CF_3\}_2C_6H_3)_4]$ were prepared according to previously reported literature procedures.[1,2] Hexane (hex; HPLC grade, >97%, Sigma-Aldrich) and dichloromethane (DCM; HPLC grade, ≥99.8%, Sigma-Aldrich) were purified using an MBraun SPS-800 solvent system. Diethyl ether (Et_2O; puriss. p.a., ACS reagent grade, ≥99.8%, Sigma-Aldrich) and tetrahydrofuran (THF; HPLC grade, ≥99.9%, Sigma-Aldrich) were dried and distilled from a sodium metal/benzophenone mixture. Difluorobenzene (DFB; 98%, Fluorochem), fluorobenzene (C_6H_5F; 99%, Alfa Aesar), CD_2Cl_2 (99.9%, Fluorochem) and d_8-THF (99.5%, Fluorochem) were dried and distilled from CaH_2. All dry solvents were stored under argon in gas-tight ampoules over 3 Å molecular sieves.

*Synthesis of (IPr)PBr$_3$ (**1**).* To a solution of IPr (519 mg, 1.338 mmol) in diethyl ether (25 mL) was added a diethyl ether (25 mL) solution of PBr_3 (362 mg, 1.338 mmol) while stirring at ambient temperature affording a yellow solution and a fine white precipitate. The mixture was stirred for one hour before the mixture was filtered and the solution concentrated to 10 mL. The concentrated solution was cooled at –35 °C overnight to afford yellow crystals which were subsequently filtered and dried thoroughly under vacuum (460 mg, 52% crystalline yield). Yellow crystals suitable for single crystal X-ray diffraction were grown by

slow diffusion of hexane into a concentrated THF solution of the product. Anal. Calcd. for $C_{27}H_{36}Br_3N_2P$ (659.27): C 49.19%, H 5.50%, N 4.25%. Found: C 49.98%, H 5.79%, N 4.18%. ^{1}H NMR (500.30 MHz, d_8-THF): δ (ppm) 7.98 (s, 2H; N(C*H*)$_2$N), 7.55 (t, $^3J_{H–H}$ = 8 Hz, 2H; *para*-Dipp), 7.38 (d, $^3J_{H–H}$ = 8 Hz, 4H; *meta*-Dipp), 3.30 (sept, $^3J_{H–H}$ = 7 Hz, 4H; C_6H_3{C*H*(CH_3)}$_2$, 1.44 (d, $^3J_{H–H}$ = 7 Hz, 12H; C_6H_3{CH(C*H*$_3$)}$_2$), 1.12 (d, $^3J_{H–H}$ = 7 Hz, 12H; C_6H_3{CH(C*H*$_3$)}$_2$). ^{13}C{^{1}H} NMR (125.80 MHz, d_8-THF): δ (ppm) 149.2 (d, $^1J_{^{13}C–^{31}P}$ = 176 Hz; *C*N$_2$), 148.0 (*ortho*-Dipp), 133.6 (*ipso*-Dipp), 132.7 (*para*-Dipp), 128.6 (d, $^3J_{^{13}C–^{31}P}$ = 4 Hz; N(*C*H)$_2$N), 125.5 (*meta*-Dipp), 30.4 (C_6H_3{*C*H(CH_3)}$_2$), 26.6, 23.9 (C_6H_3{CH(*C*H$_3$)}$_2$). ^{31}P NMR (202.38 MHz, d_8-THF): δ (ppm) 24.8 (s).

*Synthesis of [P$_2$(IPr)$_2$Br$_3$]Br ([**2**]Br).* To a solution of IPr (2.570 g, 7.088 mmol) in THF (50 mL) was added PBr_3 (1.919 g, 7.088 mmol) while stirring at ambient temperature to afford a yellow solution. The solution was then heated without stirring to 65 °C for three days. During this time, a colour change from yellow to red is observed followed by the formation of large deep red crystals which were suitable for single crystal X-ray diffraction. The red solution is then filtered and the crystals washed with warm THF until the washings remain colourless. The crystals were then dried thoroughly under vacuum at 65 °C (2.943 g, 72 % crystalline yield). Anal. Calcd. for $C_{54}H_{72}Br_4N_4P_2$ (1158.73): C 55.97%, H 6.26%, N 4.84%. Found: C 56.79%, H 6.34%, N 4.90%. ESI-MS, positive-ion mode (DCM, 60 °C, 4.5 kV): *m*/*z* 1079.2753 (100%) [P$_2$(IPr)$_2$Br$_3$]$^+$ (Calcd. 1079.2739). ^{1}H NMR (500.30 MHz, CD_2Cl_2): δ (ppm) 7.60 (t, $^3J_{H–H}$ = 8 Hz, 2H; *para*-Dipp), 7.55 (d, $^3J_{H–H}$ = 2 Hz, 2H; N(C*H*)(CH)N), 7.51 (t, $^3J_{H–H}$ = 8 Hz, 2H; *para*-Dipp), 7.43 (d, $^3J_{H–H}$ = 2 Hz, 2H; N(CH)(C*H*)N), 7.41 (dd, $^3J_{H–H}$ = 8 Hz, $^4J_{H–H}$ = 1 Hz, 2H; *meta*-Dipp), 7.36 (dd, $^3J_{H–H}$ = 8 Hz, $^4J_{H–H}$ = 1 Hz, 2H; *meta*-Dipp), 7.27 (dd, $^3J_{H–H}$ = 8 Hz, $^4J_{H–H}$ = 1 Hz, 2H; *meta*-Dipp), 7.06 (dd, $^3J_{H–H}$ = 8 Hz, $^4J_{H–H}$ = 1 Hz, 2H; *meta*-Dipp), 2.81 (sept, $^3J_{H–H}$ = 7 Hz, 2H; C_6H_3{C*H*(CH_3)}$_2$, 2.72 (sept, $^3J_{H–H}$ = 7 Hz,

2H; $C_6H_3\{CH(CH_3)\}_2$, 2.56 (sept, $^3J_{H–H}$ = 7 Hz, 2H; $C_6H_3\{CH(CH_3)\}_2$, 2.55 (sept, $^3J_{H–H}$ = 7 Hz, 2H; $C_6H_3\{CH(CH_3)\}_2$, 1.37 (d, $^3J_{H–H}$ = 7 Hz, 6H; $C_6H_3\{CH(CH_3)\}_2$), 1.33 (d, $^3J_{H–H}$ = 7 Hz, 6H; $C_6H_3\{CH(CH_3)\}_2$), 1.28 (d, $^3J_{H–H}$ = 7 Hz, 6H; $C_6H_3\{CH(CH_3)\}_2$), 1.07 (d, $^3J_{H–H}$ = 7 Hz, 6H; $C_6H_3\{CH(CH_3)\}_2$), 1.05 (d, $^3J_{H–H}$ = 7 Hz, 6H; $C_6H_3\{CH(CH_3)\}_2$), 1.00 (d, $^3J_{H–H}$ = 7 Hz, 6H; $C_6H_3\{CH(CH_3)\}_2$), 0.81 (d, $^3J_{H–H}$ = 7 Hz, 6H; $C_6H_3\{CH(CH_3)\}_2$), 0.51 (d, $^3J_{H–H}$ = 7 Hz, 6H; $C_6H_3\{CH(CH_3)\}_2$). $^{13}C\{^1H\}$ NMR (125.80 MHz, CD_2Cl_2): δ (ppm) 150.2 (m; CN_2), 148.1 (t, $D_{^{13}C–^{31}P}$ = 3 Hz; *ortho*-Dipp), 147.3, 147.2, 146.8 (*ortho*-Dipp), 133.4, 132.9 (*para*-Dipp), 131.4 (t, $D_{^{13}C–^{31}P}$ = 2 Hz; *ipso*-Dipp), 131.2 (*ipso*-Dipp), 128.7 (N(*C*H)(CH)N), 126.7 (N(CH)(*C*H)N), 125.9, 125.6, 125.1, 124.3 (*meta*-Dipp), 30.3, 30.0, 29.7 ($C_6H_3\{CH(CH_3)\}_2$), 28.9 (t, $D_{^{13}C–^{31}P}$ = 2 Hz; ($C_6H_3\{CH(CH_3)\}_2$), 27.1, 26.8, 26.6, 26.3, 23.2, 23.1, 23.0 ($C_6H_3\{CH(CH_3)\}_2$), 21.1 (t, $D_{^{13}C–^{31}P}$ = 4 Hz; $C_6H_3\{CH(CH_3)\}_2$). ^{31}P NMR (202.38 MHz, CD_2Cl_2): δ (ppm) –27.3 (s). UV/Vis (C_6H_5F): λ_{max} 461 nm.

Synthesis of $[P_2(IPr)_2Br_3][B(3,5\text{-}\{CF_3\}_2C_6H_3)_4)_2]$ *([**2**][BAr$^F_4$]).* THF (25 mL) was added to a mixture of $[P_2(IPr)_2Br_3]Br$ (329 mg, 0.284 mmol) and $Na[BAr^F_4]$ (252 mg, 0.284 mmol) and the resulting suspension stirred overnight to afford an orange solution and colourless precipitate. The solution was filtered and the solvent removed *in vacuo* to afford an orange solid (347 mg, 63% yield). Orange crystals suitable for single crystal X-ray diffraction were grown by slow diffusion of hexane into a THF solution. Anal. Calcd. for $C_{86}H_{84}BBr_3F_{24}N_4P_2$ (1942.23): C 53.18%, H 4.36%, N 2.88%. Found: C 53.25%, H 4.40%, N 2.98%. ^1H NMR (500.30 MHz, d_8-THF): δ (ppm) 8.05 (s, 2H; N(C*H*)(CH)N), 7.97 (s, 2H; N(CH)(C*H*)N), 7.79 (s, 8H; *ortho*- BAr^F_4), 7.62 (t, $^3J_{H–H}$ = 8 Hz, 2H; *para*-Dipp), 7.57 (s, 4H; *para*- BAr^F_4), 7.53 (t, $^3J_{H–H}$ = 8 Hz, 2H; *para*-Dipp), 7.48 (d, $^3J_{H–H}$ = 8 Hz, 2H; *meta*-Dipp), 7.43 (d, $^3J_{H–H}$ = 8 Hz, 2H; *meta*-Dipp), 7.34 (d, $^3J_{H–H}$ = 8 Hz, 2H; *meta*-Dipp), 7.12 (d, $^3J_{H–H}$ = 8 Hz, 2H; *meta*-Dipp), 2.92 (sept, $^3J_{H–H}$ = 7 Hz, 2H; $C_6H_3\{CH(CH_3)\}_2$, 2.80 (sept,

$^3J_{H–H}$ = 7 Hz, 2H; $C_6H_3\{CH(CH_3)\}_2$, 2.67 (sept, $^3J_{H–H}$ = 7 Hz, 2H; $C_6H_3\{CH(CH_3)\}_2$, 2.60 (sept, $^3J_{H–H}$ = 7 Hz, 2H; $C_6H_3\{CH(CH_3)\}_2$, 1.41 (d, $^3J_{H–H}$ = 7 Hz, 6H; $C_6H_3\{CH(CH_3)\}_2$), 1.36 (d, $^3J_{H–H}$ = 7 Hz, 6H; $C_6H_3\{CH(CH_3)\}_2$), 1.32 (d, $^3J_{H–H}$ = 7 Hz, 6H; $C_6H_3\{CH(CH_3)\}_2$), 1.09 (d, $^3J_{H–H}$ = 7 Hz, 6H; $C_6H_3\{CH(CH_3)\}_2$), 1.06 (d, $^3J_{H–H}$ = 7 Hz, 6H; $C_6H_3\{CH(CH_3)\}_2$), 1.01 (d, $^3J_{H–H}$ = 7 Hz, 6H; $C_6H_3\{CH(CH_3)\}_2$), 0.84 (d, $^3J_{H–H}$ = 7 Hz, 6H; $C_6H_3\{CH(CH_3)\}_2$), 0.57 (d, $^3J_{H–H}$ = 7 Hz, 6H; $C_6H_3\{CH(CH_3)\}_2$). $^{13}C\{^1H\}$ NMR (125.80 MHz, CD_2Cl_2): δ (ppm) 163.0 (m, $^1J_{^{13}C–^{11}B}$ = 50 Hz, $^1J_{^{13}C–^{10}B}$ = 17 Hz; *ipso*- BAr^F_4), 150.3 (m, CN_2), 148.8 (t, $D_{^{13}C–^{31}P}$ = 2 Hz; *ortho*-Dipp), 148.1, 148.0, 147.5 (*ortho*-Dipp), 135.8 (*ortho*- BAr^F_4), 134.0, 133.4 (*para*-Dipp), 132.7 (t, $D_{^{13}C–^{31}P}$ = 2 Hz; *ipso*-Dipp), 132.4 (*ipso*-Dipp), 130.3 (N(CH)(CH)N), 130.2 (qq, $^2J_{^{13}C–^{19}F}$ = 31 Hz, $^4J_{^{13}C–^{19}F}$ = 2 Hz; *meta*- BAr^F_4), 128.2 (N(CH)(CH)N), 126.7, 126.4 (*meta*-Dipp), 125.7 (q, $^1J_{^{13}C–^{19}F}$ = 271 Hz; CF_3-BAr^F_4), 125.7, 124.9 (*meta*-Dipp), 118.4 (sept, $^3J_{^{13}C–^{19}F}$ = 4 Hz; *para*- BAr^F_4), 31.0, 30.7, 30.5 ($C_6H_3\{CH(CH_3)\}_2$), 29.7 (t, $D_{^{13}C–^{31}P}$ = 2 Hz; ($C_6H_3\{CH(CH_3)\}_2$), 26.8, 26.4, 26.3, 26.2, 23.7, 23.5, 23.4 ($C_6H_3\{CH(CH_3)\}_2$), 21.8 (t, $D_{^{13}C–^{31}P}$ = 4 Hz; $C_6H_3\{CH(CH_3)\}_2$). ^{31}P NMR (202.38 MHz, d_8-THF): δ (ppm) –26.8 (s). ^{11}B NMR (128.39 MHz, DFB): δ (ppm) –6.2 (s). ^{19}F NMR (376.54 MHz, DFB): δ (ppm) –63.1 (s).

Synthesis of $[P_2(IPr)_2Br_2][B(3,5\text{-}\{CF_3\}_2C_6H_3)_4)_2]_2$ *([**3**]*$[BAr^F_4]_2$*).* DFB (5 mL) was added to a mixture of $[P_2(IPr)_2Br_3][BAr^F_4]$ (105 mg, 0.0541 mmol) and $Na[BAr^F_4]$ (48 mg, 0.0541 mmol) while stirring at ambient temperature yielding a cloudy yellow solution. The solution was then filtered and the solvent removed *in vacuo* to afford a pale yellow crystalline solid which was then washed with DCM (5 mL) and the solid dried thoroughly under vacuum (145 mg, 99% yield). Crystals suitable for single crystal X-ray diffraction were grown from a saturated DCM solution at room temperature or by slow diffusion of hexane into a DFB solution. Anal. Calcd. for $C_{118}H_{96}B_2Br_2F_{48}N_4P_2$ (2725.75): C 52.00%, H 3.55%, N 2.05%.

Found: C 52.28%, H 3.42%, N 1.86%. ESI-MS, positive ion mode (DFB, 60 °C, 4.5 kV): *m/z* 1861.5209 (3%) $[P_2(IPr)_2Br_2(BAr^F{}_4)]^+$ (Calcd. 1861.4248), 1079.2500 (76%) $[P_2(IPr)_2Br_3]^+$ (Calcd. 1079.2739), 1015.3296 (100%) $[P_2(IPr)_2(OH)]^+$ (Calcd. 1015.3608), 499.1745 (15%) $[P_2(IPr)_2Br_2]^{2+}$ (Calcd. 499.1787), 468.2322 (5%) $[P_2(IPr)_2Br(OH)]^{2+}$ (Calcd. 468.2208). ^{1}H NMR (500.30 MHz, DFB): δ (ppm) 8.31 (s, 16H; *ortho*-$BAr^F{}_4$), 8.06 (s, 4H; $N(CH)_2N$), 7.79 (t, $^3J_{H–H}$ = 8 Hz, 4H; *para*-Dipp), 7.69 (s, 8H; *para*-$BAr^F{}_4$), 7.46 (d, $^3J_{H–H}$ = 8 Hz, 4H; *meta*-Dipp), 7.38 (d, $^3J_{H–H}$ = 8 Hz, 4H; *meta*-Dipp), 2.40 (overlapping sept, $^3J_{H–H}$ = 7 Hz, 8H; $C_6H_3\{CH(CH_3)\}_2$, 1.34 (d, $^3J_{H–H}$ = 7 Hz, 12H; $C_6H_3\{CH(CH_3)\}_2$), 1.27 (d, $^3J_{H–H}$ = 7 Hz, 12H; $C_6H_3\{CH(CH_3)\}_2$), 1.19 (d, $^3J_{H–H}$ = 7 Hz, 24H; $C_6H_3\{CH(CH_3)\}_2$). ^{13}C{^{1}H} NMR (125.80 MHz, DFB): δ (ppm) 163.0 (m, $^1J_{^{13}C–^{11}B}$ = 50 Hz, $^1J_{^{13}C–^{10}B}$ = 17 Hz; *ipso*-$BAr^F{}_4$), 146.3, 146.1 (*ortho*-Dipp), 138.5 (m, CN_2), 135.6; *ortho*-$BAr^F{}_4$), 134.7 (N(*C*H)(CH)N), 132.2 (*para*-Dipp), 130.2 (s, *ipso*-Dipp and qq, $^2J_{^{13}C–^{19}F}$ = 31 Hz, $^4J_{^{13}C–^{19}F}$ = 2 Hz; *meta*-$BAr^F{}_4$), 126.1, 125.8 (*meta*-Dipp), 125.4 (q, $^1J_{^{13}C–^{19}F}$ = 271 Hz; *C*F_3-$BAr^F{}_4$), 118.1 (sept, - $^3J_{^{13}C–^{19}F}$ = 4 Hz; *para*-$BAr^F{}_4$), 30.5, 30.3 ($C_6H_3\{CH(CH_3)\}_2$), 26.2, 25.9, 21.9 (×2) ($C_6H_3\{CH(CH_3)\}_2$). ^{31}P NMR (202.38 MHz, DFB): δ (ppm) –1.8 (s). ^{11}B NMR (128.39 MHz, DFB): δ (ppm) –6.2 (s). ^{19}F NMR (376.54 MHz, DFB): δ (ppm) –63.1 (s). UV/Vis (DFB): λ_{max} 382 nm.

Synthesis of $[P_2(IPr)_2Br][SnBr_5(THF)]$ *([**4**]*$[SnBr_5(THF)]$*).* THF (10 mL) was added to a mixture of $[IPr_2P_2Br_3]Br$ (234 mg, 0.202 mmol) and $SnBr_2$ (56 mg, 0.202 mmol) while stirring at ambient temperature to afford a deep red solution. The mixture was stirred for five minutes before the solvent was removed *in vacuo* to afford a deep red solid (290 mg, 95% yield). Crystals of $[IPr_2P_2Br]_2[SnBr_6]$ suitable for single crystal X-ray diffraction were grown from the reaction mixture on addition of half an equivalent of IPr (which reacts with the $[SnBr_5(THF)]^-$ anion to afford $SnBr_4(IPr)$ and $[SnBr_6]^{2-}$). ESI-MS, positive ion mode

(DCM, 60 °C, 4.5 kV): *m/z* 919.5514 (100%) $[P_2(IPr)_2Br]^+$ (Calcd 919.4394). ^{1}H NMR (500.30 MHz, d_8-THF, 338 K): δ (ppm) 7.72 (s, 4H; N(C*H*)$_2$N), 7.50 (t, $^3J_{H–H}$ = 8 Hz, 4H; *para*-Dipp), 7.25 (d, $^3J_{H–H}$ = 8 Hz, 8H; *meta*-Dipp), 2.50 (sept, 8H; $^3J_{H–H}$ = 7 Hz; C_6H_3{C*H*(CH_3)}$_2$), 1.07 (d, $^3J_{H–H}$ = 7 Hz, 48H; C_6H_3{CH(C*H*$_3$)}$_2$). ^{1}H NMR (500.30 MHz, d_8-THF, 208 K): δ (ppm) 8.24 (s, 1H; N(C*H*)$_2$N), 8.20 (s, 1H; N(C*H*)$_2$N), 7.80 (s, 2H; N(C*H*)$_2$N), 7.64 (br t, $^3J_{H–H}$ = 8 Hz, 1H; *para*-Dipp), 7.46–7.58 (m, 5H; *meta*- and *para*-Dipp); 7.40 (br d, $^3J_{H–H}$ = 8 Hz, 1H; *meta*-Dipp), 7.34 (d, $^3J_{H–H}$ = 8 Hz, 2H; *meta*-Dipp), 7.26 (d, $^3J_{H–H}$ = 8 Hz, 2H; *meta*-Dipp), 7.00 (br d, $^3J_{H–H}$ = 8 Hz, 1H; *meta*-Dipp), 3.14 (br sept, $^3J_{H–H}$ = 7 Hz, 1H; C_6H_3{C*H*(CH_3)}$_2$), 2.60 (br sept, $^3J_{H–H}$ = 7 Hz, 1H; C_6H_3{C*H*(CH_3)}$_2$), 2.51 (sept, $^3J_{H–H}$ = 7 Hz, 2H; C_6H_3{C*H*(CH_3)}$_2$), 2.30 (sept, $^3J_{H–H}$ = 7 Hz, 2H; C_6H_3{C*H*(CH_3)}$_2$), 2.15 (br sept, $^3J_{H–H}$ = 7 Hz, 1H; C_6H_3{C*H*(CH_3)}$_2$), 2.96 (br sept, $^3J_{H–H}$ = 7 Hz, 1H; C_6H_3{C*H*(CH_3)}$_2$), 1.29 (d, $^3J_{H–H}$ = 7 Hz, 3H; C_6H_3{CH(C*H*$_3$)}$_2$), 1.22 (d, $^3J_{H–H}$ = 7 Hz, 12H; C_6H_3{CH(C*H*$_3$)}$_2$), 1.17 (d, $^3J_{H–H}$ = 7 Hz, 3H; C_6H_3{CH(C*H*$_3$)}$_2$), 1.12 (d, $^3J_{H–H}$ = 7 Hz, 6H; C_6H_3{CH(C*H*$_3$)}$_2$), 1.08 (d, $^3J_{H–H}$ = 7 Hz, 3H; C_6H_3{CH(C*H*$_3$)}$_2$), 1.01 (d, $^3J_{H–H}$ = 7 Hz, 9H; C_6H_3{CH(C*H*$_3$)}$_2$), 0.95 (d, $^3J_{H–H}$ = 7 Hz, 3H; C_6H_3{CH(C*H*$_3$)}$_2$), 0.79 (d, $^3J_{H–H}$ = 7 Hz, 6H; C_6H_3{CH(C*H*$_3$)}$_2$), 0.57 (d, $^3J_{H–H}$ = 7 Hz, 3H; C_6H_3{CH(C*H*$_3$)}$_2$). ^{13}C{^{1}H} NMR (125.80 MHz, d_8-THF, 338 K): δ (ppm) 157.8 (v br s, *C*N$_2$), 147.0 (*ortho*-Dipp), 133.4 (*ipso*-Dipp), 132.9 (*para*-Dipp), 128.3 (N(*C*H)$_2$N), 125.9 (*meta*-Dipp), 30.2 (C_6H_3{*C*H(CH_3)}$_2$), 25.9, 23.5 (C_6H_3{CH(*C*H$_3$)}$_2$). ^{13}C{^{1}H} NMR (125.80 MHz, d_8-THF, 208 K): δ (ppm) 164.2 (dd, $^1J_{^{13}C–^{31}P}$ = 118 Hz, $^2J_{^{13}C–^{31}P}$ = 32 Hz; *C*N$_2$), 150.6 (dd, $^1J_{^{13}C–^{31}P}$ = 85 Hz, $^2J_{^{13}C–^{31}P}$ = 26 Hz; *C*N$_2$), 147.2 (×2), 147.1, 146.7, 146.6, 145.9 (×2), 145.4 (*ortho*-Dipp), 133.7, 133.5 (×2), 133.0 (*ipso*-Dipp), 132.7, 132.6 (×2), 132.4 (*para*-Dipp), 130.5, 127.3, 127.2 (×2) (N(*C*H)$_2$N), 126.6, 126.3 (×2), 126.1, 126.0, 125.5, 125.2 (×2) (*meta*-Dipp), 30.3, 30.2 (×2), 29.9 (C_6H_3{*C*H(CH_3)}$_2$), 24.2, 23.9, 23.6 (×2), 23.3, 23.1, 22.0 (×2) (C_6H_3{CH(*C*H$_3$)}$_2$). No observable resonances in the ^{31}P NMR at

338 K. ^{31}P NMR (202.38 MHz, d_8-THF, 208 K): δ (ppm) 145.4 (d, $^1J_{^{31}P-^{31}P}$ = 391 Hz; (IPr)*P*PBr(IPr)); –7.6 (d, $^1J_{^{31}P-^{31}P}$ = 391 Hz; (IPr)P*P*Br(IPr)). ^{119}Sn{^{1}H} NMR (186.43 MHz, d_8-THF, 298 K): δ (ppm) –1657.7 (s).

*Synthesis of [P_2(IPr)$_2$][BAr$^F{}_4$] ([**5**][BAr$^F{}_4$]).* To a solution of [P_2(IPr)$_2$$Br_3$][BAr$^F{}_4$] (44 mg, 0.023 mmol) in THF (500 μL) was added tetrakis(dimethylamino)ethylene (7 mg, 0.034 mmol) via microsyringe to instantly afford a deep purple solution. After 30 minutes, the solvent was removed *in vacuo* and the product extracted with diethyl ether (5 mL). After filtration, the solvent was removed *in vacuo* to afford a black solid (20 mg, 52% yield). Crystals suitable for single crystal X-ray diffraction were grown by slow diffusion of hexane into a THF solution of the product. Anal. Calcd. for $C_{86}H_{84}BF_{24}N_4P_2$ (1700.32): C 60.68%, H 4.97%, N 3.29%. Found: C 58.75%, H 5.11%, N 3.22%. ESI-MS, positive ion mode (DCM, 60 °C, 4.5 kV): *m/z* 838.5018 (100%) [P_2(IPr)$_2$]$^+$ (Calcd. 838.5227). No observable resonances in the ^{31}P NMR spectrum. UV/Vis (C_6H_5F): λ_{max} 464 nm; 588 nm.

Characterisation techniques: Single crystal X-ray diffraction data were collected using an Oxford Diffraction Supernova dual-source diffractometer equipped with a 135 mm Atlas CCD area detector. Crystals were selected under Paratone-N oil, mounted on micromount loops and quench-cooled using an Oxford Cryosystems open flow N_2 cooling device.[3] Data were collected at 150 K using mirror monochromated Cu Kα radiation (λ = 1.5418 Å; Oxford Diffraction Supernova). Data were processed using the CrysAlisPro package, including unit cell parameter refinement and inter-frame scaling (which was carried out using SCALE3 ABSPACK within CrysAlisPro).[4] Structures were subsequently solved using direct methods or using the charge flipping algorithm as implemented in the program SUPERFLIP,[5] and refined on F^2 using the SHELXL 2013-4 package.[6]

NMR samples were prepared inside a glovebox under nitrogen in NMR tubes equipped with a gas-tight valve. ^{1}H, ^{11}B, $^{13}C\{^{1}H\}$, ^{19}F, ^{31}P and $^{119}Sn\{^{1}H\}$ NMR spectra were acquired on a Bruker AVII or AVIII NMR spectrometer at 298 K unless otherwise stated. ^{1}H and $^{13}C\{^{1}H\}$ spectra are reported relative to tetramethylsilane (TMS) and were referenced to the most downfield residual solvent resonance (d_8-THF: δ_H 3.58 ppm, δ_C 67.6 ppm; CD_2Cl_2: δ_H 5.32 ppm, δ_C 53.8 ppm; DFB: δ_H 6.95–7.11 ppm, δ_C 151.2 ppm). ^{11}B, ^{19}F, ^{31}P and $^{119}Sn\{^{1}H\}$ NMR spectra were externally referenced to $Et_2O.BF_3$, $CFCl_3$, an 85% solution of H_3PO_4 in H_2O and $SnMe_4$, respectively.

EPR measurements were performed at the Centre for Advanced Electron Spin Resonance (CAESR) of the Chemistry Department of the University of Oxford. The X-band spectrometer was a Bruker-Biospin EMXplus with a PremiumX microwave bridge, and a Bruker BioSpin SHQE-W resonator.

Positive ion mode electrospray mass spectra were recorded on a Bruker MicrOTOF mass spectrometer. The samples (10–20 μM) were prepared inside a glovebox under argon and the sample injected through a standard PEEK tubing feedthrough directly to the mass analyser at 10 μL min^{-1}.[7]

UV/Vis spectra were recorded on a Lambda 750 spectrophotometer. Samples were prepared inside a glovebox under nitrogen in a 1 mm wide silica cuvette equipped with a J. Young valve.

Elemental analyses were performed by Elemental Microanalysis Ltd, Devon. 10–15 mg samples were sent in sealed, evacuated Pyrex ampoules.

2. Single crystal X-ray diffraction data

Table S1. Selected X-ray data collection and refinement parameters for **1**, [**2**]Br·3THF and [**2**][BAr^F_4].

	**1**	[**2**]Br·3THF	[**2**][BAr^F_4]
Formula	$C_{27}H_{36}Br_3N_2P$	$C_{66}H_{96}Br_4N_4O_3P_2$	$C_{86}H_{84}BBr_3F_{24}N_4P_2$
CCDC depository number	1480947	1480948	1480949
Fw [g mol^{-1}]	659.28	1375.04	1942.05
crystal system	monoclinic	triclinic	triclinic
space group	$P2_1/c$	P–1	P–1
a (Å)	10.2830(1)	12.7442(6)	12.8207(1)
b (Å)	18.3022(1)	15.5789(7)	23.5342(3)
c (Å)	15.6495(1)	18.8569(6)	30.8558(4)
α (°)		76.130(3)	90.475(1)
β (°)	108.079(1)	81.932(3)	99.027(1)
γ (°)		68.427(4)	105.727(1)
V ($Å^3$)	2799.85(4)	3374.4(3)	8837.8(2)
Z	4	2	4
radiation, λ (Å)	1.54178, Cu Kα	1.54178, Cu Kα	1.54178, Cu Kα
T (K)	150(2)	150(2)	150(2)
ρ_{calc} (g cm^{-3})	1.564	1.353	1.460
μ (mm^{-1})	6.011	3.708	2.880
reflections collected	31191	35659	127655
independent reflections	5822	13975	36533
parameters	326	728	2283
R(int)	0.0198	0.0288	0.0257
R1/wR2,[a] I ≥ 2σI (%)	2.36/5.77	4.47/13.18	4.27/11.63
R1/wR2,[a] all data (%)	2.42/5.80	4.69/13.39	4.73/12.09
GOF	1.138	1.027	1.025

[a] $R1 = [\Sigma||F_o| - |F_c||]/\Sigma|F_o|$; $wR2 = \{[\Sigma w[(F_o)^2 - (F_c)^2]^2]/[\Sigma w(F_o{}^2)^2]\}^{1/2}$; $w = [\sigma^2(F_o)^2 + (AP)^2 + BP]^{-1}$, where $P = [(F_o)^2 + 2(F_c)^2]/3$ and the A and B values are 0.0242 and 2.67 for **1**, 0.0758 and 8.12 for [**2**]Br·3THF, and 0.0643 and 10.80 for [**2**][BAr^F_4].

Table S2. Selected X-ray data collection and refinement parameters for [**3**][BAr^F_4]$_2$·$2C_6H_4F_2$, [**3**][BAr^F_4]$_2$·$1.5CH_2Cl_2$, [**4**]$_2$[$SnBr_6$]·THF and [**5**][BAr^F_4].

	[**3**][BAr^F_4]$_2$·$2C_6H_4F_2$	[**3**][BAr^F_4]$_2$·$1.5CH_2Cl_2$	[**4**]$_2$[$SnBr_6$]·THF	[**5**][BAr^F_4]
Formula	$C_{130}H_{104}B_2Br_2F_{52}N_4P_2$	$C_{119.5}H_{99}B_2Br_2Cl_3F_{48}N_4P_2$	$C_{112}H_{152}Br_8N_8OP_4Sn$	$C_{86}H_{84}BF_{24}N_4P_2$
CCDC depository number	1480950	1480951	1480952	1480953
Fw [g mol^{-1}]	2953.55	2852.75	2508.26	1702.32
crystal system	triclinic	triclinic	monoclinic	triclinic
space group	*P*–1	*P*–1	*Ia*	*P*–1
a (Å)	12.8045(4)	12.9265(1)	21.5603(2)	12.6812(2)
b (Å)	16.4182(5)	19.5862(2)	26.1169(2)	17.0730(3)
c (Å)	17.5699(4)	25.8965(3)	22.7679(2)	20.7995(4)
α (°)	73.042(2)	91.462(1)		81.712(2)
β (°)	84.650(2)	100.922(1)	111.375(1)	74.258(2)
γ (°)	67.727(3)	103.341(1)		86.832(2)
V (Å^3)	3268.83(18)	6247.54(11)	11938.48(19)	4288.43(14)
Z	1	2	4	2
radiation, λ (Å)	1.54178, Cu Kα	1.54178, Cu Kα	1.54178, Cu Kα	1.54178, Cu Kα
T (K)	150(2)	150(2)	150(2)	150(2)
ρ_{calc} (g cm^{-3})	1.500	1.516	1.396	1.318
μ (mm^{-1})	2.135	2.739	5.708	1.317
reflections collected	32251	107268	72532	84078
independent reflections	11539	25881	18569	17780
parameters	921	1676	1240	1080
R(int)	0.0300	0.0286	0.0228	0.0268
R1/wR2,[a] I ≥ 2σI (%)	5.70/15.97	5.95/15.58	2.98/8.38	5.07/13.18
R1/wR2,[a] all data (%)	6.05/16.36	6.32/15.94	3.03/8.44	5.71/13.78
GOF	1.061	1.054	1.045	1.049

[a] $R1 = [\Sigma||F_o| - |F_c||]/\Sigma|F_o|$; $wR2 = \{[\Sigma w[(F_o)^2 - (F_c)^2]^2]/[\Sigma w(F_o{}^2)^2]\}^{1/2}$; $w = [\sigma^2(F_o)^2 + (AP)^2 + BP]^{-1}$, where $P = [(F_o)^2 + 2(F_c)^2]/3$ and the A and B values are 0.1031 and 2.78 for [**3**][BAr^F_4]$_2$·$2C_6H_4F_2$, 0.0777 and 12.79 for [**3**][BAr^F_4]$_2$·$1.5CH_2Cl_2$, 0.0528 and 17.82 for [**4**]$_2$[$SnBr_6$]·THF, and 0.062 and 3.01 for [**5**][BAr^F_4].

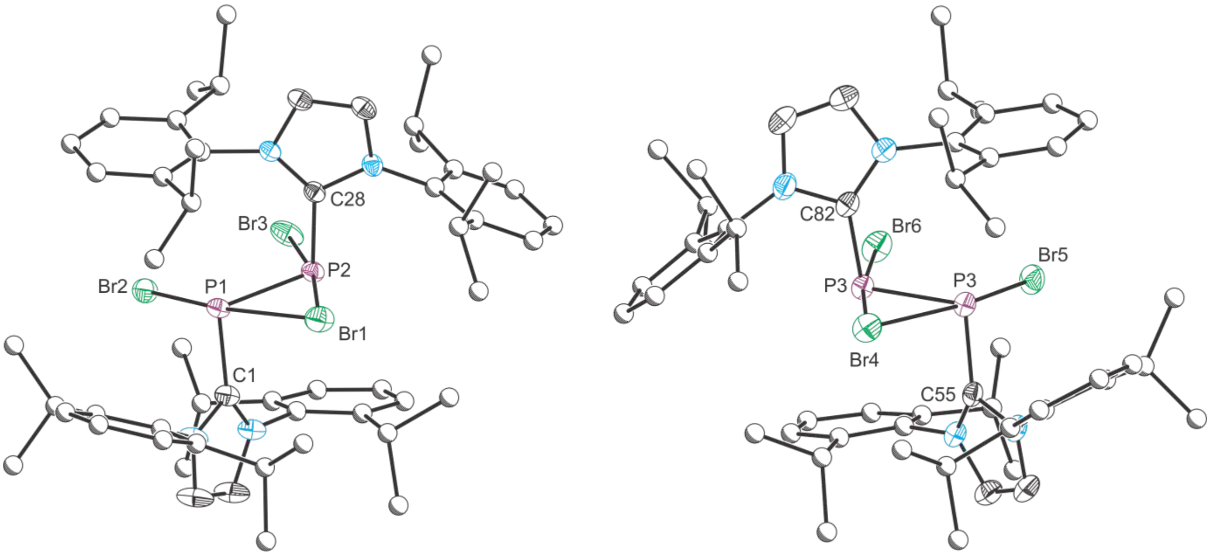

Figure S1. Thermal ellipsoid plots of the two crystallographically unique cationic moieties in [**2**][$BAr^F{}_4$]. Thermal ellipsoids pictured at 50% occupancy level (carbon atoms of Dipp functionalities pictured as spheres of arbitrary radius). All hydrogen atoms removed for clarity.

Table S3. Comparison of selected interatomic distances (Å) for the different salts of **2** and for the optimized computed geometry at the Density Functional level of Theory (**2**$_{\mathbf{DFT}}$).

**Bond**	[**2**]Br·3THF	[**2**][$BAr^F{}_4$]		**2**$_{\mathbf{DFT}}$
**P–P**	2.252(1)	2.264(1)	2.261(2)	2.305
**P–Br**$_{\mathbf{bridge}}$	2.667(1)	2.752(1)	2.723(2)	2.741
	2.810(1)	2.739(2)	2.712(2)	2.738
**P–Br**$_{\mathbf{terminal}}$	2.349(1)	2.295(2)	2.305(2)	2.344
	2.288(1)	2.307(2)	2.300(2)	2.345
**P–C**$_{\mathbf{carbene}}$	1.866(3)	1.867(2)	1.872(2)	1.867
	1.860(3)	1.870(2)	1.861(2)	1.867

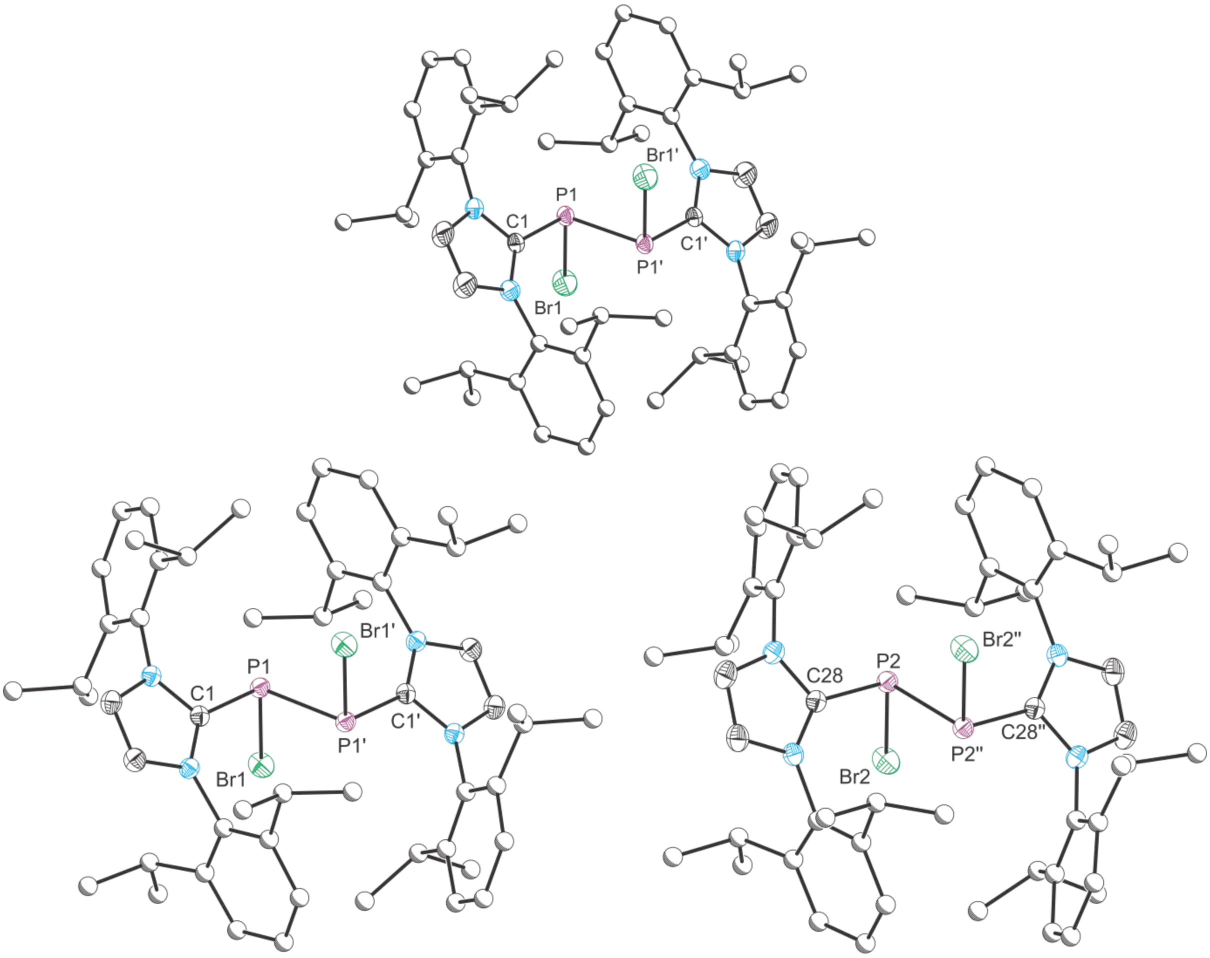

Figure S2. Thermal ellipsoid plots of the crystallographically unique cationic moieties in [**3**][BAr$^F{}_4$]$_2$·2$C_6H_4F_2$ (top) and [**3**][BAr$^F{}_4$]$_2$·1.5CH_2Cl_2 (bottom). Thermal ellipsoids pictured at 50% occupancy level (carbon atoms of Dipp functionalities pictured as spheres of arbitrary radius). All hydrogen atoms removed for clarity.

Table S4. Comparison of selected interatomic distances (Å) for the crystallographically unique cationic moieties in [**3**][BAr$^F{}_4$]$_2$·2$C_6H_4F_2$, [**3**][BAr$^F{}_4$]$_2$·1.5CH_2Cl_2 and for the optimized computed geometry at the Density Functional level of Theory (**3**$_{\mathbf{DFT}}$).

**Bond**	[**3**][BAr$^F{}_4$]$_2$·2$C_6H_4F_2$	[**3**][BAr$^F{}_4$]$_2$·1.5CH_2Cl_2		**3**$_{\mathbf{DFT}}$
**P–P**	2.240(1)	2.232(1)	2.240(1)	2.259
**P–Br**	2.211(1)	2.213(1)	2.219(1)	2.244
**P–C**$_{\mathbf{carbene}}$	1.843(2)	1.850(2)	1.843(3)	1.839

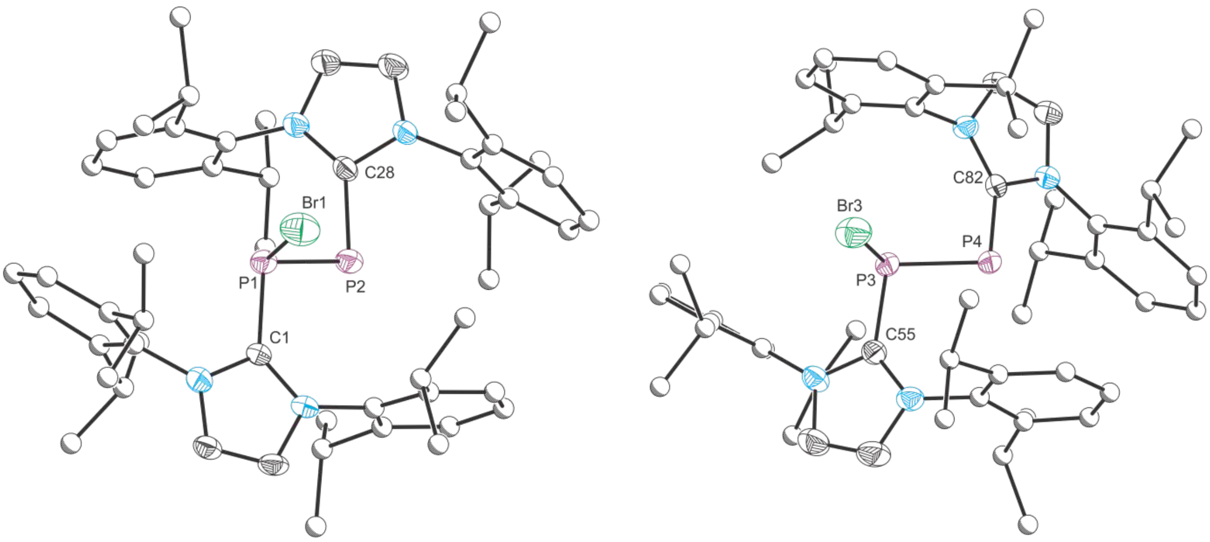

Figure S3. Thermal ellipsoid plots of the two crystallographically unique cationic moieties in [**4**]$_2$[SnBr$_6$]·THF. Thermal ellipsoids pictured at 50% occupancy level (carbon atoms of Dipp functionalities pictured as spheres of arbitrary radius). All hydrogen atoms removed for clarity.

Table S5. Comparison of selected interatomic distances (Å) for the two crystallographically unique cationic moieties in [**4**]$_2$[SnBr$_6$]·THF and for the optimized computed geometry at the Density Functional level of Theory (**4**$_{\mathbf{DFT}}$).

**Bond**	[**4**]$_2$[SnBr$_6$]·THF		**4**$_{\mathbf{DFT}}$
**P–P**	2.096(2)	2.111(2)	2.124
**P–Br**	2.443(1)	2.367(1)	2.425
**P–C**$_{\mathbf{carbene}}$	1.847(5)	1.861(5)	1.841
	1.845(5)	1.821(5)	1.810

3. NMR spectra

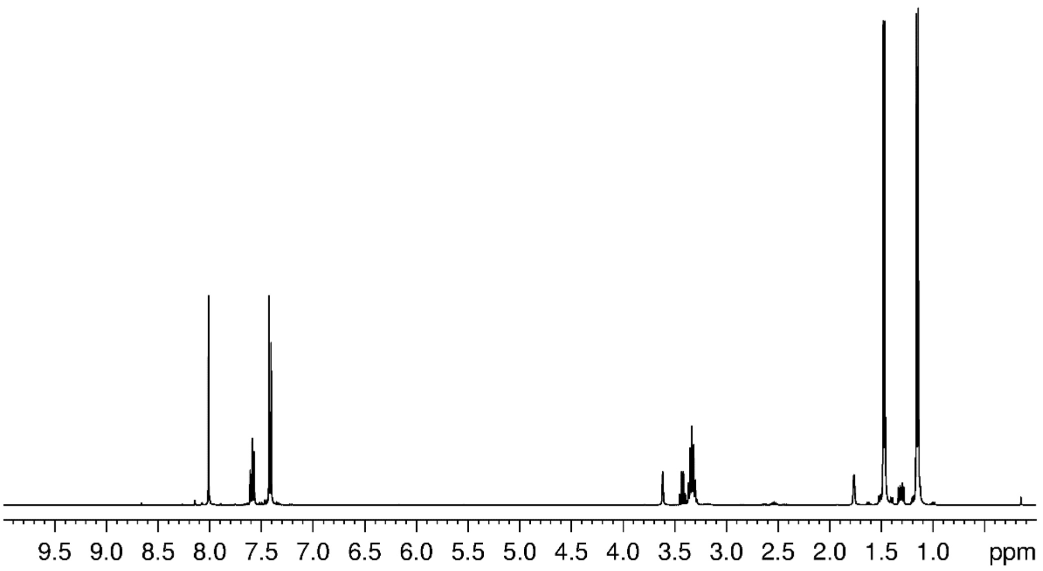

Figure S4. ^{1}H NMR spectrum of **1** in d_8-THF.

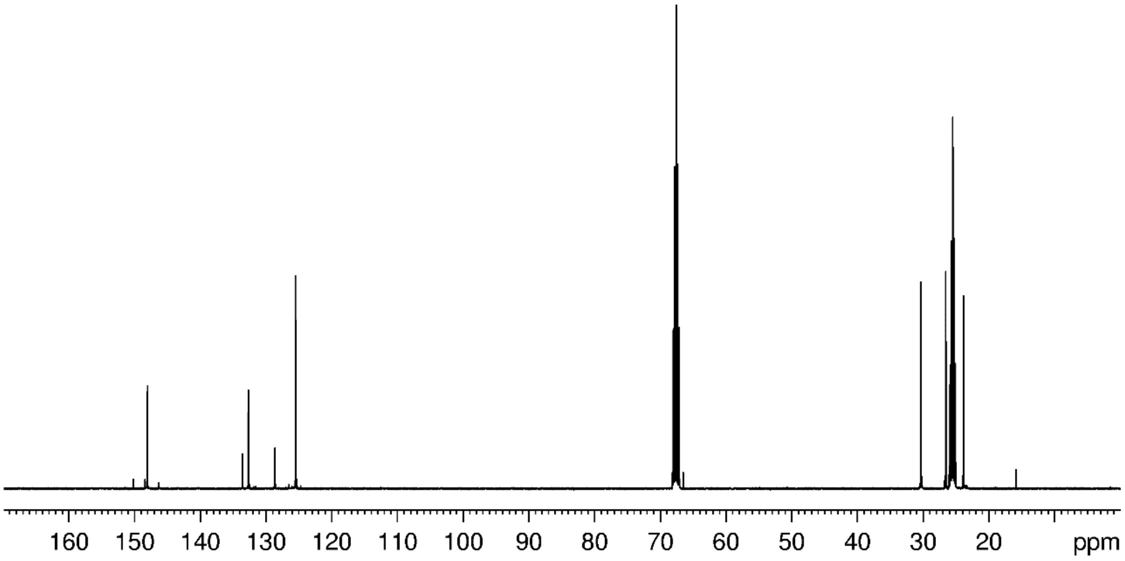

Figure S5. $^{13}C\{^{1}H\}$ NMR spectrum of **1** in d_8-THF.

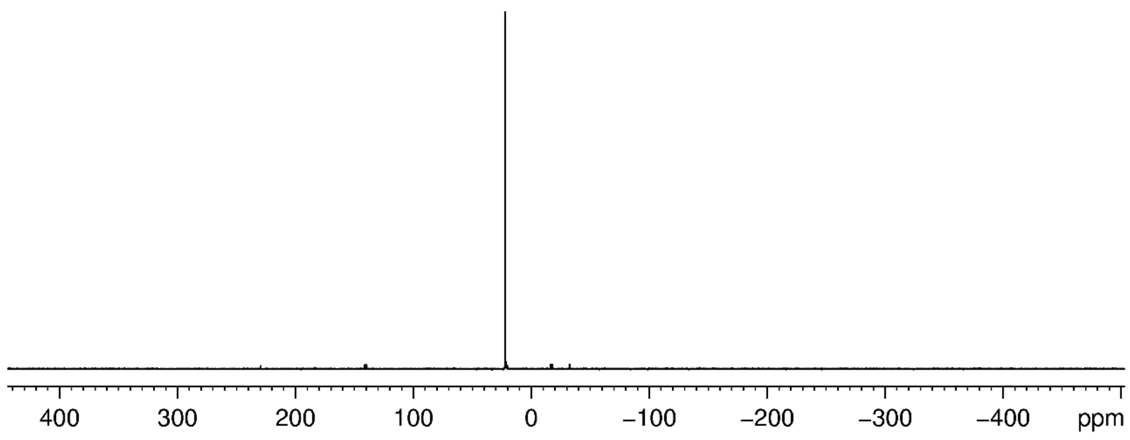

Figure S6. ^{31}P NMR spectrum of **1** in d_8-THF.

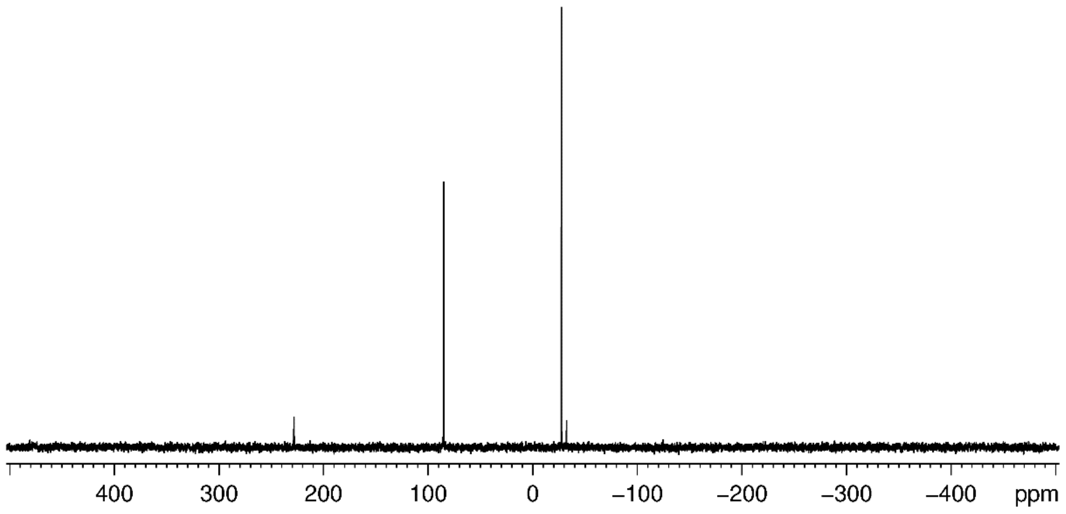

Figure S7. ^{31}P NMR spectrum of **1** after heating at 140 °C under static vacuum for two days followed by subsequent dissolution in dichloromethane. The resonance at 85.2 ppm is attributed to [(IPr)PBr_2]Br while the one at –27.4 ppm corresponds to [P_2(IPr)$_2Br_3$]Br ([**2**]Br).

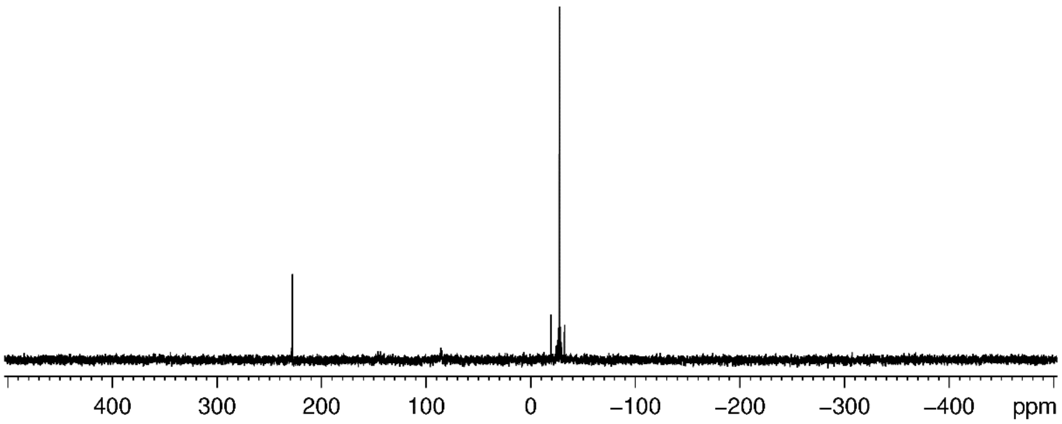

Figure S8. ^{31}P NMR spectrum of **1** after heating at 140 °C under static vacuum for seven days followed by subsequent dissolution in dichloromethane. The resonance at 228.2 ppm is attributed to PBr_3 while the one at –27.4 ppm corresponds to $[P_2(IPr)_2Br_3]Br$ ([**2**]Br).

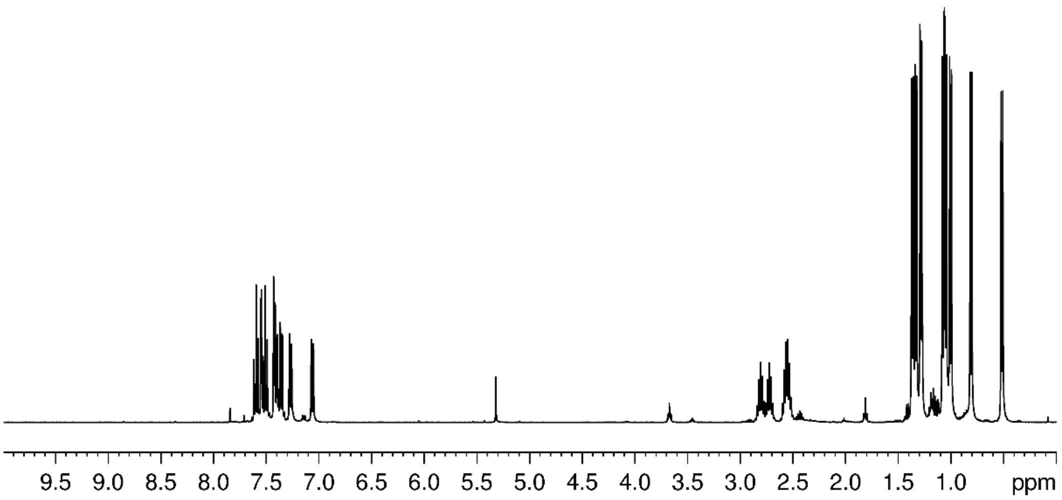

Figure S9. ^{1}H NMR spectrum of [**2**]Br in CD_2Cl_2.

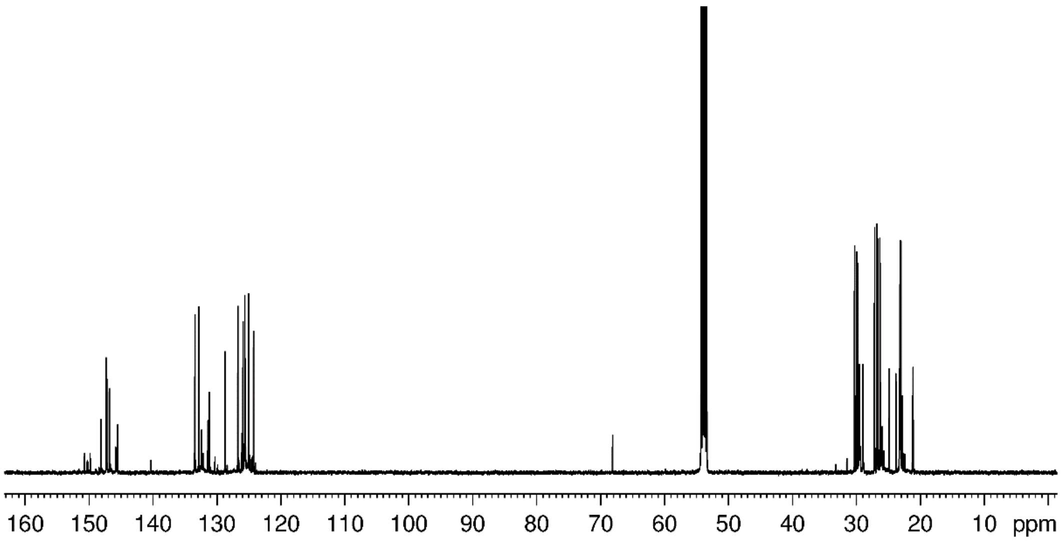

Figure S10. $^{13}C\{^{1}H\}$ NMR spectrum of [**2**]Br in CD_2Cl_2.

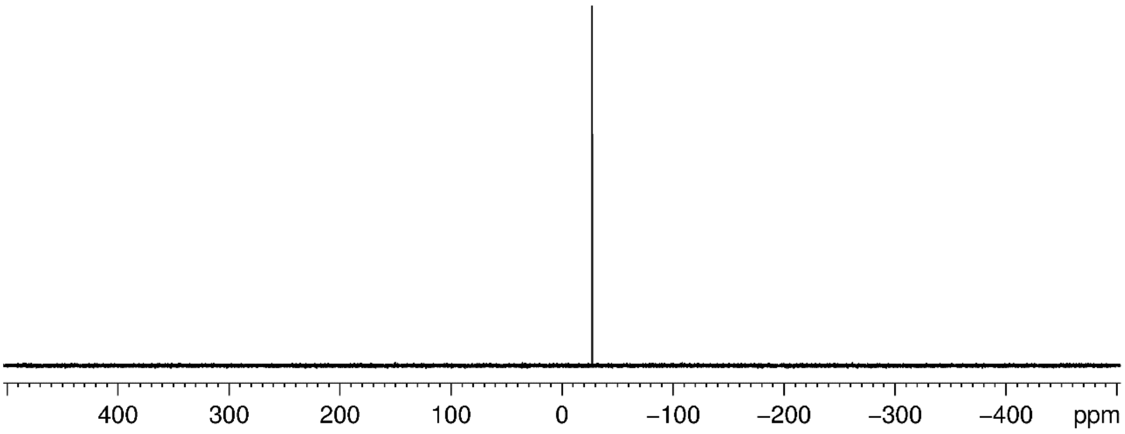

Figure S11. ^{31}P NMR spectrum of [**2**]Br in CD_2Cl_2.

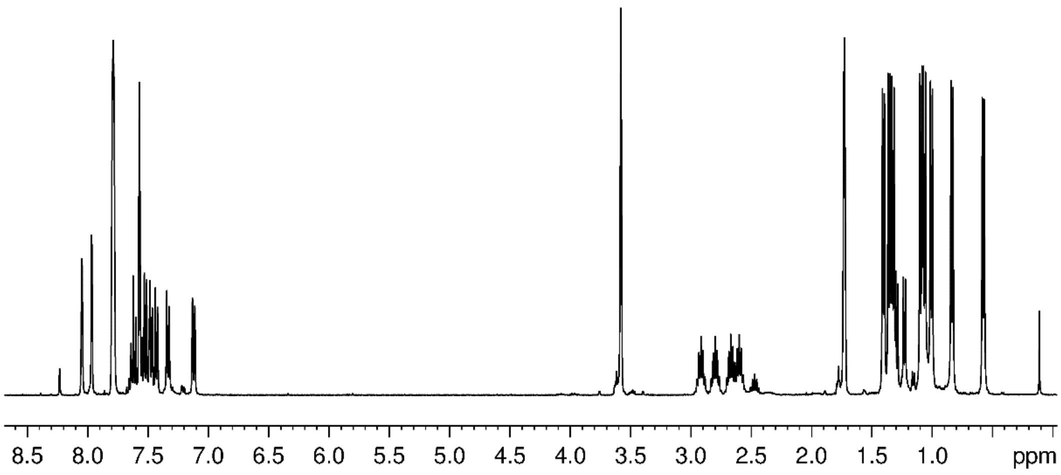

Figure S12. ^{1}H NMR spectrum of [**2**][BAr$^{F}{}_{4}$] in d_8-THF.

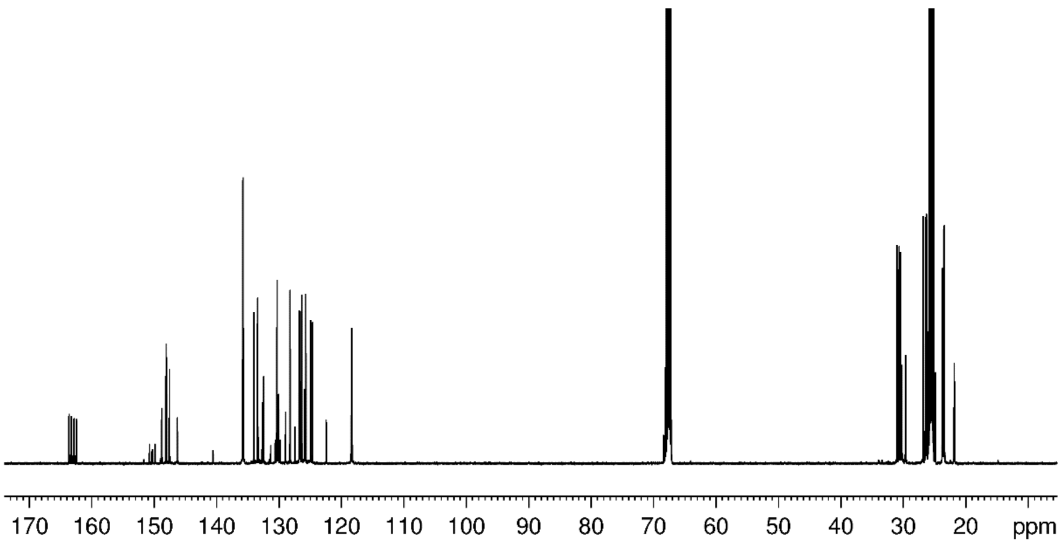

Figure S13. $^{13}C\{^{1}H\}$ NMR spectrum of [**2**][BAr$^{F}{}_{4}$] in d_8-THF.

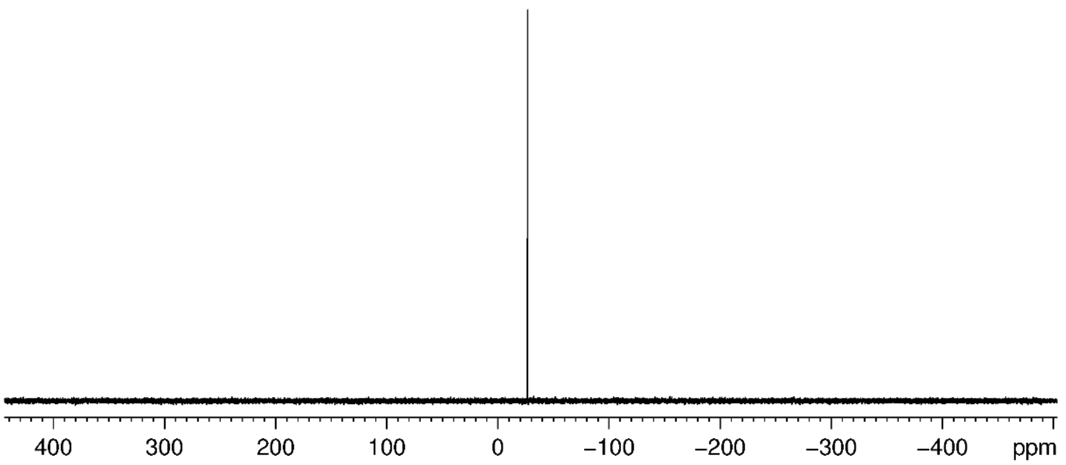

Figure S14. ^{31}P NMR spectrum of [**2**][BAr^F_4] in d_8-THF.

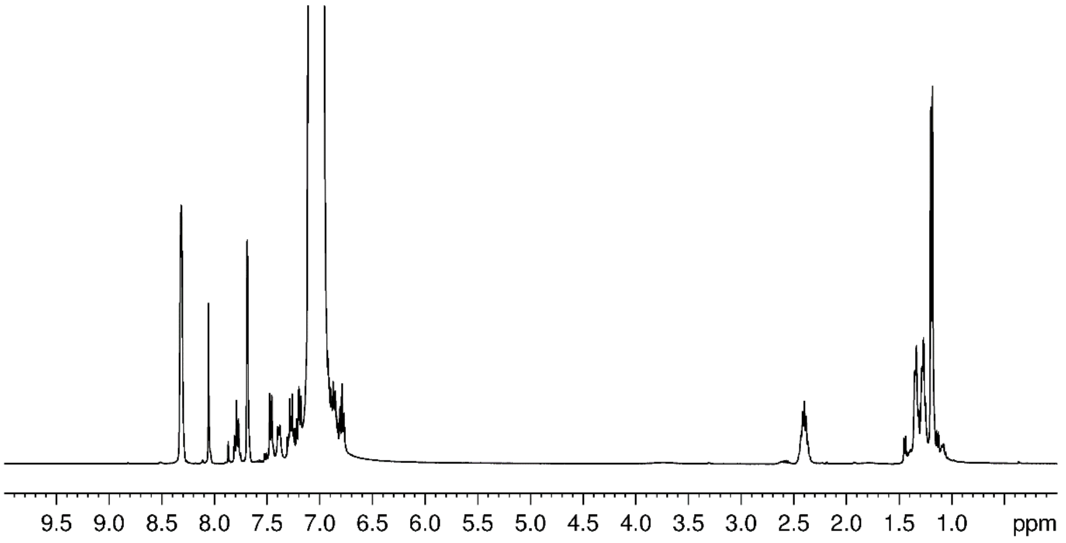

Figure S15. ^{1}H NMR spectrum of [**3**][BAr^F_4]$_2$ in DFB.

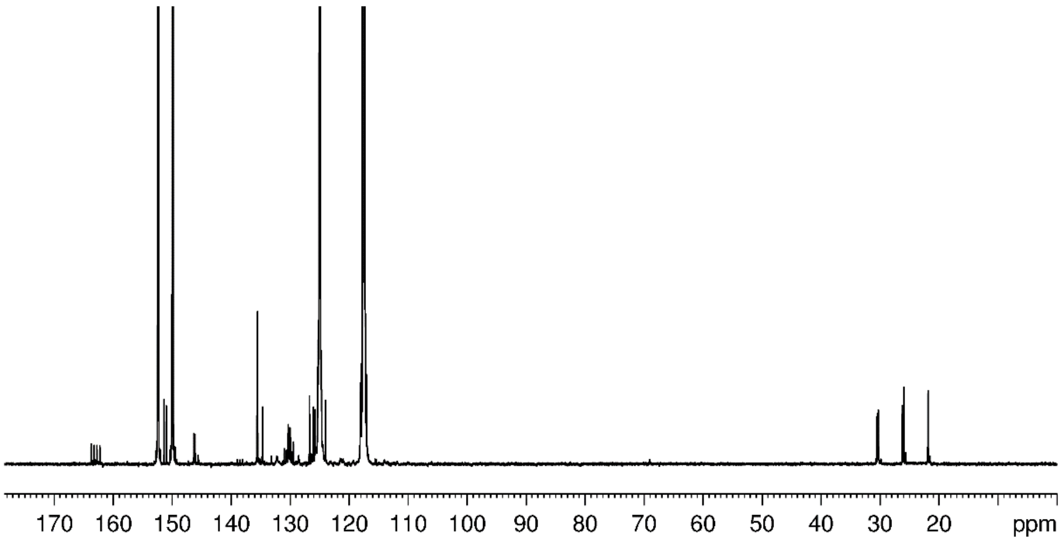

Figure S16. $^{13}C\{^{1}H\}$ NMR spectrum of [**3**][BAr^{F}_{4}]$_2$ in DFB.

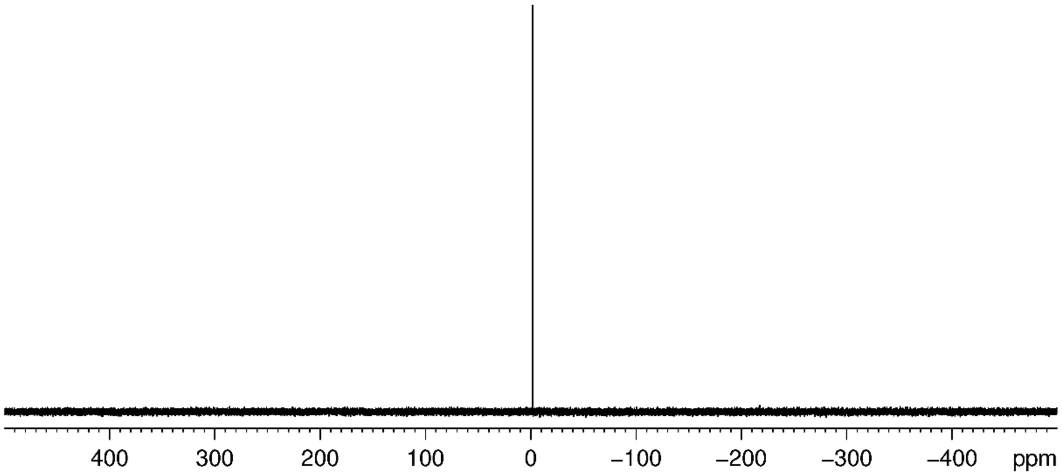

Figure S17. ^{31}P NMR spectrum of [**3**][BAr^{F}_{4}]$_2$ in DFB.

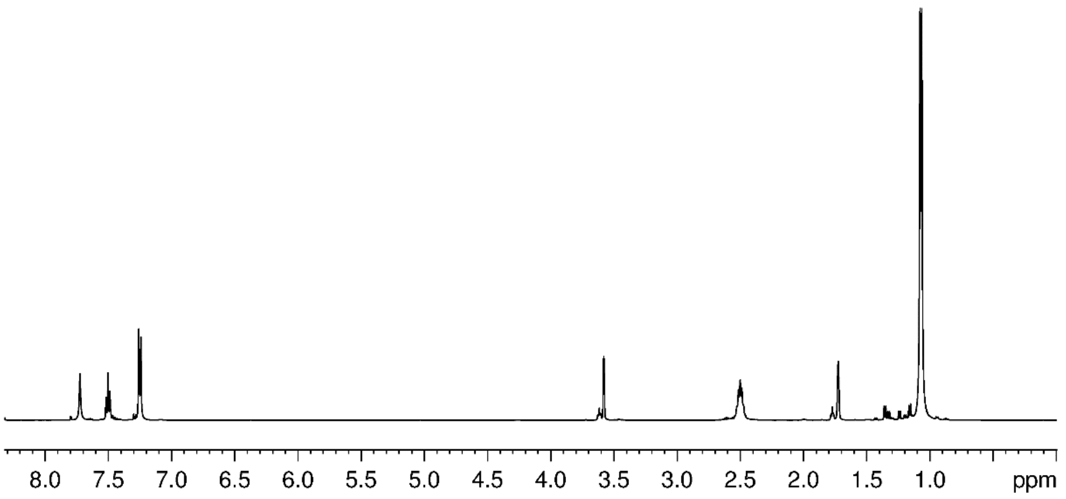

Figure S18. ^{1}H NMR spectrum of [**4**][SnBr$_5$(THF)] in d_8-THF at 338 K.

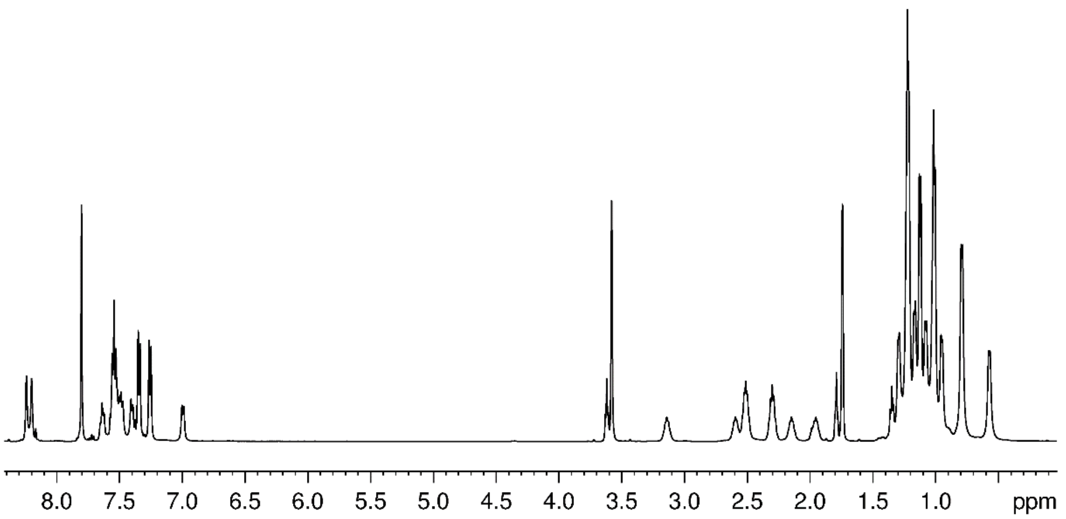

Figure S19. ^{1}H NMR spectrum of [**4**][SnBr$_5$(THF)] in d_8-THF at 208 K.

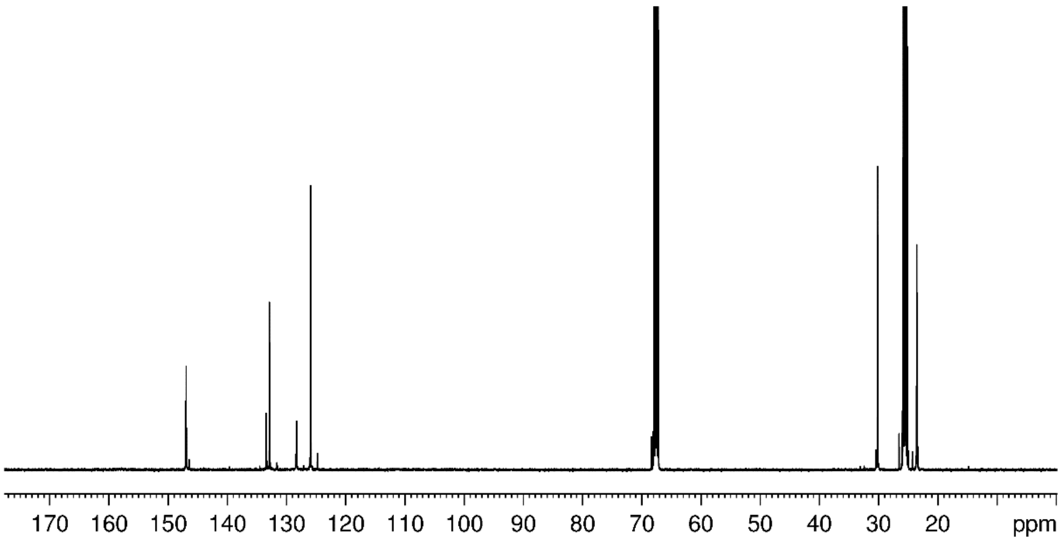

Figure S20. $^{13}C\{^{1}H\}$ NMR spectrum of [**4**][$SnBr_5$(THF)] in d_8-THF at 338 K.

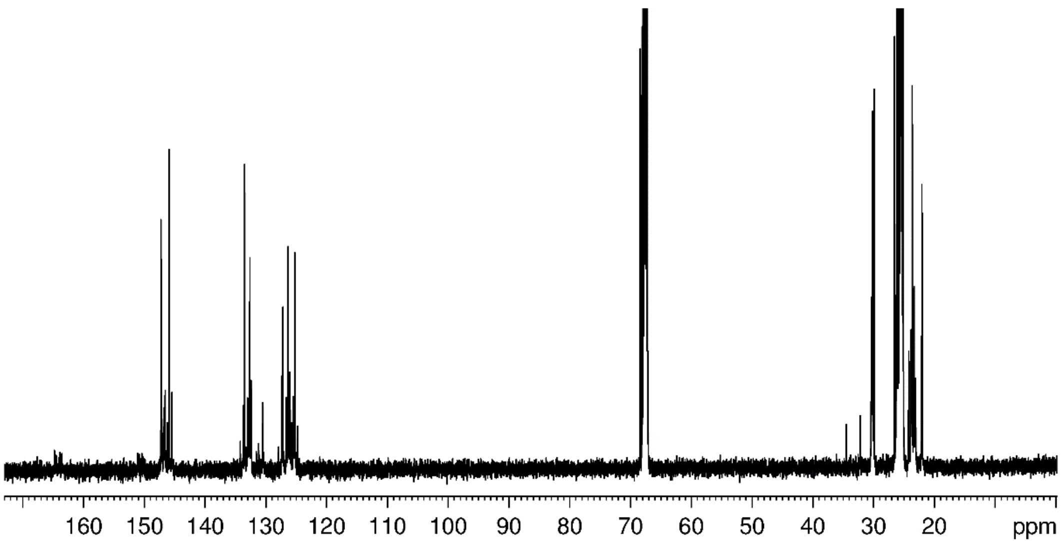

Figure S21. $^{13}C\{^{1}H\}$ NMR spectrum of [**4**][$SnBr_5$(THF)] in d_8-THF at 208 K.

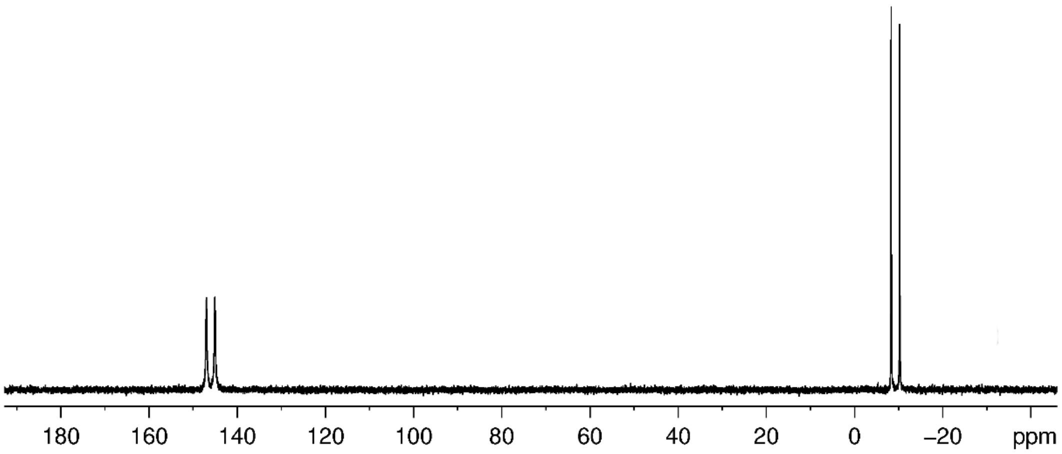

Figure S22. ^{31}P NMR spectrum of [**4**][$SnBr_5$(THF)] in d_8-THF at 208 K.

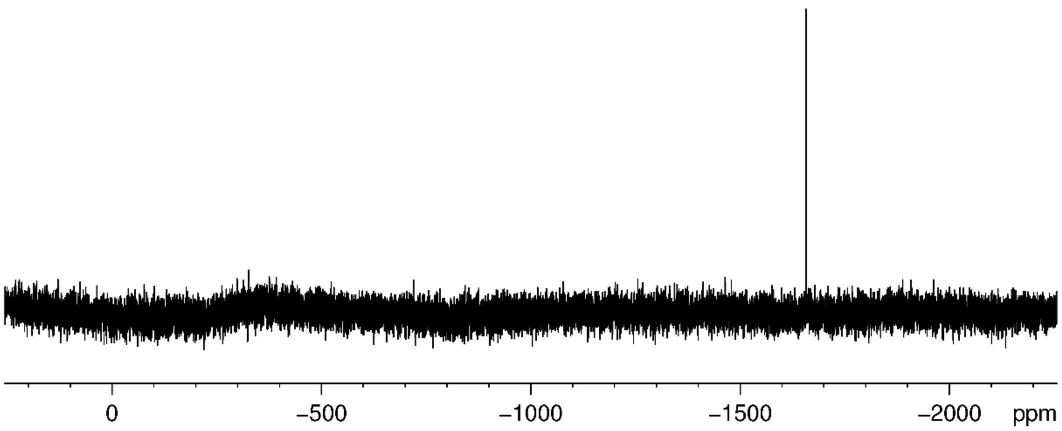

Figure S23. $^{119}Sn\{^1H\}$ NMR spectrum of [**4**][$SnBr_5$(THF)] in d_8-THF at 298 K.

4. ESI-MS spectra

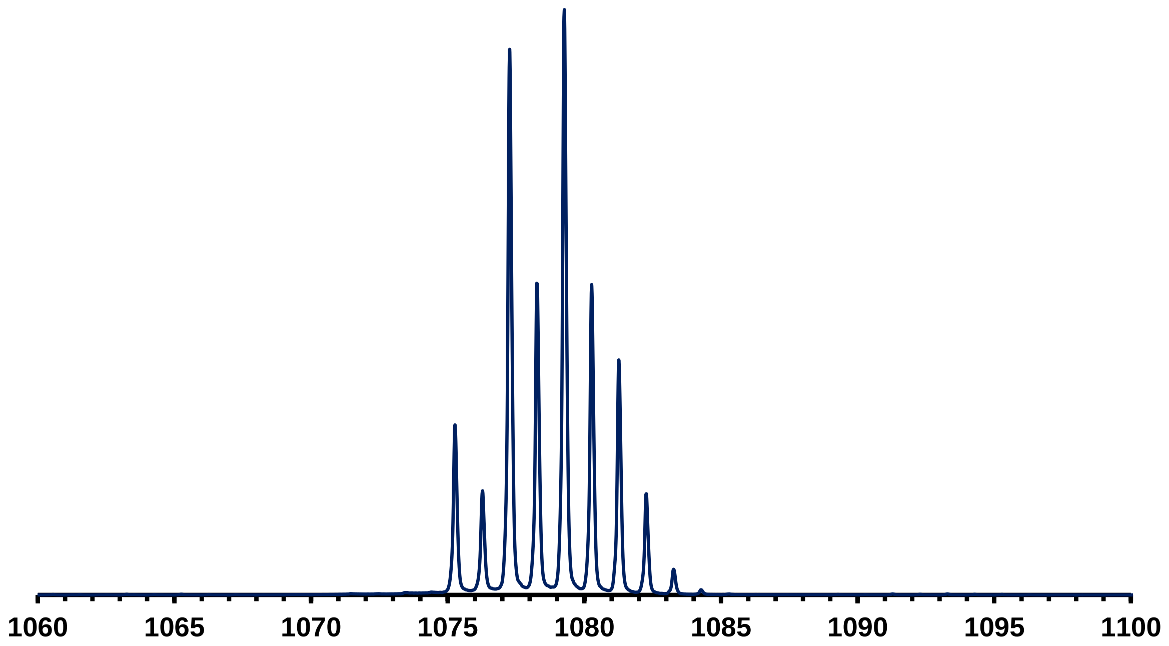

Figure S24. Mass envelope for the molecular ion observed in the positive ion mode ESI-MS spectrum of **2**.

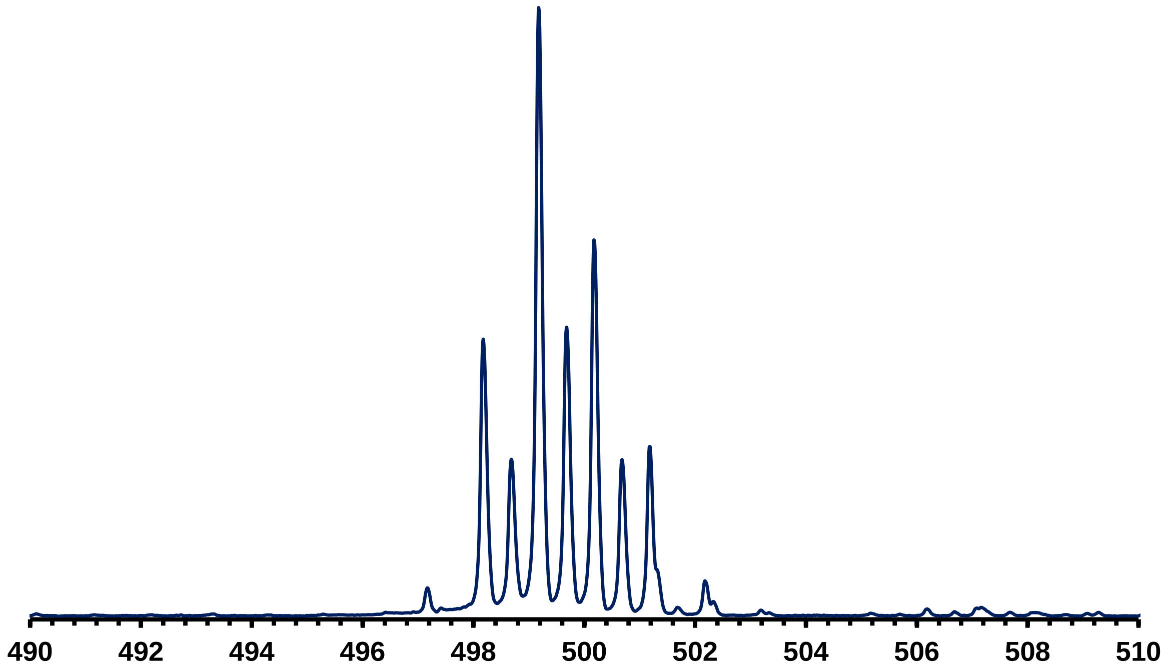

Figure S25. Mass envelope for the molecular ion observed in the positive ion mode ESI-MS spectrum of [**3**][$BAr^F{}_4$]$_2$.

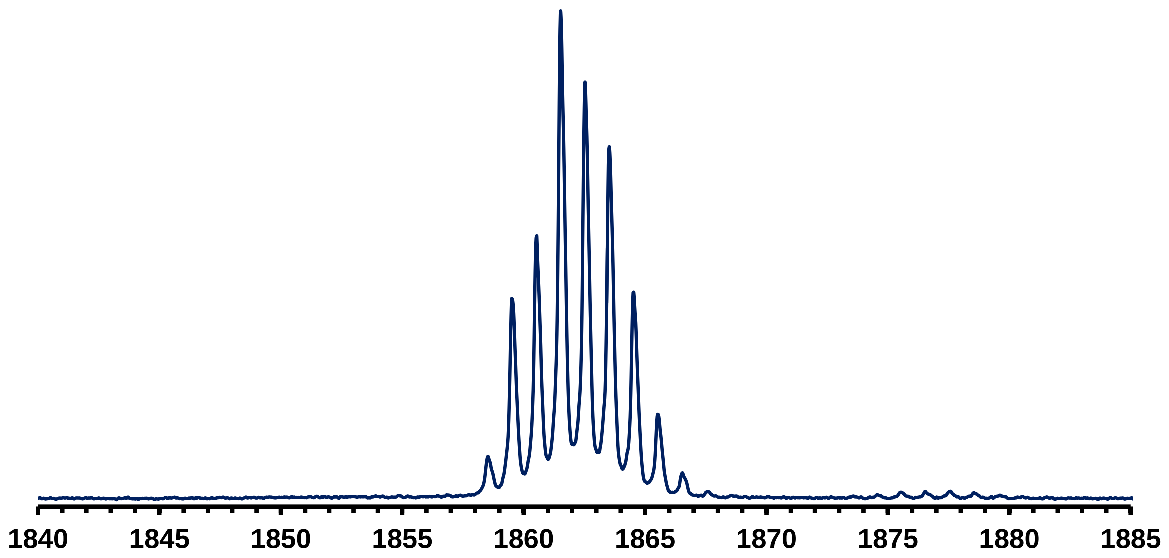

Figure S26. Mass envelope for the {[**3**][BAr$^F{}_4$]}$^+$ ion pair observed in the positive ion mode ESI-MS spectrum of [**3**][BAr$^F{}_4$]$_2$.

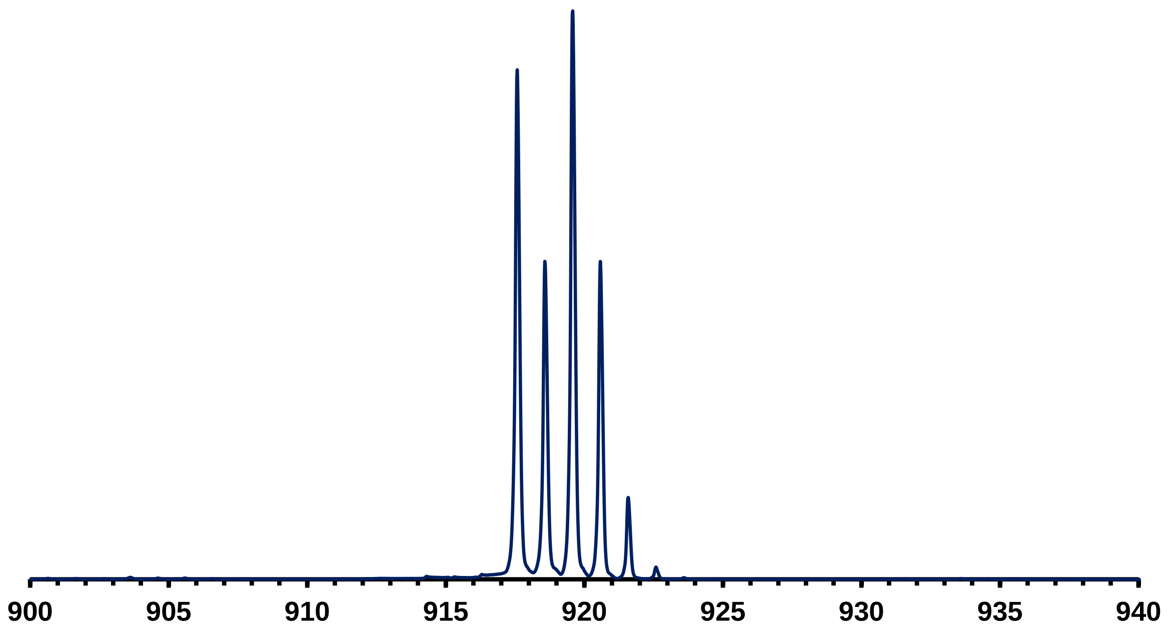

Figure S27. Mass envelope for the molecular ion observed in the positive ion mode ESI-MS spectrum of [**4**][SnBr$_5$(THF)].

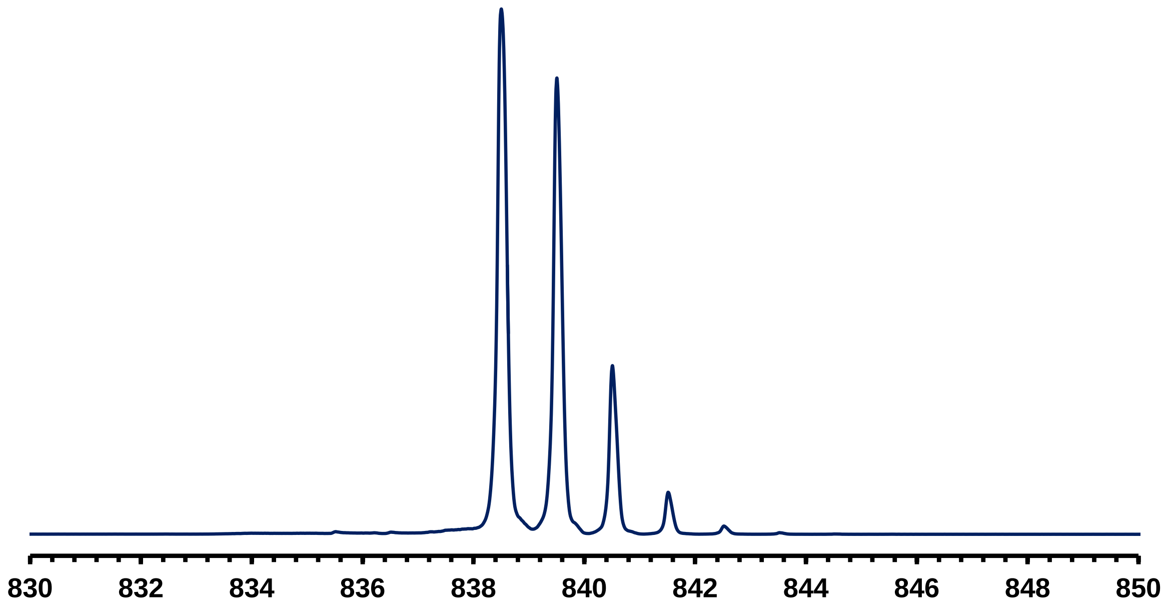

Figure S28. Mass envelope for the molecular ion observed in the positive ion mode ESI-MS spectrum of **5**.

5. UV/Vis spectra

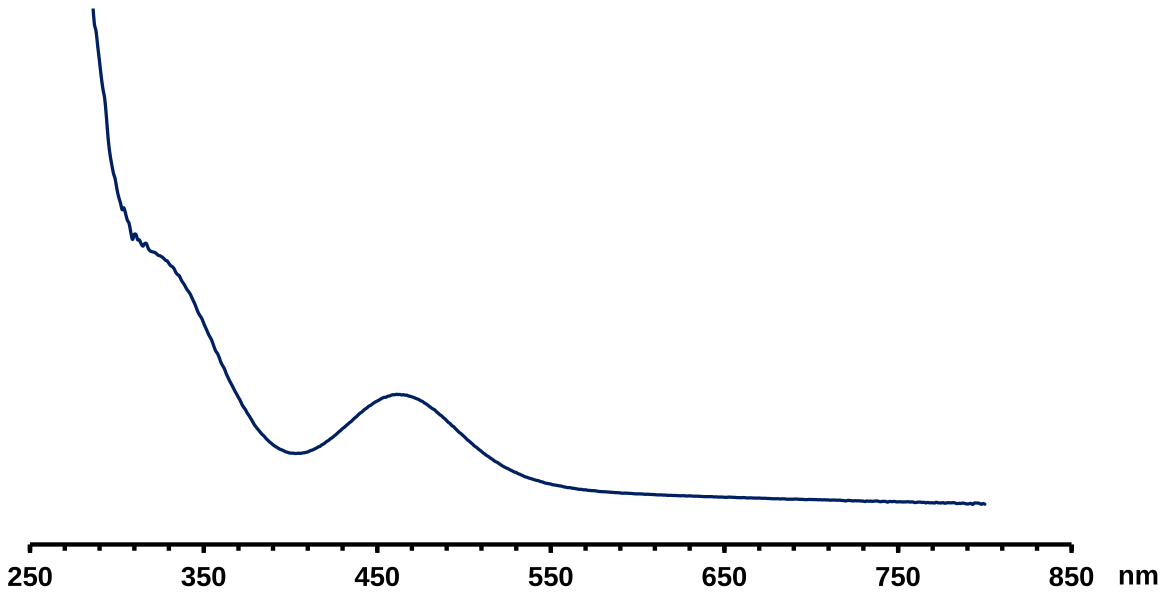

Figure S29. UV/Vis spectrum of [**2**]Br in fluorobenzene.

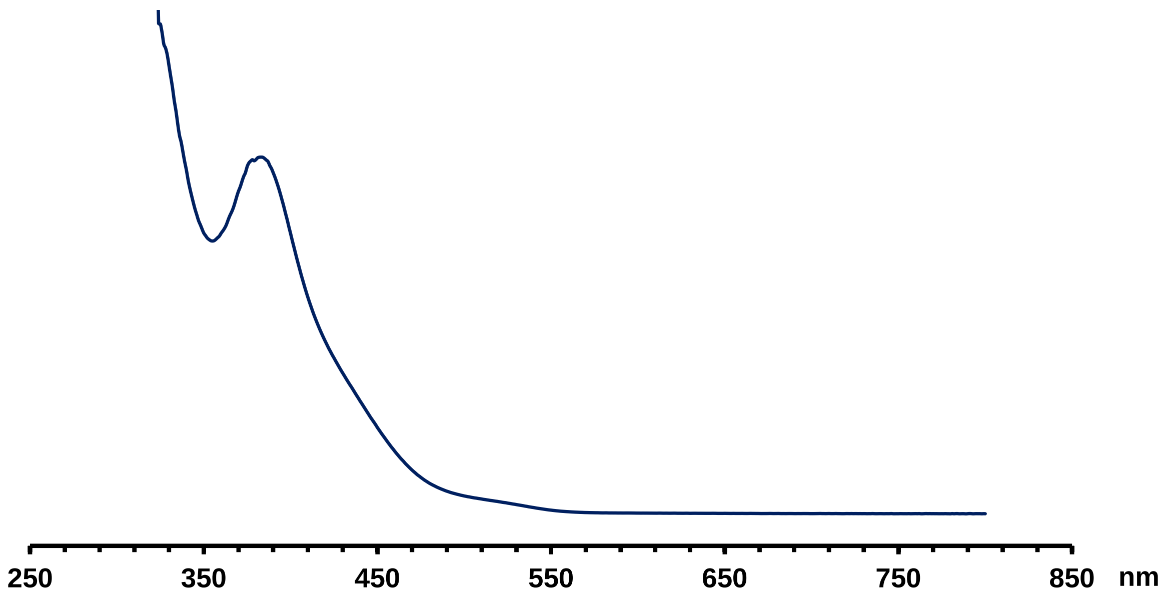

Figure S30. UV/Vis spectrum of [**3**][$BAr^F{}_4$]$_2$ in DFB.

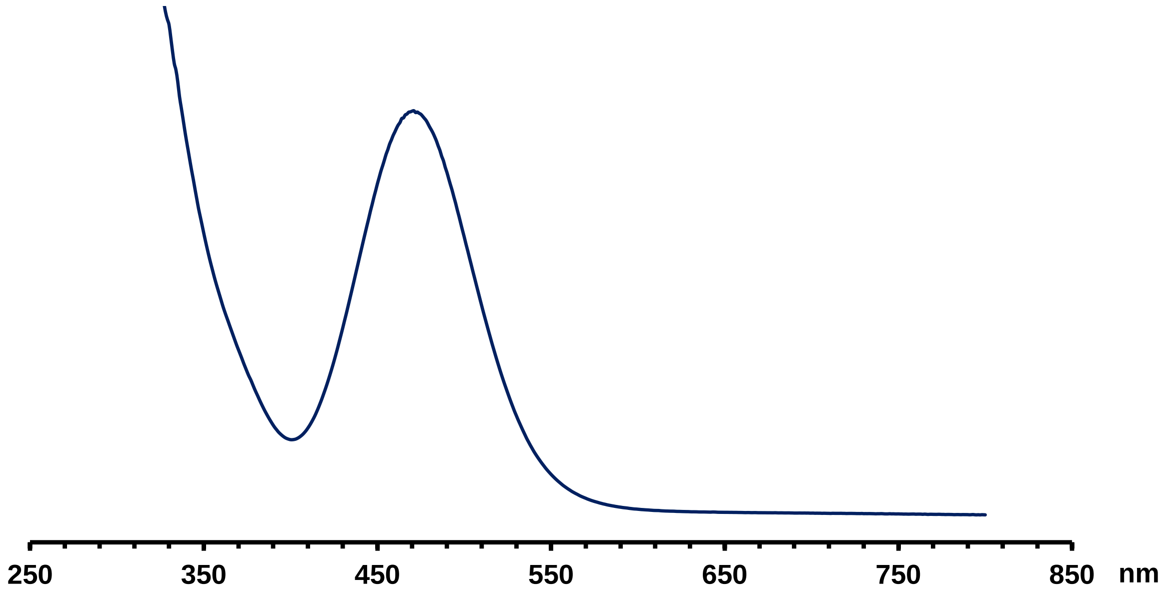

Figure S31. UV/Vis spectrum of [**4**][$SnBr_5$(THF)] in THF.

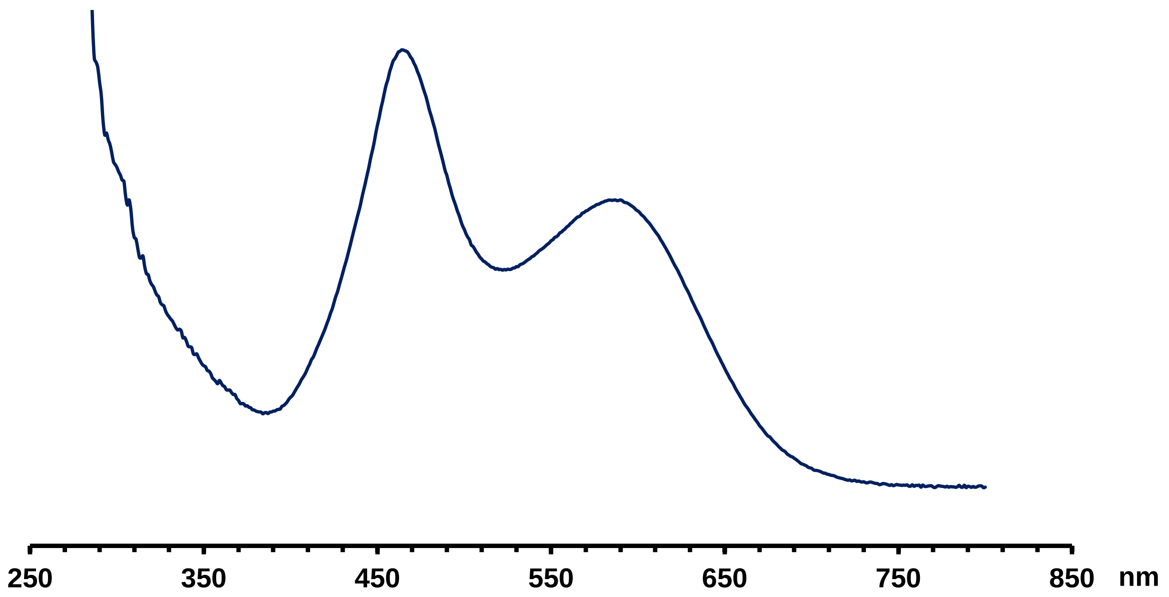

Figure S32. UV/Vis spectrum of [**5**][$BAr^F{}_4$] in fluorobenzene.

6. EPR spectra

EPR measurements were performed at the Centre for Advanced Electron Spin Resonance (CAESR) of the Chemistry Department of the University of Oxford. The X-band spectrometer was a Bruker-Biospin EMXplus with a PremiumX microwave bridge, and a Bruker BioSpin SHQE-W resonator.

The EPR spectra of **5** (Figure S31) are characteristic of the proposed molecular structure. The predominant spin density is located about the ^{31}P nuclei, giving rise to 1:2:1 hyperfine pattern (found 0.46:1.00:0.45), with unequal intensities due to slow tumbling of the molecule on the time scale of the microwave frequency.[8] This resonance has a g_{iso} value of 2.0090 (+/– 0.0001), consistent with the data previously reported by Bertrand and co-workers. The isotropic hyperfine for the ^{31}P nuclei, A_{iso}(^{31}P), is 126 MHz. The hyperfine interactions of the four ^{14}N atoms of the imidazolyl groups are resolved as a nine-peak pattern on the central peak, consistent with the hyperfine splitting rule of $2nI+1$, where n is the number of nuclei and I is the nuclear spin value. The ^{14}N isotropic hyperfine, A_{iso}(^{14}N), is 4.2 (+/–0.1) MHz. Further splitting within the central feature was observed by using a smaller field modulation value, 90 milliGauss (mG), but this is incompletely-resolved.

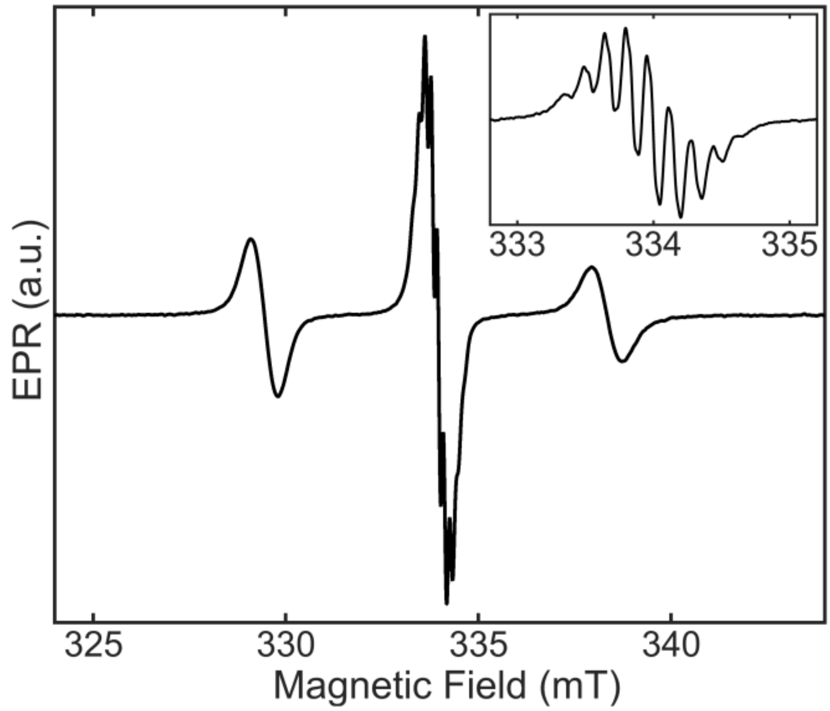

Figure S33. CW-EPR of [**5**][$BAr^F{}_4$] at X-band (ν = 9.3761 GHz) and room temperature, at a concentration of 100 μM in fluorobenzene. Non-saturating conditions were found at 5 mW, with 100kHz modulation amplitudes of 1 G and inset, 90 mG.

7. GC-MS spectra

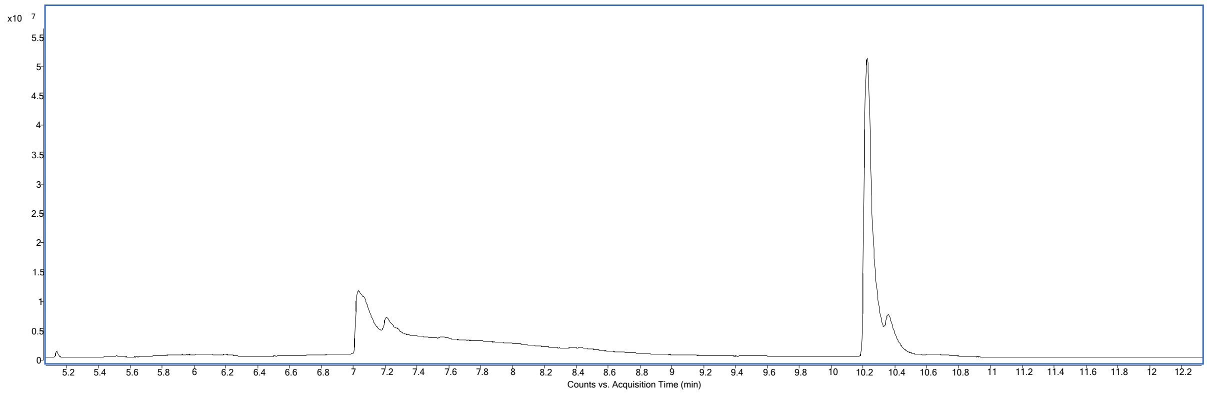

Figure S35. GC trace observed for THF solution of Br_2 heated at 65 °C overnight.

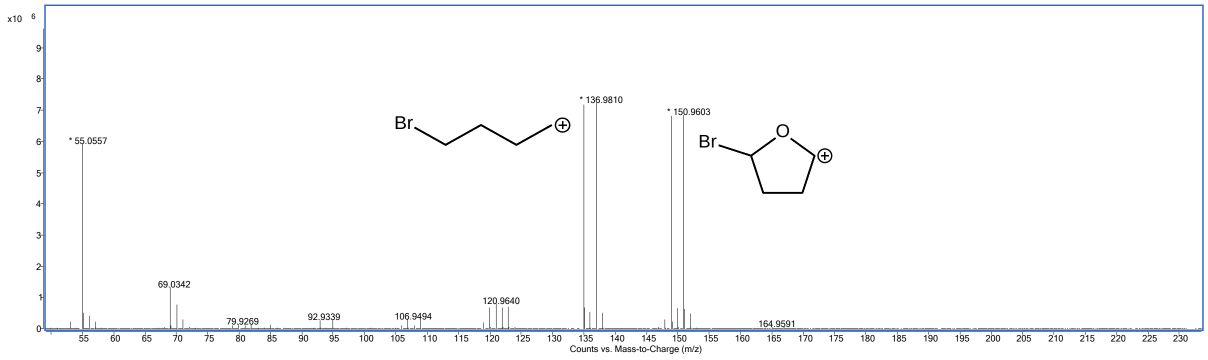

Figure S37. Positive ion mode EI-MS spectrum for the peak observed at 10.3 minutes.

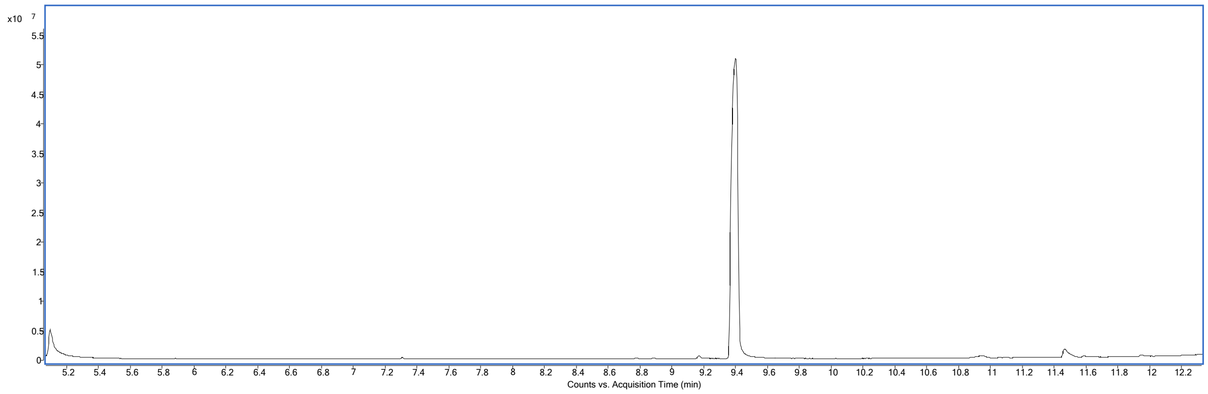

Figure S39. GC trace observed for the distilled reaction mixture of **1** heated in THF for three days at 65 °C.

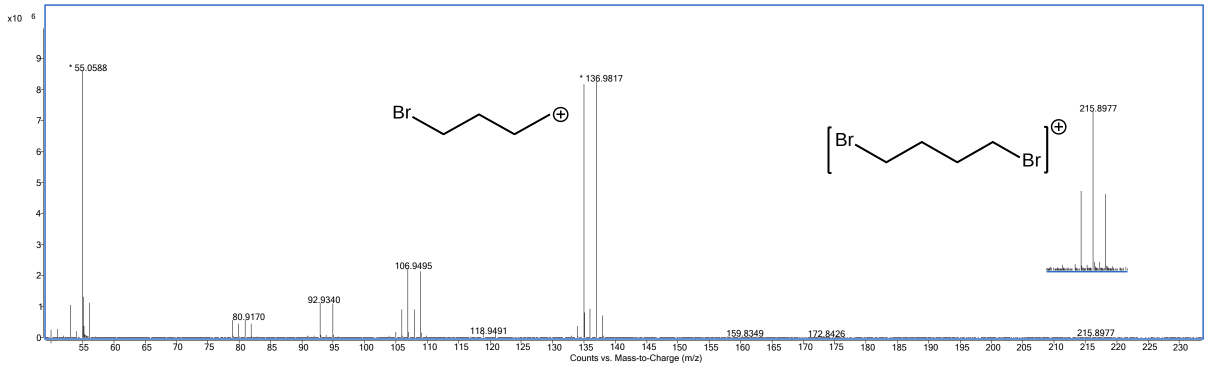

Figure S40. Positive ion mode EI-MS spectrum of the peak observed at 9.4 minutes.

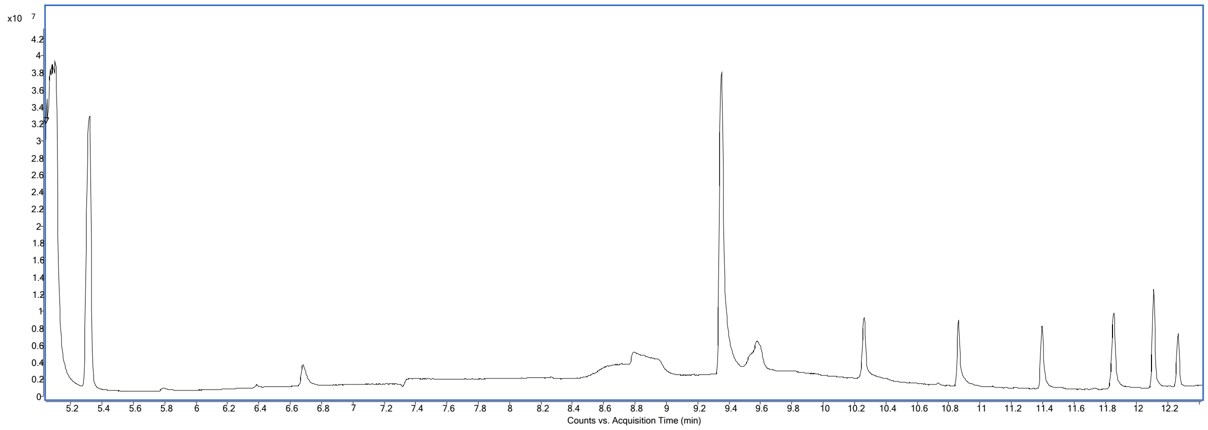

Figure S41. GC trace observed for the distilled reaction mixture of **1** heated in d_8-THF for three days at 65 °C. Peaks above 9.4 minutes were identified as oligosiloxane (grease) impurities.

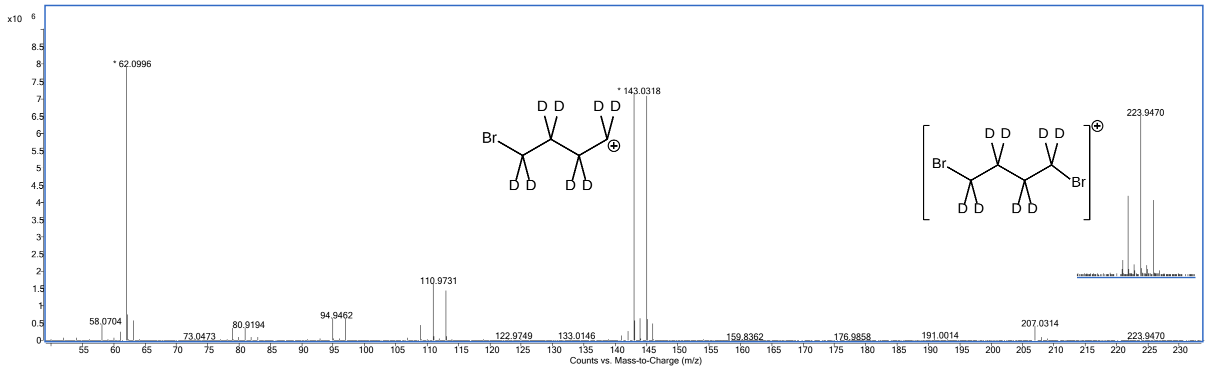

Figure S42. Positive ion mode EI-MS spectrum of the peak observed at 9.4 minutes.

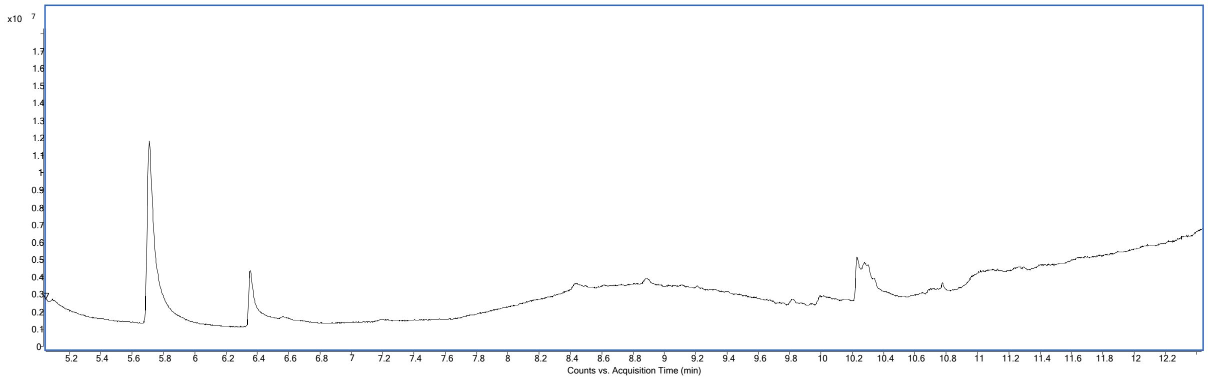

Figure S43. GC trace observed for the THF solution of the volatiles formed when **1** is heated at 140 °C and condensed onto room temperature THF.

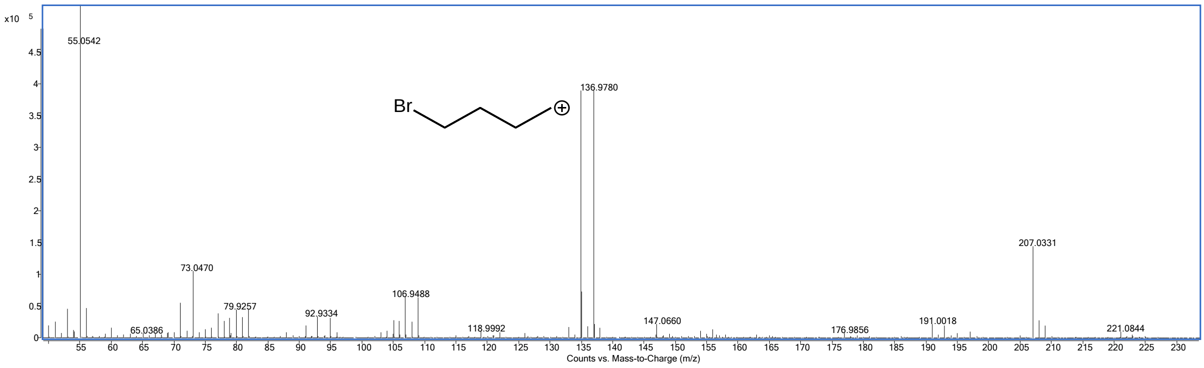

Figure S44. Positive ion mode EI-MS spectrum of the peak observed at 10.3 minutes.

8. TGA data

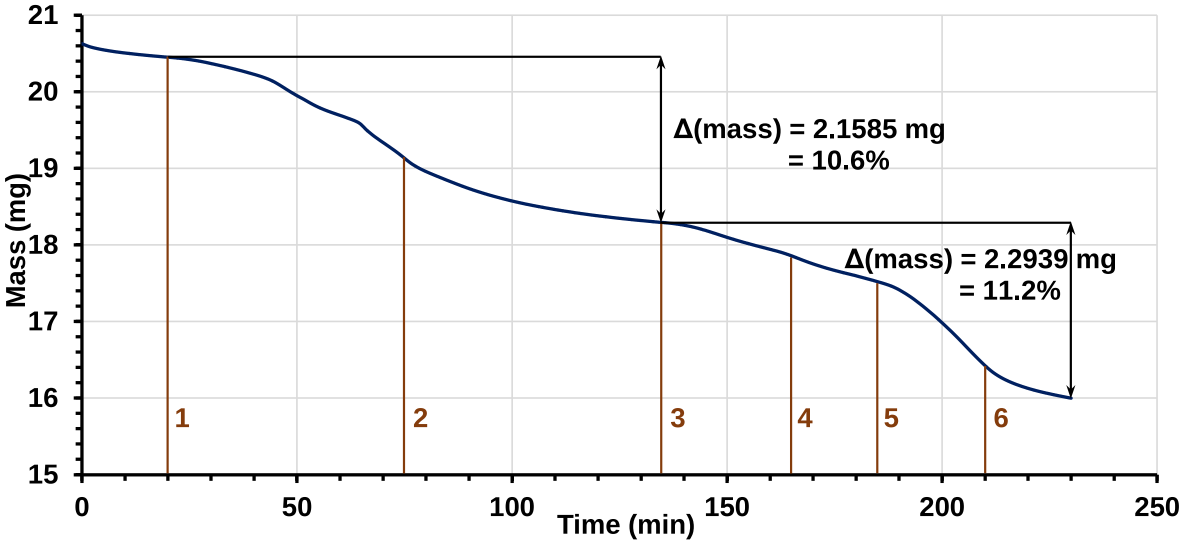

Figure S45. TGA plot of **1** observed with the following temperature program: 1 = Heat from 25 °C to 140 °C at 2 °C/min; 2 = Hold at 140 °C for 60 min; 3 = Heat from 140 °C to 200 °C at 2 °C/min; 4 = Hold at 200 °C for 20 min; 5 = Heat from 200 °C to 250 °C at 2 °C/min; 6 = Hold at 250 °C for 20 min.

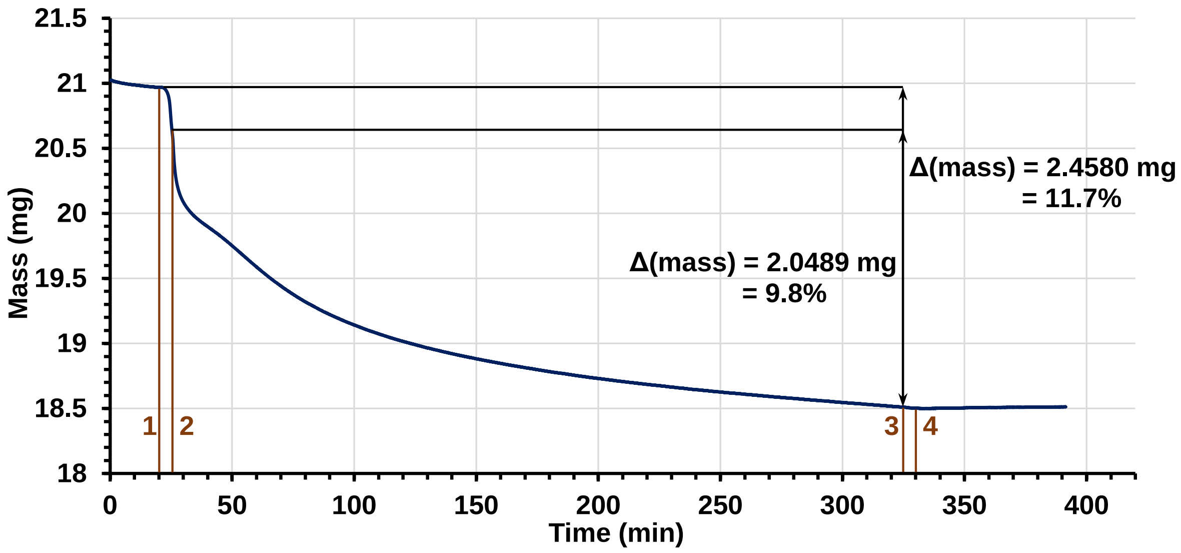

Figure S46. TGA plot of **1** observed with the following temperature program: 1 = Heat from 25 °C to 140 °C at 20 °C/min; 2 = Hold at 140 °C for 300 min; 3 = Cool from 140 °C to 25 °C at 20 °C/min; 4 = Hold at 25 °C for 60 min.

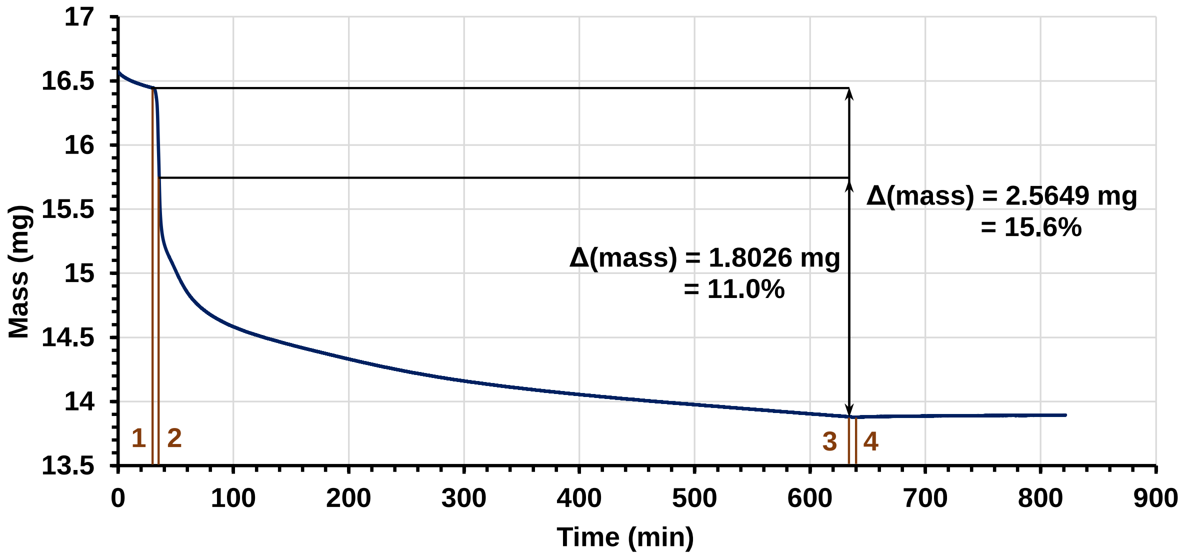

Figure S47. TGA plot of **1** observed with the following temperature program: 1 = Heat from 25 °C to 140 °C at 20 °C/min; 2 = Hold at 140 °C for 600 min; 3 = Cool from 140 °C to 25 °C at 20 °C/min; 4 = Hold at 25 °C for 60 min.

9. Computational details

All geometry optimizations were performed using the Amsterdam Density Functional package (ADF2014.01).[9] An TZ2P Slater-type basis set of triple-ζ quality, extended with two polarization functions, was used to describe all phosphorus and bromine atoms while a DZ basis set was used for all remaining atoms. Geometry optimizations were performed using the Becke88 exchange functional with Perdew86 local correlation functional.[10,11] The Grimme3 empirical dispersion correction was applied to all calculations.[12] All structures were optimized using the gradient algorithm of Versluis and Ziegler.[13] Stationary points were confirmed to be minima by the absence of imaginary frequencies.

Cartesian coordinates [Å] for the optimized computed geometry of 1.

**Atom**	***x***	***y***	***z***
1. P	0.147088	–0.016465	–0.039627
2. Br	2.705102	0.357376	–0.359257
3. Br	–2.344297	–0.072258	0.091529
4. Br	0.408807	–2.219357	0.411641
5. C	0.110576	0.039009	–1.927448
6. N	0.540139	–0.828322	–2.911630
7. C	0.642914	–0.156027	–4.140340
8. H	0.978743	–0.670368	–5.029749
9. C	0.235678	1.131702	–3.932778
10. H	0.162940	1.977739	–4.600337
11. N	–0.139258	1.233459	–2.584994
12. C	0.675961	–2.274076	–2.810069
13. C	1.960517	–2.848936	–2.837270
14. C	2.036488	–4.252981	–2.753600
15. H	3.012472	–4.738189	–2.759057
16. C	0.878463	–5.031495	–2.650378
17. H	0.958878	–6.117393	–2.574423
18. C	–0.386768	–4.426644	–2.651407
19. H	–1.277962	–5.048228	–2.578999
20. C	–0.519042	–3.031642	–2.734890
21. C	3.228104	–2.014792	–2.978394
22. H	2.966003	–0.959788	–2.806595
23. C	4.284651	–2.396919	–1.914480
24. H	5.106764	–1.664530	–1.928178
25. H	4.712114	–3.394790	–2.109173
26. H	3.838535	–2.385982	–0.910853
27. C	3.806224	–2.155776	–4.410757

28. H	4.691406	–1.509993	–4.526708
29. H	3.065781	–1.876404	–5.177247
30. H	4.108543	–3.197735	–4.606206
31. C	–1.895021	–2.374610	–2.802005
32. H	–1.827765	–1.374179	–2.351926
33. C	–2.975116	–3.116819	–1.988150
34. H	–3.880373	–2.491995	–1.936364
35. H	–2.630010	–3.291572	–0.958426
36. H	–3.255016	–4.078051	–2.450554
37. C	–2.314869	–2.221917	–4.289478
38. H	–3.317292	–1.771753	–4.361579
39. H	–2.340197	–3.210777	–4.778005
40. H	–1.606014	–1.586229	–4.844208
41. C	–0.945099	2.331039	–2.056960
42. C	–0.329468	3.341765	–1.294083
43. C	–1.168475	4.316404	–0.719797
44. H	–0.732850	5.102926	–0.104143
45. C	–2.550161	4.291285	–0.937573
46. H	–3.186841	5.046751	–0.474756
47. C	–3.123048	3.309580	–1.758653
48. H	–4.198450	3.317946	–1.932526
49. C	–2.336051	2.300583	–2.334817
50. C	1.184314	3.457791	–1.191111
51. H	1.631789	2.508134	–1.519911
52. C	1.672275	3.714994	0.251106
53. H	2.769261	3.635652	0.288748
54. H	1.259467	2.965934	0.941383
55. H	1.388260	4.720650	0.604812
56. C	1.668548	4.566386	–2.164124
57. H	1.334428	4.356931	–3.193281
58. H	2.768504	4.624119	–2.155334
59. H	1.261498	5.548521	–1.870061
60. C	–2.960009	1.274501	–3.281864
61. H	–2.295392	0.399701	–3.325672
62. C	–4.350095	0.770876	–2.826827
63. H	–4.676726	–0.052497	–3.481859
64. H	–5.112350	1.564019	–2.898500
65. H	–4.313120	0.403643	–1.792972
66. C	–3.060650	1.880656	–4.709592
67. H	–3.456307	1.133005	–5.415918
68. H	–2.084260	2.228122	–5.078593
69. H	–3.739766	2.749088	–4.701207

Cartesian coordinates [Å] for the optimized computed geometry of 2.

**Atom**	***x***	***y***	***z***
1. P	0.974842	–0.354990	–0.900526
2. P	–1.020730	0.194716	0.113274
3. Br	1.206434	0.636951	1.643671
4. Br	0.375861	–1.203217	–3.001523

5. Br	–2.655583	–0.246769	–1.509484
6. C	1.199168	–2.038356	–0.123970
7. N	0.357720	–3.081766	0.202831
8. C	1.105021	–4.223858	0.521354
9. H	0.621599	–5.146585	0.809260
10. C	2.427100	–3.892380	0.395187
11. H	3.326220	–4.476356	0.526395
12. N	2.478368	–2.546381	0.019883
13. C	–1.098370	–3.053857	0.302622
14. C	–1.858183	–3.605086	–0.751798
15. C	–3.256605	–3.618387	–0.592784
16. H	–3.882355	–4.032839	–1.382183
17. C	–3.855208	–3.101066	0.561339
18. H	–4.941561	–3.117654	0.660168
19. C	–3.070673	–2.583447	1.600371
20. H	–3.551163	–2.211838	2.504714
21. C	–1.667832	–2.564241	1.506675
22. C	–1.214783	–4.228599	–1.987329
23. H	–0.177121	–3.865440	–2.055295
24. C	–1.941320	–3.819685	–3.292157
25. H	–2.910751	–4.334166	–3.392363
26. H	–1.327816	–4.097654	–4.163504
27. H	–2.112892	–2.734938	–3.316197
28. C	–1.183350	–5.774056	–1.843389
29. H	–0.606181	–6.091161	–0.961577
30. H	–0.727469	–6.229744	–2.736911
31. H	–2.205513	–6.170722	–1.733202
32. C	–0.807731	–2.151285	2.700141
33. H	0.164753	–1.797499	2.326809
34. C	–1.418881	–0.997232	3.525783
35. H	–1.735046	–0.166557	2.880336
36. H	–0.665929	–0.605135	4.226160
37. H	–2.285991	–1.329789	4.119420
38. C	–0.547265	–3.391934	3.598581
39. H	0.042903	–3.101272	4.482036
40. H	0.007548	–4.173421	3.056999
41. H	–1.501042	–3.825418	3.939561
42. C	3.719712	–1.791329	–0.132447
43. C	4.286147	–1.680201	–1.422557
44. C	5.476686	–0.939474	–1.540243
45. H	5.944664	–0.828898	–2.517926
46. C	6.080539	–0.366598	–0.414403
47. H	7.007175	0.198762	–0.523547
48. C	5.517212	–0.535912	0.856721
49. H	6.017529	–0.107545	1.723898
50. C	4.323670	–1.259562	1.033369
51. C	3.723089	–2.437325	–2.620432
52. H	2.671290	–2.688330	–2.417713
53. C	3.757843	–1.612863	–3.927001
54. H	3.311753	–0.619312	–3.776325

55. H	3.178715	–2.130513	–4.707008
56. H	4.786814	–1.491742	–4.303641
57. C	4.501220	–3.772915	–2.779148
58. H	5.571746	–3.576802	–2.950730
59. H	4.107500	–4.345578	–3.633512
60. H	4.414436	–4.393615	–1.873367
61. C	3.788034	–1.537954	2.437205
62. H	2.706040	–1.730198	2.362632
63. C	3.999735	–0.351170	3.409744
64. H	5.046199	–0.295635	3.751975
65. H	3.369477	–0.487338	4.302178
66. H	3.731107	0.606952	2.943772
67. C	4.473419	–2.807322	3.017712
68. H	5.565782	–2.667464	3.050287
69. H	4.268606	–3.702753	2.414060
70. H	4.113961	–2.995348	4.041561
71. C	–0.979648	2.032182	–0.213187
72. N	–0.570555	2.827301	–1.263255
73. C	–0.997982	4.147779	–1.065146
74. H	–0.768511	4.923770	–1.781477
75. C	–1.681393	4.180033	0.120216
76. H	–2.187304	4.985898	0.631347
77. N	–1.650450	2.885103	0.647238
78. C	0.245095	2.436997	–2.408791
79. C	1.652990	2.412039	–2.238960
80. C	2.423384	2.066387	–3.363188
81. H	3.508220	2.027194	–3.275553
82. C	1.817824	1.807921	–4.599564
83. H	2.434770	1.553450	–5.462707
84. C	0.428909	1.893967	–4.742681
85. H	–0.022982	1.707652	–5.716022
86. C	–0.394897	2.209021	–3.646037
87. C	2.324330	2.869415	–0.944505
88. H	1.631993	2.697840	–0.106422
89. C	3.625310	2.098792	–0.623631
90. H	3.477428	1.013408	–0.703064
91. H	3.940241	2.316648	0.408103
92. H	4.451401	2.390763	–1.292520
93. C	2.598045	4.396887	–1.030210
94. H	3.222898	4.625540	–1.908474
95. H	3.125769	4.737608	–0.125716
96. H	1.662636	4.970494	–1.119149
97. C	–1.903372	2.350746	–3.828639
98. H	–2.375835	2.294899	–2.835622
99. C	–2.495155	1.215163	–4.699209
100. H	–2.256897	1.359918	–5.765629
101. H	–3.592445	1.207877	–4.604848
102. H	–2.111574	0.235419	–4.382946
103. C	–2.235301	3.734997	–4.450183
104. H	–1.905975	4.564281	–3.805649

105. H	–3.321784	3.830214	–4.606973
106. H	–1.732188	3.847638	–5.424094
107. C	–2.210080	2.518265	1.946049
108. C	–1.436208	2.774012	3.104567
109. C	–1.996412	2.396599	4.338768
110. H	–1.437631	2.570632	5.257547
111. C	–3.266393	1.810329	4.408336
112. H	–3.679045	1.526894	5.377471
113. C	–4.022373	1.612943	3.246514
114. H	–5.024583	1.192626	3.321842
115. C	–3.514700	1.976873	1.985259
116. C	–0.111475	3.531508	3.054942
117. H	0.333030	3.386551	2.057078
118. C	0.910465	3.041061	4.109589
119. H	1.910711	3.428706	3.862469
120. H	0.964687	1.944214	4.140894
121. H	0.656313	3.410124	5.116879
122. C	–0.377756	5.049771	3.261819
123. H	–1.041390	5.464181	2.490096
124. H	0.571183	5.608355	3.236418
125. H	–0.857317	5.220208	4.239381
126. C	–4.401390	1.916381	0.745116
127. H	–3.761514	1.936560	–0.149389
128. C	–5.254003	0.629868	0.676959
129. H	–4.631436	–0.263078	0.831023
130. H	–5.725559	0.549140	–0.314601
131. H	–6.062561	0.639282	1.426756
132. C	–5.295475	3.186248	0.710536
133. H	–5.937252	3.228061	1.604759
134. H	–5.938495	3.176970	–0.183448
135. H	–4.684469	4.102456	0.691447

Cartesian coordinates [Å] for the optimized computed geometry of 3 (1S,2R isomer).

Atom	*x*	*y*	*z*
1. P	1.128999	0.018916	–0.030781
2. P	–1.128999	–0.018916	0.030781
3. Br	1.286245	2.257015	–0.057718
4. Br	–1.286245	–2.257015	0.057718
5. C	1.367432	–0.371610	1.750106
6. N	2.361091	–1.279803	2.056384
7. C	1.408205	–0.740784	3.988157
8. H	1.104664	–0.616724	5.018739
9. C	2.390098	–1.526041	3.422724
10. H	3.102955	–2.205001	3.870951
11. N	0.794917	–0.023503	2.958324
12. C	3.321024	–1.734287	1.054127
13. C	4.407520	–0.870833	0.776447
14. C	5.271137	–1.261975	–0.262212
15. H	6.132735	–0.643365	–0.510278

16. C	5.048374	–2.450352	–0.970871
17. H	5.738572	–2.739990	–1.764380
18. C	3.974071	–3.290596	–0.646237
19. H	3.841463	–4.224561	–1.189490
20. C	3.082232	–2.954163	0.386910
21. C	4.701861	0.359454	1.634295
22. H	3.791033	0.629027	2.194890
23. C	5.112431	1.601978	0.813557
24. H	6.085656	1.460785	0.317912
25. H	5.215052	2.469963	1.483204
26. H	4.363233	1.844487	0.046276
27. C	5.789869	–0.015097	2.677877
28. H	5.474087	–0.873927	3.290459
29. H	5.995297	0.835614	3.345552
30. H	6.727751	–0.293522	2.172434
31. C	1.972702	–3.900581	0.832346
32. H	1.199898	–3.306415	1.350499
33. C	1.293918	–4.631895	–0.347057
34. H	0.995611	–3.924833	–1.133552
35. H	0.401257	–5.169304	0.009698
36. H	1.962061	–5.385126	–0.794069
37. C	2.554489	–4.916927	1.853864
38. H	3.350352	–5.516318	1.384964
39. H	1.766832	–5.599534	2.208550
40. H	2.992865	–4.409233	2.726541
41. C	–0.175449	1.049698	3.193471
42. C	0.335096	2.361009	3.365943
43. C	–0.602111	3.370039	3.651371
44. H	–0.260163	4.390642	3.814595
45. C	–1.970450	3.081541	3.745339
46. H	–2.674643	3.881594	3.976298
47. C	–2.435117	1.772112	3.583887
48. H	–3.498682	1.564124	3.686636
49. C	–1.541807	0.714850	3.329851
50. C	1.832256	2.672240	3.348730
51. H	2.327292	1.963193	2.665756
52. C	2.148867	4.100737	2.842729
53. H	1.571815	4.362491	1.943905
54. H	3.219241	4.179638	2.599907
55. H	1.939144	4.856941	3.615258
56. C	2.439511	2.472923	4.765601
57. H	1.904856	3.095177	5.499738
58. H	3.499766	2.769962	4.768206
59. H	2.380880	1.427466	5.099173
60. C	–2.032447	–0.730108	3.332387
61. H	–1.291525	–1.355478	2.806058
62. C	–3.401633	–0.900700	2.632398
63. H	–4.221703	–0.507732	3.254170
64. H	–3.605654	–1.968949	2.465573
65. H	–3.435925	–0.376013	1.666492

66. C	–2.118496	–1.243728	4.797515
67. H	–1.142584	–1.209809	5.303587
68. H	–2.480184	–2.283583	4.816915
69. H	–2.816449	–0.620748	5.378527
70. C	–1.367432	0.371610	–1.750106
71. N	–2.361091	1.279803	–2.056384
72. C	–1.408205	0.740784	–3.988157
73. H	–1.104664	0.616724	–5.018739
74. C	–2.390098	1.526041	–3.422724
75. H	–3.102955	2.205001	–3.870951
76. N	–0.794917	0.023503	–2.958324
77. C	–3.321024	1.734287	–1.054127
78. C	–4.407520	0.870833	–0.776447
79. C	–5.271137	1.261975	0.262212
80. H	–6.132735	0.643365	0.510278
81. C	–5.048374	2.450352	0.970871
82. H	–5.738572	2.739990	1.764380
83. C	–3.974071	3.290596	0.646237
84. H	–3.841463	4.224561	1.189490
85. C	–3.082232	2.954163	–0.386910
86. C	–4.701861	–0.359454	–1.634295
87. H	–3.791033	–0.629027	–2.194890
88. C	–5.112431	–1.601978	–0.813557
89. H	–6.085656	–1.460785	–0.317912
90. H	–5.215052	–2.469963	–1.483204
91. H	–4.363233	–1.844487	–0.046276
92. C	–5.789869	0.015097	–2.677877
93. H	–5.474087	0.873927	–3.290459
94. H	–5.995297	–0.835614	–3.345552
95. H	–6.727751	0.293522	–2.172434
96. C	–1.972702	3.900581	–0.832346
97. H	–1.199898	3.306415	–1.350499
98. C	–1.293918	4.631895	0.347057
99. H	–0.995611	3.924833	1.133552
100. H	–0.401257	5.169304	–0.009698
101. H	–1.962061	5.385126	0.794069
102. C	–2.554489	4.916927	–1.853864
103. H	–3.350352	5.516318	–1.384964
104. H	–1.766832	5.599534	–2.208550
105. H	–2.992865	4.409233	–2.726541
106. C	0.175449	–1.049698	–3.193471
107. C	–0.335096	–2.361009	–3.365943
108. C	0.602111	–3.370039	–3.651371
109. H	0.260163	–4.390642	–3.814595
110. C	1.970450	–3.081541	–3.745339
111. H	2.674643	–3.881594	–3.976298
112. C	2.435117	–1.772112	–3.583887
113. H	3.498682	–1.564124	–3.686636
114. C	1.541807	–0.714850	–3.329851
115. C	–1.832256	–2.672240	–3.348730

116. H	–2.327292	–1.963193	–2.665756
117. C	–2.148867	–4.100737	–2.842729
118. H	–1.571815	–4.362491	–1.943905
119. H	–3.219241	–4.179638	–2.599907
120. H	–1.939144	–4.856941	–3.615258
121. C	–2.439511	–2.472923	–4.765601
122. H	–1.904856	–3.095177	–5.499738
123. H	–3.499766	–2.769962	–4.768206
124. H	–2.380880	–1.427466	–5.099173
125. C	2.032447	0.730108	–3.332387
126. H	1.291525	1.355478	–2.806058
127. C	3.401633	0.900700	–2.632398
128. H	4.221703	0.507732	–3.254170
129. H	3.605654	1.968949	–2.465573
130. H	3.435925	0.376013	–1.666492
131. C	2.118496	1.243728	–4.797515
132. H	1.142584	1.209809	–5.303587
133. H	2.480184	2.283583	–4.816915
134. H	2.816449	0.620748	–5.378527

TOTAL BONDING ENERGY: –73042.37 kJ mol^{-1}

Cartesian coordinates [Å] for the optimized computed geometry of 3 (1R,2R isomer).

**Atom**	***x***	***y***	***z***
1. P	1.236816	–0.015553	0.009322
2. P	–1.018313	–0.263556	–0.201080
3. Br	2.244374	–1.575860	–1.179000
4. Br	–1.384261	–2.442450	–0.524226
5. C	1.310903	–0.725104	1.732704
6. N	2.127639	–1.707634	2.266225
7. C	1.108704	–0.766135	4.007923
8. H	0.749408	–0.430154	4.970718
9. C	1.993595	–1.746637	3.656976
10. H	2.575979	–2.439521	4.250450
11. N	0.697695	–0.141987	2.826529
12. C	3.166454	–2.496753	1.615108
13. C	4.445939	–1.909063	1.514718
14. C	5.440569	–2.677482	0.882577
15. H	6.447206	–2.275574	0.771565
16. C	5.160161	–3.970275	0.417079
17. H	5.951739	–4.558197	–0.048843
18. C	3.880571	–4.528005	0.568339
19. H	3.695519	–5.540985	0.213436
20. C	2.844277	–3.794012	1.168466
21. C	4.751447	–0.507057	2.050029
22. H	3.944244	–0.213788	2.743136
23. C	4.795062	0.532662	0.899254
24. H	5.567900	0.262185	0.162103
25. H	5.033129	1.531260	1.297830
26. H	3.833578	0.594475	0.365737
27. C	6.068272	–0.479008	2.864496

28. H	6.089781	–1.288706	3.609141
29. H	6.165385	0.482362	3.391740
30. H	6.949225	–0.587003	2.214646
31. C	1.440297	–4.365810	1.339499
32. H	0.742966	–3.518604	1.460170
33. C	0.976121	–5.176772	0.109216
34. H	1.120277	–4.608055	–0.820810
35. H	–0.091165	–5.429787	0.207464
36. H	1.523231	–6.128262	0.020456
37. C	1.372472	–5.228685	2.628052
38. H	2.086657	–6.064365	2.565632
39. H	0.362341	–5.644954	2.761645
40. H	1.623643	–4.639143	3.522706
41. C	–0.148287	1.045600	2.883829
42. C	0.504862	2.300155	2.920196
43. C	–0.285700	3.412190	3.259132
44. H	0.175841	4.395545	3.344644
45. C	–1.657832	3.269323	3.512063
46. H	–2.244845	4.142267	3.800734
47. C	–2.277427	2.015743	3.420630
48. H	–3.343851	1.927968	3.623573
49. C	–1.525766	0.861345	3.136899
50. C	2.012030	2.440340	2.709947
51. H	2.387460	1.523413	2.230864
52. C	2.360543	3.609677	1.762213
53. H	1.781170	3.541827	0.830738
54. H	3.431153	3.583853	1.508958
55. H	2.158217	4.587166	2.226243
56. C	2.732511	2.570362	4.076316
57. H	2.395961	3.472674	4.609767
58. H	3.821050	2.644484	3.930691
59. H	2.526364	1.700903	4.719640
60. C	–2.154657	–0.528575	3.220487
61. H	–1.450816	–1.258799	2.782342
62. C	–3.477606	–0.618747	2.432022
63. H	–4.267251	–0.004244	2.890359
64. H	–3.835859	–1.658875	2.420245
65. H	–3.347166	–0.275026	1.398593
66. C	–2.388159	–0.921468	4.705801
67. H	–1.455934	–0.918907	5.288054
68. H	–2.827794	–1.928633	4.768759
69. H	–3.080847	–0.212109	5.185047
70. C	–1.410146	0.368784	–1.893180
71. N	–2.680307	0.884303	–2.088326
72. C	–1.743330	0.830874	–4.097365
73. H	–1.470277	0.899245	–5.142266
74. C	–2.899699	1.159222	–3.432871
75. H	–3.831317	1.588466	–3.778487
76. N	–0.838838	0.352379	–3.148746
77. C	–3.616298	1.236812	–1.030901

78. C	–4.674565	0.351024	–0.739210
79. C	–5.586544	0.769797	0.247108
80. H	–6.437900	0.138837	0.499109
81. C	–5.413441	1.991386	0.911689
82. H	–6.135209	2.296287	1.670922
83. C	–4.350160	2.847135	0.584588
84. H	–4.263743	3.810276	1.085968
85. C	–3.441555	2.505220	–0.431112
86. C	–4.867360	–0.953443	–1.509914
87. H	–3.925987	–1.186334	–2.037724
88. C	–5.192106	–2.154181	–0.591027
89. H	–6.174458	–2.040391	–0.107784
90. H	–5.228778	–3.078540	–1.187950
91. H	–4.432018	–2.278390	0.191692
92. C	–5.978361	–0.761878	–2.578331
93. H	–5.742964	0.066760	–3.263969
94. H	–6.102833	–1.680416	–3.172155
95. H	–6.940383	–0.529938	–2.095301
96. C	–2.364772	3.478764	–0.921426
97. H	–2.047256	3.157887	–1.928184
98. C	–1.121831	3.470055	–0.003506
99. H	–0.725472	2.453443	0.156736
100. H	–0.324806	4.098218	–0.430258
101. H	–1.381468	3.868756	0.984946
102. C	–2.920441	4.916503	–1.070751
103. H	–3.115026	5.380249	–0.092125
104. H	–2.188717	5.550541	–1.594167
105. H	–3.858367	4.919671	–1.645903
106. C	0.514217	–0.016263	–3.538290
107. C	0.696620	–1.274797	–4.146419
108. C	1.977040	–1.547495	–4.663546
109. H	2.168792	–2.498831	–5.159296
110. C	3.006881	–0.600584	–4.566496
111. H	3.985357	–0.821754	–4.995508
112. C	2.788616	0.635971	–3.939791
113. H	3.604084	1.355051	–3.882190
114. C	1.528222	0.963911	–3.408885
115. C	–0.432494	–2.295424	–4.265715
116. H	–1.282239	–1.948074	–3.653622
117. C	–0.005061	–3.680974	–3.720111
118. H	0.411122	–3.593115	–2.706862
119. H	–0.874429	–4.356016	–3.684870
120. H	0.751442	–4.153606	–4.366283
121. C	–0.913264	–2.400923	–5.736754
122. H	–0.095032	–2.742566	–6.389253
123. H	–1.740918	–3.121751	–5.819362
124. H	–1.260628	–1.429245	–6.120617
125. C	1.249211	2.342132	–2.805644
126. H	0.426906	2.244920	–2.076546
127. C	2.464235	2.928272	–2.051371

128. H	3.267502	3.224645	–2.743564
129. H	2.160680	3.837380	–1.511680
130. H	2.878836	2.214082	–1.323908
131. C	0.779500	3.321554	–3.915923
132. H	–0.145610	2.977325	–4.402018
133. H	0.593143	4.320804	–3.493093
134. H	1.555490	3.414267	–4.692044

TOTAL BONDING ENERGY: –73013.41 kJ mol^{-1}

Cartesian coordinates [Å] for the optimized computed geometry of 4.

**Atom**	***x***	***y***	***z***
1. P	1.043938	0.024603	–0.055225
2. P	–1.071663	–0.010479	0.134760
3. Br	1.956958	–0.915500	1.984664
4. C	1.374821	1.779267	0.393470
5. N	0.669598	2.780351	1.033975
6. C	1.464119	3.921432	1.180488
7. H	1.076870	4.820240	1.641429
8. C	2.683393	3.640684	0.614999
9. H	3.565834	4.250198	0.479120
10. N	2.617679	2.330112	0.138907
11. C	–0.743710	2.733321	1.374153
12. C	–1.662748	3.195790	0.406317
13. C	–3.028939	3.092658	0.725476
14. H	–3.776757	3.445955	0.015443
15. C	–3.439827	2.532931	1.941714
16. H	–4.503663	2.448172	2.165630
17. C	–2.498872	2.093857	2.882033
18. H	–2.840971	1.657547	3.818590
19. C	–1.121444	2.193839	2.622119
20. C	–1.220420	3.787828	–0.930341
21. H	–0.121180	3.737021	–0.989568
22. C	–1.778311	2.976708	–2.125680
23. H	–1.466300	1.925146	–2.054674
24. H	–1.406615	3.399712	–3.072219
25. H	–2.878977	3.010371	–2.153902
26. C	–1.617798	5.283610	–1.013384
27. H	–2.713344	5.401433	–1.018077
28. H	–1.222667	5.735460	–1.936934
29. H	–1.223663	5.843339	–0.149442
30. C	–0.075754	1.772946	3.645489
31. H	0.834392	1.471532	3.103860
32. C	–0.528139	0.558447	4.479375
33. H	–1.303539	0.832049	5.213978
34. H	0.328190	0.150020	5.036996
35. H	–0.928499	–0.226270	3.823839
36. C	0.280386	2.979095	4.554747
37. H	0.688793	3.817561	3.969013
38. H	1.031712	2.682973	5.303973

39. H	–0.618557	3.338266	5.081059
40. C	3.644357	1.692837	–0.672396
41. C	3.473055	1.735961	–2.075001
42. C	4.460064	1.110454	–2.857749
43. H	4.367367	1.111968	–3.943745
44. C	5.573979	0.506734	–2.258504
45. H	6.338600	0.042073	–2.882532
46. C	5.721668	0.504226	–0.864742
47. H	6.597354	0.033476	–0.419605
48. C	4.751085	1.092876	–0.033128
49. C	2.300747	2.461309	–2.735912
50. H	1.664544	2.896091	–1.949645
51. C	1.426867	1.485609	–3.555015
52. H	1.995732	1.055383	–4.392927
53. H	0.556441	2.013794	–3.973109
54. H	1.067441	0.651947	–2.933754
55. C	2.809051	3.639854	–3.604127
56. H	3.447530	4.314963	–3.013432
57. H	1.957400	4.215931	–3.999180
58. H	3.399858	3.277776	–4.459256
59. C	4.923617	1.132273	1.481651
60. H	3.930512	1.292030	1.932867
61. C	5.487180	–0.191455	2.049636
62. H	4.934286	–1.056369	1.658481
63. H	5.393914	–0.192826	3.146661
64. H	6.555946	–0.311737	1.809389
65. C	5.838642	2.323639	1.875680
66. H	6.830533	2.215932	1.407873
67. H	5.970543	2.356968	2.968599
68. H	5.418129	3.286988	1.549477
69. C	–1.196207	–1.736490	–0.397315
70. N	–0.684437	–2.495597	–1.440276
71. C	–1.202337	–3.804001	–1.397099
72. H	–0.921332	–4.538552	–2.139696
73. C	–2.077199	–3.863222	–0.346898
74. H	–2.705326	–4.666223	0.011762
75. N	–2.067938	–2.601865	0.259016
76. C	0.094385	–2.040371	–2.580046
77. C	–0.606082	–1.435696	–3.653779
78. C	0.103434	–1.255307	–4.853672
79. H	–0.399522	–0.827124	–5.719461
80. C	1.453662	–1.621030	–4.954579
81. H	1.975912	–1.498143	–5.904793
82. C	2.138983	–2.125213	–3.843388
83. H	3.198722	–2.363968	–3.926181
84. C	1.468496	–2.354511	–2.626305
85. C	–2.071674	–1.013524	–3.520952
86. H	–2.231846	–0.701864	–2.475640
87. C	–2.419800	0.209064	–4.402101
88. H	–1.668498	1.004681	–4.298834

89. H	–3.395465	0.617867	–4.096214
90. H	–2.498378	–0.064712	–5.466488
91. C	–3.031958	–2.190910	–3.837809
92. H	–2.857243	–2.553630	–4.864142
93. H	–4.079734	–1.858442	–3.760942
94. H	–2.888763	–3.035139	–3.147772
95. C	2.202402	–2.952706	–1.428507
96. H	1.581403	–2.798964	–0.529693
97. C	3.559189	–2.249416	–1.184054
98. H	4.297336	–2.505400	–1.961280
99. H	3.971063	–2.561176	–0.212787
100. H	3.438817	–1.157927	–1.171097
101. C	2.395617	–4.481694	–1.608543
102. H	1.429461	–5.005924	–1.663643
103. H	2.961066	–4.893675	–0.758252
104. H	2.953181	–4.695067	–2.534678
105. C	–2.877305	–2.162637	1.378058
106. C	–4.031311	–1.401606	1.087100
107. C	–4.731342	–0.856874	2.178002
108. H	–5.633121	–0.269398	2.002463
109. C	–4.294854	–1.081063	3.490786
110. H	–4.854944	–0.658268	4.326804
111. C	–3.170720	–1.881637	3.745102
112. H	–2.872952	–2.072846	4.775578
113. C	–2.432336	–2.445567	2.689786
114. C	–4.546907	–1.232661	–0.343256
115. H	–3.840966	–1.730045	–1.026783
116. C	–4.641553	0.251292	–0.770940
117. H	–5.368358	0.798212	–0.148223
118. H	–4.975978	0.320638	–1.818742
119. H	–3.666882	0.751533	–0.676150
120. C	–5.908785	–1.958472	–0.497554
121. H	–5.826842	–3.010495	–0.180969
122. H	–6.240378	–1.930722	–1.547707
123. H	–6.685667	–1.479125	0.119328
124. C	–1.290752	–3.432691	2.933240
125. H	–0.657723	–3.454522	2.029563
126. C	–0.375247	–3.078624	4.123779
127. H	0.117093	–2.111850	3.970157
128. H	0.409527	–3.845469	4.221231
129. H	–0.928872	–3.054081	5.075984
130. C	–1.911465	–4.845407	3.137368
131. H	–2.503851	–4.863635	4.066592
132. H	–1.120410	–5.607688	3.211717
133. H	–2.587977	–5.116573	2.313005

Cartesian coordinates [Å] for the optimized computed geometry of 5.

**Atom**	***x***	***y***	***z***

1. P	–1.039274	–0.009221	–0.099454
2. P	1.082770	–0.076138	0.108133
3. C	–1.376031	1.735004	–0.096979
4. N	–0.595133	2.890837	–0.121899
5. C	–1.404436	4.037762	–0.075764
6. H	–0.971511	5.028725	–0.100596
7. C	–2.705649	3.616325	–0.024863
8. H	–3.636833	4.166933	–0.004267
9. N	–2.691483	2.214821	–0.039064
10. C	0.828010	2.919284	–0.375882
11. C	1.714763	3.026969	0.718798
12. C	3.088512	3.084719	0.426112
13. H	3.809527	3.185725	1.237014
14. C	3.543763	2.997864	–0.896866
15. H	4.614037	3.046146	–1.103385
16. C	2.639444	2.845948	–1.957326
17. H	3.016157	2.773262	–2.977787
18. C	1.252921	2.811454	–1.719790
19. C	1.206335	3.060125	2.157074
20. H	0.143503	2.762983	2.143895
21. C	1.962548	2.052343	3.060276
22. H	1.959416	1.045087	2.618041
23. H	1.479887	1.997507	4.048661
24. H	3.010383	2.352537	3.218645
25. C	1.291574	4.501848	2.722431
26. H	2.338583	4.844372	2.749728
27. H	0.887486	4.538438	3.746119
28. H	0.722216	5.207439	2.096503
29. C	0.262512	2.683729	–2.877947
30. H	–0.752011	2.585332	–2.462085
31. C	0.531413	1.415891	–3.721308
32. H	–0.211742	1.330959	–4.528878
33. H	0.466353	0.510744	–3.101587
34. H	1.528024	1.450533	–4.187712
35. C	0.283214	3.968806	–3.744214
36. H	1.267936	4.109952	–4.217741
37. H	0.072690	4.857904	–3.128870
38. H	–0.475050	3.904571	–4.540909
39. C	–3.873029	1.382006	–0.149291
40. C	–4.404886	0.785580	1.018471
41. C	–5.572311	0.016411	0.867427
42. H	–6.029493	–0.454992	1.735804
43. C	–6.171177	–0.146931	–0.390386
44. H	–7.084218	–0.737338	–0.479724
45. C	–5.608224	0.439557	–1.529469
46. H	–6.083748	0.294872	–2.499218
47. C	–4.438937	1.217595	–1.434004
48. C	–3.757708	1.013883	2.385277
49. H	–2.662339	1.015198	2.245674
50. C	–4.092643	–0.099072	3.401620

51. H	–5.148829	–0.056574	3.712752
52. H	–3.483413	0.034594	4.308570
53. H	–3.887722	–1.098038	2.986361
54. C	–4.177068	2.398239	2.952739
55. H	–3.802780	3.223119	2.330354
56. H	–3.772549	2.531933	3.968651
57. H	–5.275055	2.468494	3.001343
58. C	–3.787900	1.801155	–2.687911
59. H	–3.067526	2.576746	–2.381313
60. C	–3.002291	0.690448	–3.432158
61. H	–2.213525	0.259016	–2.797733
62. H	–2.536718	1.095899	–4.343952
63. H	–3.683731	–0.123940	–3.726454
64. C	–4.817164	2.482418	–3.621465
65. H	–5.480576	1.746722	–4.101080
66. H	–4.294256	3.026446	–4.423096
67. H	–5.442523	3.197433	–3.064855
68. C	1.158254	–1.872141	0.193179
69. N	1.924154	–2.531514	1.144031
70. C	1.932488	–3.912125	0.910133
71. H	2.474791	–4.591156	1.554916
72. C	1.160983	–4.134346	–0.200754
73. H	0.925485	–5.045278	–0.733645
74. N	0.692480	–2.883823	–0.640534
75. C	2.580393	–1.860370	2.248787
76. C	1.862611	–1.714290	3.455804
77. C	2.522128	–1.081983	4.524712
78. H	2.011048	–0.962299	5.480073
79. C	3.831262	–0.603739	4.377237
80. H	4.330006	–0.123345	5.220068
81. C	4.505101	–0.739725	3.156048
82. H	5.521378	–0.357658	3.058834
83. C	3.893918	–1.377356	2.060605
84. C	0.422265	–2.194813	3.599367
85. H	0.121513	–2.679172	2.656126
86. C	–0.530882	–0.994761	3.829965
87. H	–0.304813	–0.489512	4.783231
88. H	–1.570609	–1.349343	3.867266
89. H	–0.438903	–0.261286	3.015511
90. C	0.293540	–3.252735	4.724215
91. H	1.001598	–4.081165	4.564780
92. H	–0.728182	–3.663602	4.746811
93. H	0.503775	–2.812924	5.711816
94. C	4.636380	–1.532547	0.735121
95. H	3.976522	–2.061560	0.028359
96. C	4.962362	–0.149473	0.119275
97. H	4.044922	0.437818	–0.025094
98. H	5.456411	–0.273815	–0.857191
99. H	5.641511	0.423944	0.771741
100. C	5.909240	–2.397849	0.914170

101. H	6.644850	–1.894557	1.561515
102. H	6.385947	–2.582045	–0.061523
103. H	5.661455	–3.368105	1.373459
104. C	0.100009	–2.641976	–1.943365
105. C	–1.299008	–2.755473	–2.103538
106. C	–1.814794	–2.511382	–3.388660
107. H	–2.884651	–2.608626	–3.565198
108. C	–0.971878	–2.143135	–4.446202
109. H	–1.395222	–1.953871	–5.433646
110. C	0.411460	–2.038821	–4.252017
111. H	1.053657	–1.768744	–5.090718
112. C	0.983507	–2.300951	–2.993305
113. C	–2.202435	–3.200289	–0.955903
114. H	–1.683664	–2.969129	–0.009921
115. C	–3.565305	–2.468802	–0.938154
116. H	–3.436500	–1.378603	–0.988500
117. H	–4.103363	–2.703655	–0.006607
118. H	–4.208122	–2.783596	–1.776287
119. C	–2.410358	–4.738322	–1.023280
120. H	–2.902754	–5.014706	–1.969465
121. H	–3.042766	–5.077421	–0.187554
122. H	–1.452529	–5.277941	–0.974071
123. C	2.503630	–2.294746	–2.814093
124.H	2.733678	–2.566874	–1.772142
125. C	3.134492	–0.905944	–3.070667
126. H	2.951621	–0.572275	–4.104580
127. H	4.224713	–0.961261	–2.921808
128. H	2.728875	–0.150395	–2.381225
129. C	3.144890	–3.385349	–3.711885
130. H	2.670131	–4.364105	–3.537438
131. H	4.221185	–3.471995	–3.494829
132. H	3.030798	–3.140166	–4.779966

References

[1] a) Hintermann, L. *Beilstein J. Org. Chem.* **2007**, *3*, No. 22; b) Jafarpour, L.; Stevens, E. D.; Nolan, S. P. *J. Organomet. Chem.* **2000**, *606*, 49–54.

[2] Yakelis, N. A.; Bergman, R. G. *Organometallics* **2005**, *24*, 3579–3581.

[3] Cosier, J.; Glazer, A. M. *J. Appl. Cryst.* **1986**, *19*, 105–107.

[4] CrysAlisPro, Agilent Technologies, Version 1.171.35.8.

[5] Palatinus, L.; Chapuis, G. *J. Appl. Cryst.* **2007**, *40*, 786–790.

[6] a) Sheldrick, G. M. *Acta Cryst.* **2008**, A64, 112–122; b) Sheldrick, G. M. *Acta Cryst.* **1990**, A46, 467–473; c) SHELX2013, Programs for Crystal Structure Analysis (Release 2013), Sheldrick, G. M., University of Göttingen (Germany), 1998.

[7] Lubben, A. T.; McIndoe, J. S.; Weller, A. S. *Organometallics* **2008**, *27*, 3303–3306.

[8] Freed, J. H. *Spin Labeling: Theory and applications*, Berliner, L. J. (Ed), Academic Press, New York, 1976, Vol. 1, pp. 53–132.

[9] a) te Velde, G.; Bickelhaupt, F. M.; Baerends, E. J.; Fonseca Guerra, C.; van Gisbergen, S. J. A.; Snijders, J. G.; Ziegler, T. *J. Comput. Chem.* **2001**, *22*, 931−967; b) Fonseca Guerra, C.; Snijders, J. G.; te Velde, G.; Baerends, E. J. *Theor. Chem. Acc.* **1998**, *99*, 391−403; c) ADF2014.01, SCM, Theoretical Chemistry, Vrije Universiteit: Amsterdam, The Netherlands, http://www.scm.com.

[10] Becke, A. D. *Phys. Rev. A* **1988**, *38*, 3098–3100.

[11] Perdew, J. P. *Phys. Rev. B* **1986**, *33*, 8822–8824.

[12] Grimme, S.; Anthony, J.; Ehrlich, S.; Krieg, H. *J. Chem. Phys.* **2010**, *132*, 154104–154119.

[13] Versluis, L.; Ziegler, T.; *J. Chem. Phys.* **1988**, *88*, 322−328.
